# 18F-FDG-PET/CT-negative gastric cancer employs glutamine-based gluconeogenesis and fatty acid oxidation to support tumor growth

**DOI:** 10.1038/s41419-026-08662-9

**Published:** 2026-03-26

**Authors:** Jia Liu, Mingjie Xia, Zhexuan Zhao, Tian Gao, Yanzhao Qu, Qian Wang, Xiangdan Liu, Jianlan Du, Shunxin Han, Shiying Yang, Min Wei, Xin Jin, Yang Wang

**Affiliations:** 1 Key Laboratory of Molecular Epigenetics of the Ministry of Education (MOE), Northeast Normal University, Jilin, China; 2 Department of Gastric and Colorectal Surgery, General Surgery Center, The First Hospital of Jilin University, Jilin, China

**Keywords:** Cancer metabolism, Gastric cancer

## Abstract

Most tumors exhibit increased glucose uptake and reprogram metabolism to aerobic glycolysis to meet their demands for macromolecule biosynthesis and energy production. Consequently, PET/CT using 18F-2-fluoro-2-deoxy-D-glucose (18F-FDG-PET/CT) has been developed and is clinically utilized in cancer imaging diagnostics. However, numerous cancers demonstrate negative imaging during 18F-FDG-PET/CT detection, suggesting these cancers employ alternative metabolic rewiring. In this study, we discovered that 18F-FDG-PET/CT-negative gastric cancers coordinate glutamine-based gluconeogenesis and fatty acid oxidation to meet DNA and ATP demands, sustaining tumor growth despite low glucose uptake. PCK and CPT1A, the key enzymes which are responsible for remodeling the metabolism, were highly expressed in FDG-PET/CT-negative gastric cancers. Accordingly, PCK/CPT1A negatively correlated with 18F-FDG imaging levels and positively correlated with poorer clinical classifications. Mechanistically, PPARγ is highly expressed in FDG-PET/CT-negative cells and drives the transcription of the PCK and genes. Pharmacological inhibition of the PCK/CPT1A significantly suppressed tumor growth in 18F-FDG-PET/CT-negative gastric cancers, as demonstrated in both cell-derived xenograft (CDX) and patient-derived xenograft (PDX) models. Together, these results highlight the heterogeneity of tumor cells from metabolic perspective, and identify PCK/CPT1A as a target for metabolic reprogramming and precision therapy of 18F-FDG-PET/CT-negative cancers.

## Introduction

Metabolic reprogramming is a hallmark of tumor cells [1–4]. The Warburg effect, also known as aerobic glycolysis [2], is the most well-characterized phenomenon associated with metabolic reprogramming in tumor cells. Typically, glycolysis is a physiological response to hypoxia in normal tissues. However, in the 1920s, Otto Warburg observed that cancer cells in tumor slices and ascites constitutively take up glucose and switch glucose metabolism from oxidative phosphorylation to glycolysis. As a result, these cells produce lactate even under normal oxygen concentrations. This shift in glucose metabolism to glycolysis allows glycolytic intermediates to supply glycolytic shunts, thereby fueling the synthesis of macromolecules such as DNA, lipids, and proteins [2]. Therefore, aerobic glycolysis meets the biosynthetic demands required to support the rapid and sustainable proliferation of cancer cells. This phenomenon has been observed in various types of cancer cells and tumors to date [5–7].

Based on the phenomenon of aerobic glycolysis, 18F-2-fluoro-2-deoxy-D-glucose (18F-FDG), a glucose analog, has become the most widely used radiotracer for the detection of malignancies in combination with positron emission tomography (PET)/computed tomography (CT). Briefly, 18F-FDG is intravenously injected into the patient and is then rapidly taken up by tumor cells. It is subsequently phosphorylated to form 6-phosphate FDG, which cannot be further metabolized and thus accumulates continuously within cancer cells. This accumulation can be imaged using PET/CT to indicate cancer focus. Specifically, 18F-FDG-PET/CT is capable of detecting distant metastases and thereby enhances the accuracy and specificity of tumor diagnosis and analysis. To date, 18F-FDG-PET/CT has been widely applied for tumor diagnosis, staging, treatment monitoring, and evaluation of various types of malignant tumors [8–12].

Gastric cancer ranks fifth in incidence among all malignant tumors worldwide, particularly in Asia, with a fatality rate exceeding 75% [13, 14]. Early screening, preoperative staging of high-risk groups, and accurate diagnosis, including the use of 18F-FDG-PET/CT, are the primary approaches to reducing the incidence and mortality of gastric cancer [15, 16]. However, patients with gastric cancer often present with negative findings on 18F-FDG-PET/CT imaging, which significantly limits the diagnostic utility of this modality in gastric cancer [17, 18]. To facilitate treatment, gastric cancer has many clinicopathologic classifications, among which the most commonly used are the Lauren classification and the World Health Organization (WHO) classification [19]. However, the diffuse type of gastric cancer (from the Lauren classification) and gastric signet-ring cell carcinoma (from the WHO classification) are often more aggressive and frequently exhibit negative 18F-FDG-PET/CT findings [18]. Given that 18F-FDG-PET/CT is based on the principle of aerobic glycolysis, it is reasonable to question whether 18F-FDG-PET/CT-negative gastric cancer does not utilize aerobic glycolysis. If so, we are prompted to investigate which alternative metabolic pathways are employed by 18F-FDG-PET/CT-negative gastric cancer and how these pathways support tumor growth.

In this study, we demonstrate that 18F-FDG-PET/CT-negative gastric cancer cells rely on glutamine-based gluconeogenesis and fatty acid oxidation to fulfill their metabolic requirements for macromolecule biosynthesis and energy production. This metabolic strategy allows these cancer cells to maintain robust proliferative capacity despite low glucose uptake. PCK and CPT, key enzymes in gluconeogenesis and fatty acid oxidation, respectively, are highly expressed in 18F-FDG-PET/CT-negative gastric cancer cells and play a critical role in remodeling their metabolism. Moreover, transcription factor PPARγ is highly expressed in FDG-PET/CT-negative cells and drives the transcription of the PCK and CPT1A. Pharmacological inhibition of the PCK/CPT axis significantly suppresses the growth of 18F-FDG-PET/CT-negative gastric tumors. Collectively, our findings reveal a novel metabolic reprogramming pathway and provide potential therapeutic targets for the treatment of 18F-FDG-PET/CT-negative gastric cancer.

## Results

### Metabolic remodeling in 18F-FDG-PET/CT-negative gastric cancer extends beyond aerobic glycolysis

**Fig. 1:**
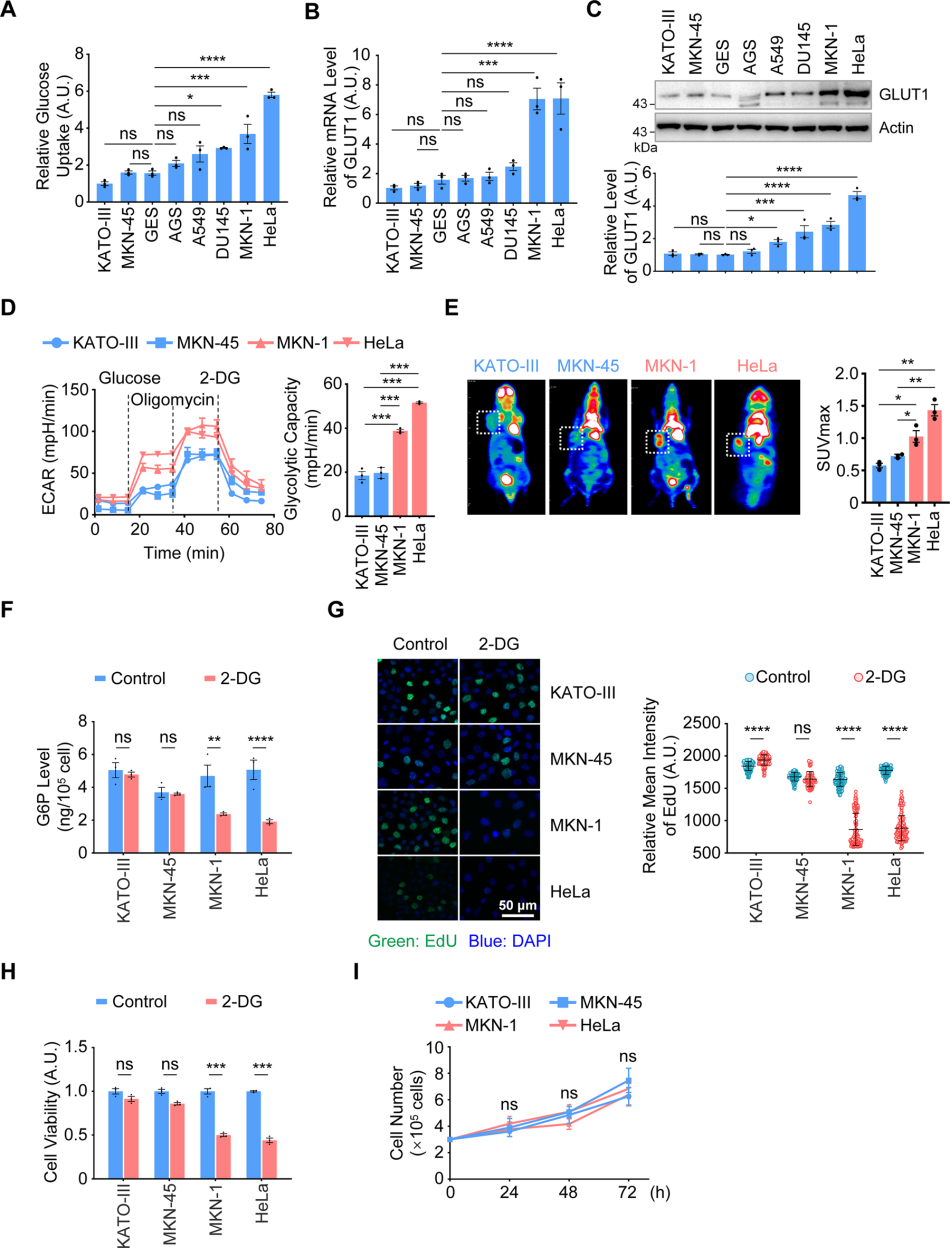
The 18F-FDG-PET/CT-negative gastric cancer remodels metabolism beyond aerobic glycolysis. **A**, **C** Assay of Glucose Uptake and GLUT1 Expression. Glucose uptake was measured in KATO-III, MKN-45, GES, AGS, A549, DU145, MKN-1, and HeLa cell lines using the Glucose Assay Kit (**A**). The mRNA (**B**) and protein (**C**) levels of GLUT1 were analyzed by RT-qPCR and WB, respectively. Data are presented as mean ± SD (*n* = 3). Statistical analysis was performed using one-way ANOVA: ^*^P < 0.05; ^***^P < 0.001; ^****^P < 0.0001. ns, not significant. A.U., arbitrary units.**D** Detection of ECAR. KATO-III, MKN-45, MKN-1, and HeLa cell lines were seeded in a Seahorse XFp analyzer and sequentially treated with glucose, oligomycin, and 2-DG. Real-time ECAR under different conditions was plotted (left panel), and glycolytic capacity was quantified (right panel). Data are presented as mean ± SD (*n* = 3). Statistical analysis was performed using one-way ANOVA: ^***^P < 0.001. **E** Xenograft Tumors Examined by 18F-FDG-PET/CT. KATO-III, MKN-45, MKN-1, and HeLa cell lines were injected into athymic nude mice to form xenograft tumors, which were then scanned using micro-PET/CT. Representative images are shown (left panel), and SUV values for FDG uptake in tumor regions were quantified (right panel). Data are presented as mean ± SD (*n* = 3). Statistical analysis was performed using one-way ANOVA: ^*^P < 0.05; ^**^P < 0.01. **F** Evaluation of G6P Levels. KATO-III, MKN-45, MKN-1, and HeLa cell lines were treated with or without 2-DG (5 mM) for 48 h and then subjected to G6P level analysis using ELISA. Data are presented as mean ± SD (n = 3). Statistical analysis was performed using two-way ANOVA: ^**^P < 0.01; ^****^P < 0.0001; ns, not significant. **G** Assay for DNA Synthesis. KATO-III, MKN-45, MKN-1, and HeLa cell lines were treated with or without 2-DG (5 mM) for 48 h and stained with EdU (green) and DAPI (blue). DNA synthesis was analyzed by immunofluorescence. Representative images are shown (left panel), and EdU intensity was quantified (right panel). Data are presented as mean ± SD (*n* = 3). Statistical analysis was performed using two-way ANOVA: ^****^P < 0.0001; ns, not significant. **H** Analysis of Cell Viability. KATO-III, MKN-45, MKN-1, and HeLa cell lines were treated with or without 2-DG (5 mM) for 48 h and then subjected to cell viability analysis using the CCK8 Assay Kit. Data are presented as mean ± SD (*n* = 3). Statistical analysis was performed using two-way ANOVA: ^***^P < 0.001; ns, not significant. **I** Functional Analysis of Cancer Cell Proliferation. KATO-III, MKN-45, MKN-1, and HeLa cell lines were cultured for 72 h, and cell proliferation curves were generated. Data are presented as mean ± SD (*n* = 3). Statistical analysis was performed using one-way repeated measures ANOVA: ns, not significant.

To elucidate the metabolic reprogramming mode of 18F-FDG-PET/CT-negative gastric cancer, identifying cell lines that mimic the negative 18F-FDG-PET/CT imaging phenotype is a top priority. Given that 18F-FDG-PET/CT imaging relies on high glucose uptake and glycolysis [18], we evaluated four gastric cancer cell lines and the gastric epithelial cell line GES for glucose uptake. We observed significant variations in glucose uptake among these cell lines (Fig. 1A). Initially, we selected KATO-III and MKN-45, which exhibited lower glucose uptake compared to the normal cell line GES, as the experimental models for studying 18F-FDG-PET/CT-negative gastric cancer. Meanwhile, MKN-1 and HeLa, which had higher glucose uptake than GES, were chosen as the control group (Fig. 1A). Additionally, we measured the expression of GLUT1, a marker directly reflecting glucose uptake capacity, and found that GLUT1 levels in KATO-III and MKN-45 were significantly lower than those in the control cells (Fig. 1B, C). Subsequently, we assessed the glycolytic capacity of these gastric cancer cells by analyzing their extracellular acidification rate (ECAR). The results showed that the ECAR levels, glycolytic capacity, and expression of key glycolytic enzymes in KATO-III and MKN-45 cells were notably lower than those in the control cells (Figs. 1D and S1). These findings indicated that KATO-III and MKN-45 cells indeed have lower glucose uptake and glycolytic capacity, making them suitable models for studying 18F-FDG-PET/CT-negative gastric cancer. Next, we subcutaneously implanted these cells into nude mice. After tumor formation, micro-PET/CT scans revealed that KATO-III and MKN-45 cells yielded negative 18F-FDG-PET/CT results, whereas positive results were observed for MKN-1 and HeLa (Fig. 1E). Concurrently, the standard uptake value (SUV) of FDG absorbed by the tumorigenic regions of KATO-III and MKN-45 was significantly lower than that of MKN-1 and HeLa (Fig. 1E). Collectively, these results further confirm that the KATO-III and MKN-45 cell lines are appropriate models for mimicking 18F-FDG-PET/CT-negative imaging.

The glycolytic intermediate glucose-6-phosphate (G6P) can enter the pentose phosphate pathway (PPP), which produces NADPH and ribose-5-phosphate (R5P). These molecules are critical building blocks for nucleotide biogenesis and are thus essential for DNA synthesis and subsequent cell proliferation [20]. After confirming reduced glucose uptake and diminished glycolysis in 18F-FDG-PET/CT-negative gastric cancer cells, we hypothesized that the levels of the glycolytic intermediate G6P and intracellular nucleic acid synthesis might be decreased. However, further experiments revealed that treatment with the glycolysis inhibitor 2-Deoxy-D-glucose (2-DG) did not significantly reduce the levels of G6P or DNA synthesis in KATO-III and MKN-45 cells, whereas these levels were significantly decreased in control cells (Fig. 1F, G). More importantly, 2-DG barely affected the proliferation rates of KATO-III and MKN-45 cells compared to control cells (Fig. 1H), and the proliferation rates of KATO-III and MKN-45 did not differ significantly from those of the control group (Fig. 1I). Collectively, these data suggest that 18F-FDG-PET/CT-negative gastric cancer cells remodel their metabolism beyond canonical aerobic glycolysis, and alternative metabolic pathways may compensate for glycolysis to support cancer cell proliferation.

### Glutamine-based gluconeogenesis supports macromolecule synthesis in 18F-FDG-PET/CT-negative gastric cancer

**Fig. 2:**
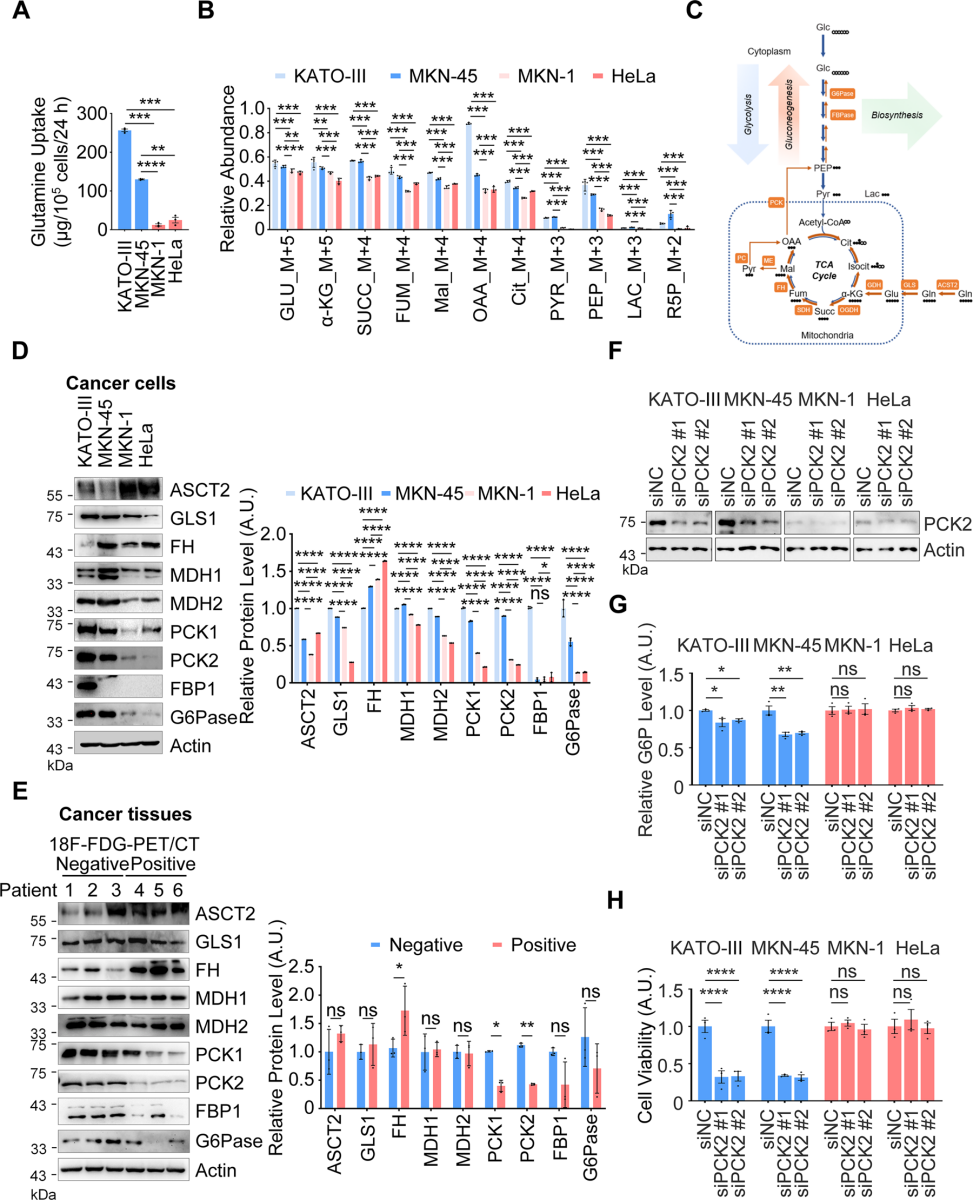
Glutamine-based gluconeogenesis back up macromolecule synthesis in 18F-FDG-PET/CT-negative gastric cancer. **A** Assay for Glutamine Uptake. KATO-III, MKN-45, MKN-1, and HeLa cell lines were cultured for 24 h, and the supernatants were analyzed for the level of glutamine. Data are presented as mean ± SD (*n* = 3). Statistical analysis was performed using one-way ANOVA: ^**^P < 0.01; ^***^P < 0.001; ^****^P < 0.0001. **B** Evaluation of Major Metabolites in Glutamine Metabolic Flux. KATO-III, MKN-45, MKN-1, and HeLa cell lines were cultured in the presence of [^13^C_5_]-glutamine for 24 h prior to metabolic flux analysis by UHPLC-HRMS. Metabolites analyzed include glutamate (GLU), α-ketoglutarate (α-KG), succinate (SUCC), fumarate (FUM), malate (MAL), citrate (CIT), pyruvate (PYR), oxaloacetate (OAA), phosphoenolpyruvate (PEP), lactate (LAC), and ribulose-5-phosphate (R5P). Data are presented as mean ± SD (*n* = 5). Statistical analysis was performed using one-way ANOVA: ^**^P < 0.01; ^***^P < 0.001; ^****^P < 0.0001. **C** Metabolic flow pattern of glutamine labeled with [^13^C_5_]. Evaluation of the major metabolic enzymes in glutamine-derived gluconeogenesis flux. KATO-III, MKN-45, MKN-1 and HeLa cell lines (**D**) and 18F-FDG-PET/CT-negative and positive gastric cancer tissues (**E**) were subjected to metabolic enzyme expression assays by WB, respectively. Representative images and quantification of proteins were displayed. Mean ± SD, *n* = 3. Two-way ANOVA: ^*^P < 0.05; ^**^P < 0.01; ^****^P < 0.0001; ns, not significant. **F** Evaluation of PCK2 Knockdown Effect. KATO-III, MKN-45, MKN-1, and HeLa cell lines with endogenous PCK2 depletion were analyzed for PCK2 protein levels by WB. **G** Evaluation of G6P Levels. KATO-III, MKN-45, MKN-1, and HeLa cell lines were depleted for endogenous PCK2 for 48 h. G6P levels were measured by ELISA. Data are presented as mean ± SD (*n* = 3). Statistical analysis was performed using two-way ANOVA: ^*^P < 0.05; ^**^P < 0.01; ns, not significant. A.U., arbitrary units. **H** Analysis of Cell Viability. KATO-III, MKN-45, MKN-1, and HeLa cell lines were depleted for endogenous PCK2 for 48 h. Cell viability was assessed using the CCK8 Assay Kit. Data are presented as mean ± SD (*n* = 3). Statistical analysis was performed using two-way ANOVA: ^****^P < 0.0001; ns, not significant.

Given the crucial role of glycolysis, we hypothesized that 18F-FDG-PET/CT-negative gastric cancer cells may engage in gluconeogenesis, the reverse process of glycolysis, to compensate for the reduced glycolytic flux, thereby providing the necessary macromolecular precursors to support rapid cell proliferation. Amino acids are well-established as the key precursors for initiating gluconeogenesis [21–23]. Therefore, we employed high-performance liquid chromatography (HPLC) to measure the uptake levels of amino acids. The data revealed that the uptake of glutamine by the 18F-FDG-PET/CT-negative gastric cancer cell lines KATO-III and MKN-45 was significantly higher than that of the other 19 amino acids and was also higher than that in control cells (Figs. 2A and S2A). This suggests that glutamine may play a pivotal role in 18F-FDG-PET/CT-negative gastric cancer cells.

To further elucidate the role of glutamine in these cancer cells, we cultured the cells with isotope-labeled [^13^C_5_]-glutamine. Metabolic flux analysis showed that the levels of ^13^C-labeled metabolites, including glutamate (GLU), α-ketoglutarate (α-KG), succinate (SUCC), fumarate (FUM), malate (MAL), citrate (CIT), pyruvate (PYR), oxaloacetate (OAA), phosphoenolpyruvate (PEP), lactate (LAC), and ribulose-5-phosphate (R5P), were significantly higher in the 18F-FDG-PET/CT-negative gastric cancer cells (KATO-III and MKN-45) compared to control cells (Fig. 2B). Based on these isotope labeling results, we constructed a metabolic flux diagram for glutamine (Fig. 2C), which indicated that glutamine enters gluconeogenesis via the mitochondrial tricarboxylic acid (TCA) cycle in 18F-FDG-PET/CT-negative gastric cancer cells.

To elucidate the mechanism underlying the initiation of glutamine-based gluconeogenesis in 18F-FDG-PET/CT-negative gastric cancer, we examined the expression levels of key gluconeogenic enzymes (e.g., Fructose-1,6-BisPhosphatase (FBPase) and Glucose-6-Phosphatase (G6Pase)) in both 18F-FDG-PET/CT-negative and -positive gastric cancer cells and tissues. The results demonstrated that the expression of key enzymes, particularly PCK1/2, was significantly higher in KATO-III and MKN-45 cells, as well as in 18F-FDG-PET/CT-negative gastric cancer tissues, compared to control cells and tissues (Fig. 2D, E). While the expression levels of other key enzymes were not significantly different between PET-positive and -negative groups, like FBPase or G6Pase (Fig. 2D, E). Next, we assessed the impact of glutamine-based gluconeogenesis on the proliferation of 18F-FDG-PET/CT-negative gastric cancer cells. Inhibition of PCK1/2 using the specific inhibitor 3-Mercaptopicolinic acid (3-MPA), or knockdown of PCK2 expression, significantly reduced the levels of G6P and DNA in KATO-III and MKN-45 cells (Figs. 2F, G, and S2B). Correspondingly, these treatments also inhibited the proliferation of these cells (Figs. 2H and S2C). Collectively, these findings suggest that 18F-FDG-PET/CT-negative gastric cancer cells utilize glutamine-based gluconeogenesis to support the synthesis of macromolecules required for cell proliferation.

### Fatty acid oxidation supports energy generation in 18F-FDG-PET/CT-negative gastric cancer

**Fig. 3:**
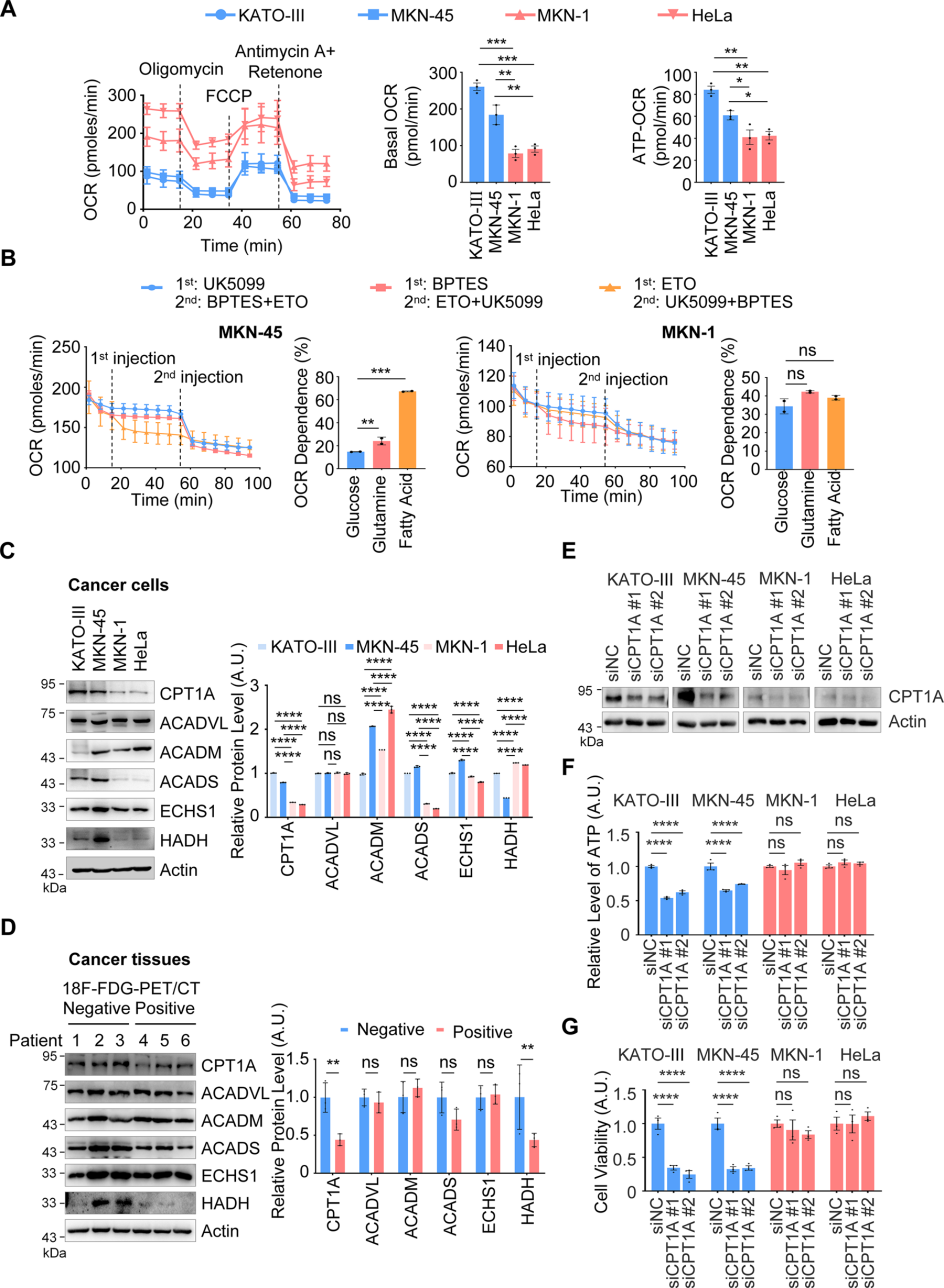
The expression of PCK2 or CPT1A negatively associate with 18F-FDG-PET/CT in gastric cancer. **A** Detection of OCR. KATO-III, MKN-45, MKN-1, and HeLa cell lines were seeded in a Seahorse XFp analyzer and sequentially treated with oligomycin, FCCP, and antimycin A/rotenone. Real-time OCR under different conditions was plotted (left panel), and the rates of overall oxygen consumption (middle panel) and oxygen consumption for ATP production (right panel) were quantified. Data are presented as mean ± SD (*n* = 3). Statistical analysis was performed using one-way ANOVA: ^*^P < 0.05; ^**^P < 0.01; ^***^P < 0.001. (**B**) Evaluation of the Contribution Ratio of Different Nutrients to OCR. MKN-45 and MKN-1 cell lines were treated with specific inhibitors of different metabolic pathways prior to OCR assessment. Real-time OCR under different conditions was plotted (left panel), and associated quantifications were shown (right panel). The inhibitors used were: UK5099 (30 µM, inhibitor of glucose metabolism), BPTES (40 µM, inhibitor of glutamine metabolism), and ETO (20 µM, inhibitor of fatty acid oxidation metabolism). Data are presented as mean ± SD (*n* = 3). Statistical analysis was performed using one-way ANOVA: ^**^P < 0.01; ^***^P < 0.001. Evaluation of the major metabolic enzymes in fatty acid oxidation. KATO-III, MKN-45, MKN-1 and HeLa cell lines (**C**) and 18F-FDG-PET/CT-negative and -positive gastric cancer tissues (**D**) were subjected to metabolic enzyme expression assays by WB, respectively. Mean ± SD, *n* = 3. Two-way ANOVA: ^**^P < 0.01; ^****^P < 0.0001; ns, not significant. **E** Evaluation of the knockdown effect of CPT1A. KATO-III, MKN-45, MKN-1 and HeLa cell lines depleted for endogenous CPT1A were subjected to examination of CPT1A protein levels by WB. **F** Evaluation of ATP Levels. KATO-III, MKN-45, MKN-1, and HeLa cell lines were depleted for endogenous CPT1A. ATP levels were measured using the ATP Assay Kit. Data are presented as mean ± SD (*n* = 3). Statistical analysis was performed using one-way ANOVA: ^****^P < 0.0001; ns, not significant. **G** Analysis of Cell Viability. KATO-III, MKN-45, MKN-1, and HeLa cell lines were treated as described in (**E**) and then subjected to cell viability analysis using the CCK8 Assay Kit. Data are presented as mean ± SD (*n* = 3). Statistical analysis was performed using one-way ANOVA: ^****^P < 0.0001; ns, not significant.

Given that gluconeogenesis is an energy-consuming pathway, the question arises as to how 18F-FDG-PET/CT-negative gastric cancer cells with low glucose uptake meet their energy demands. Initially, we measured the mitochondrial oxygen consumption rate (OCR), a key indicator of ATP generation. The results showed that, compared to control cells, the basal OCR was significantly increased in 18F-FDG-PET/CT-negative gastric cancer cells (KATO-III and MKN-45), with a corresponding increase in ATP production (Fig. 3A). These findings suggest that despite low glucose uptake and glycolysis, these cancer cells exhibit robust mitochondrial ATP generation.To investigate which nutrients contribute to mitochondrial ATP generation in 18F-FDG-PET/CT-negative gastric cancer cells, we used specific inhibitors targeting different metabolic pathways: the glucose metabolism inhibitor UK5099, the glutamine metabolism inhibitor BPTES, and the fatty acid oxidation inhibitor Etomoxir (ETO). The results indicated that ETO significantly decreased OCR in KATO-III and MKN-45 cells but not in control cells (Figs. 3B, and S3, S4A). This suggests that fatty acid oxidation is a major contributor to mitochondrial ATP generation in 18F-FDG-PET/CT-negative gastric cancer cells.

To elucidate the mechanism underlying the initiation of fatty acid oxidation in 18F-FDG-PET/CT-negative gastric cancer, we examined the expression levels of key enzymes (e.g., enoyl-CoA hydratase, short chain (ECHS) and hydroxyacyl-CoA dehydrogenase (HADH)) involved in this process in both 18F-FDG-PET/CT-negative gastric cancer cells and tissues. The results revealed that the expression of key enzymes, particularly CPT1A, was significantly higher in KATO-III and MKN-45 cells, as well as in 18F-FDG-PET/CT-negative gastric cancer tissues, compared to control cells and tissues (Fig. 3C, D). While the expression levels of other key enzymes were not significantly different between PET-positive and negative groups, like ECHS or HADH (Fig. 3C, D). Next, we assessed the impact of fatty acid oxidation on the proliferation of 18F-FDG-PET/CT-negative gastric cancer cells. Inhibition of CPT1A using the specific inhibitor ETO, or knockdown of CPT1A expression, significantly reduced ATP levels in KATO-III and MKN-45 cells (Figs. 3E, F and S4B). Correspondingly, these treatments also inhibited the proliferation of these cells (Figs. 3G and S4C). Collectively, these findings suggest that 18F-FDG-PET/CT-negative gastric cancer cells utilize fatty acid oxidation for ATP generation and to support cell proliferation.

### PCK2 and CPT1A are enriched in and drive the progression of 18F-FDG- PET/CT-negative cancer

**Fig. 4:**
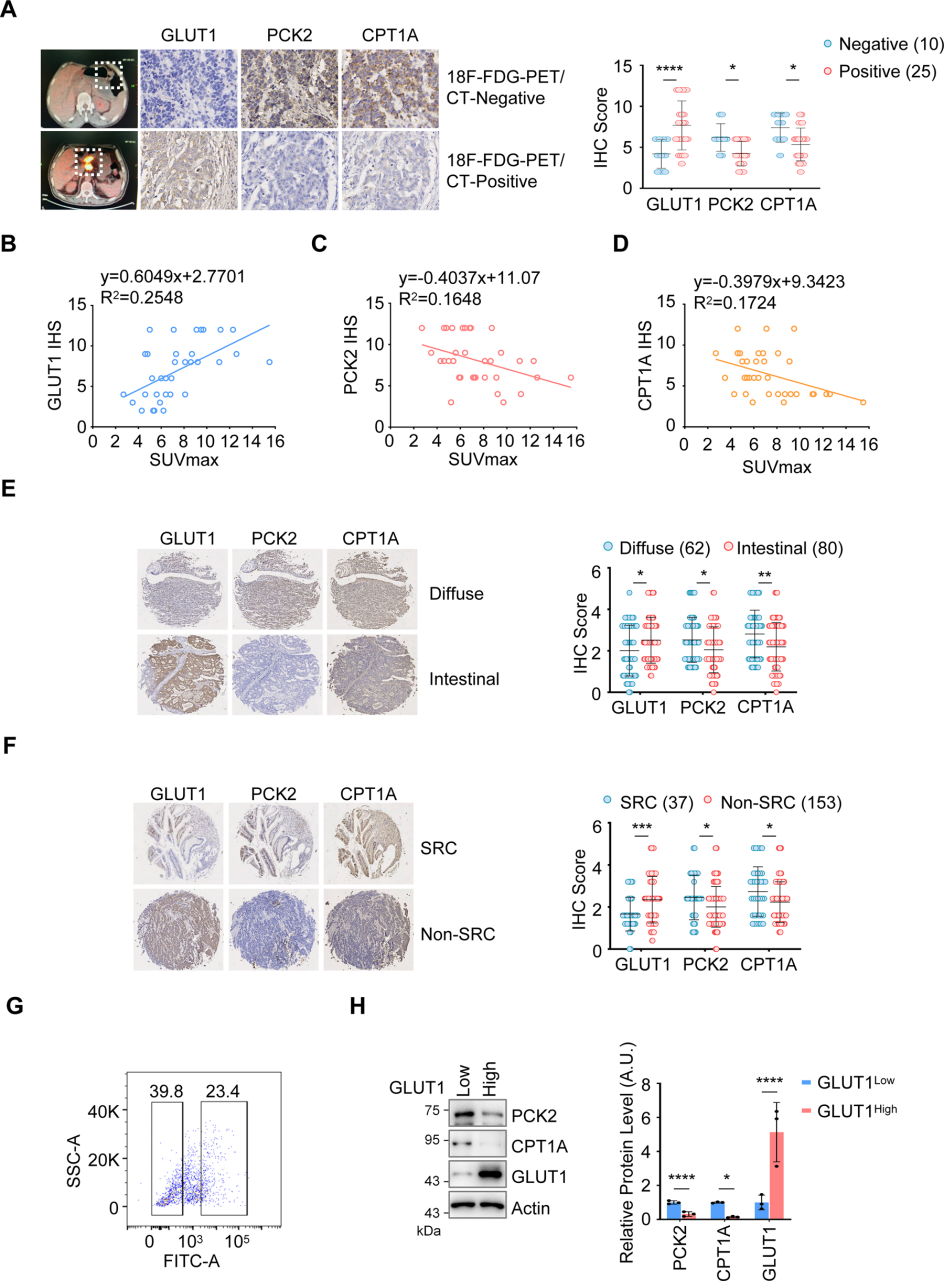
PCK2 and CPT1A are enriched in and drive the progression of 18F-FDG-PET/CT-negative cancer. **A** IHC Analysis of GLUT1, PCK2, and CPT1A in 18F-FDG PET-Negative and -Positive Gastric Cancer Tissues. Immunohistochemical (IHC) analysis was performed on 18F-FDG-PET-negative cancer tissues from 10 patients and 18F-FDG-PET-positive cancer tissues from 25 patients using antibodies against GLUT1, PCK2, and CPT1A. Representative images are shown (left panel). The staining intensity for GLUT1, PCK2, and CPT1A was scored and presented (right panel). IHC Analysis of GLUT1, PCK2, and CPT1A in a Gastric Carcinoma Tissue Microarray. A tissue microarray containing 190 cases of gastric cancer was subjected to IHC analysis to evaluate the levels of GLUT1 (**B**), PCK2 (**C**), and CPT1A (**D**). GLUT1, PCK2 and CPT1A staining were scored and subjected to correlation analysis with SUV values, respectively. The correlation analysis was determined by Pearson product moment correlation test. Mean ± SD, *n* = 35. **E** IHC Analysis of GLUT1, PCK2, and CPT1A in Gastric Cancer Subtypes According to the Lauren Classification. IHC analysis was performed on 62 cases of diffuse-type gastric cancer and 80 cases of intestinal-type gastric cancer according to the Lauren classification. Representative images are shown (left panel), and the quantification of GLUT1, PCK2, and CPT1A is presented (right panel). Data are expressed as mean ± SD (*n* = 62 or 80). Statistical analysis was performed using one-way ANOVA: ^*^P < 0.05; ^**^P < 0.01. **F** IHC Analysis of GLUT1, PCK2, and CPT1A in Gastric Cancer Subtypes According to the WHO Classification. IHC analysis was performed on 37 cases of gastric signet-ring cell carcinoma and 153 cases of non-signet-ring cell carcinoma according to the WHO classification. Representative images are shown (left panel), and the quantification of GLUT1, PCK2, and CPT1A is presented (right panel). Data are expressed as mean ± SD (*n* = 37 or 153). Statistical analysis was performed using one-way ANOVA: ^*^P < 0.05; ^***^P < 0.001. (G and H) Single cells from gastric cancer tissues were stained with an anti-GLUT1 antibody, sorted into GLUT1^high^ and GLUT1^low^ populations by flow cytometry (**G**), and analyzed for PCK2, CPT1A, and GLUT1 protein expression by WB (**H**). Representative images (left panel) and quantification of proteins (right panel) were displayed. Mean ± SD, *n* = 3. Two-way ANOVA: ^*^P < 0.05; ^****^P < 0.0001.

To further evaluate the roles of gluconeogenesis and fatty acid oxidation in 18F-FDG-PET/CT-negative gastric cancer tissues, we assessed the expression levels of GLUT1, PCK2, and CPT1A in 35 gastric cancer patients. These markers were used to indicate the levels of glycolysis, gluconeogenesis, and fatty acid oxidation, respectively. The patients underwent 18F-FDG-PET/CT imaging before surgery and were categorized into two groups: 18F-FDG-PET/CT-negative (SUV ≤ 2.5, *n* = 10) and 18F-FDG-PET/CT-positive (SUV > 2.5, *n* = 25). The results showed that GLUT1 expression was significantly lower in 18F-FDG-PET/CT-negative gastric cancer tissues compared to 18F-FDG-PET/CT-positive tissues (Fig. 4A). Conversely, the expression of PCK2 and CPT1A was higher in the 18F-FDG-PET/CT-negative tissues (Fig. 4A). Consistent with this, the expression of PCK2 and CPT1A showed a negative correlation with SUV values in gastric cancer tissues, whereas GLUT1 expression exhibited a positive correlation (Fig. 4B–D). These data indicate that the expression of PCK2 and CPT1A is indeed inversely correlated with 18F-FDG-PET/CT imaging.

The clinical diagnosis and treatment of gastric cancer rely heavily on established clinicopathological classifications, such as the Lauren and WHO systems. It has been reported that diffuse-type gastric cancer and gastric signet-ring cell carcinoma, in which classifications are often more aggressive, associated with shorter survival and higher metastatic potential, and frequently show negative 18F-FDG-PET/CT imaging [13, 14, 24]. To further examine the relationship between PCK2/CPT1A expression and prognosis in gastric cancer, we performed immunohistochemical staining on tissues from 190 gastric cancer patients. Compared with intestinal-type gastric cancer and non-signet-ring cell carcinoma, the expression of PCK2 and CPT1A was significantly elevated in diffuse-type gastric cancer and gastric signet-ring cell carcinoma (Fig. 4E, F). Conversely, GLUT1 expression was markedly reduced in these same subtypes (Fig. 4E, F). In other words, 18F-FDG-PET/CT-negative gastric cancers, which are associated with shorter patient survival, exhibit high expression of PCK2 and CPT1A. In contrast, PET/CT-positive tumors, linked to longer survival, show low expression of these metabolic enzymes. These findings collectively indicate that elevated expression of PCK2/CPT1A serves as a prognostic marker for poorer survival specifically in the context of low FDG-avidity gastric cancer. To further investigate the correlation between PCK2 or CPT1A and GLUT1, we analyzed a publicly available single-cell RNA-seq dataset of gastric cancer (GSE134520) [25] and confirmed that GLUT1^low^ tumor cells exhibit significantly higher expression of PCK2 and CPT1A compared to GLUT1^high^ cells (Fig. S5D, E). The results revealed a significant upregulation of PCK2 and CPT1A in gastric cancer cells with low GLUT1 expression. More importantly, we performed flow cytometry on single-cell suspensions obtained from fresh 18F-FDG-PET/CT-negative gastric cancer tissues using an anti-GLUT1 antibody, allowing us to separate them into GLUT1^high^ and GLUT1^low^ populations. Subsequent analysis confirmed elevated protein expression of PCK2 and CPT1A in the GLUT1^low^ group (Fig. 4G, H). Moreover, we further analysis of TCGA data across multiple additional cancer types demonstrated similar negative correlations between PCK2/CPT1A and GLUT1 expression in hepatocellular carcinoma (HCC) and kidney renal clear cell carcinoma (KIRC) (Fig. S6A–D), both of which have also been reported to exhibit 18F-FDG-PET/CT-negative imaging [26, 27]. Collectively, these data suggest that PCK2 and CPT1A are enriched in and drive the progression of 18F-FDG-PET/CT-negative cancer.

### PPARγ drives the transcription of PCK2 and CPT1A in 18F-FDG-PET/CT-negative gastric cancer

**Fig. 5:**
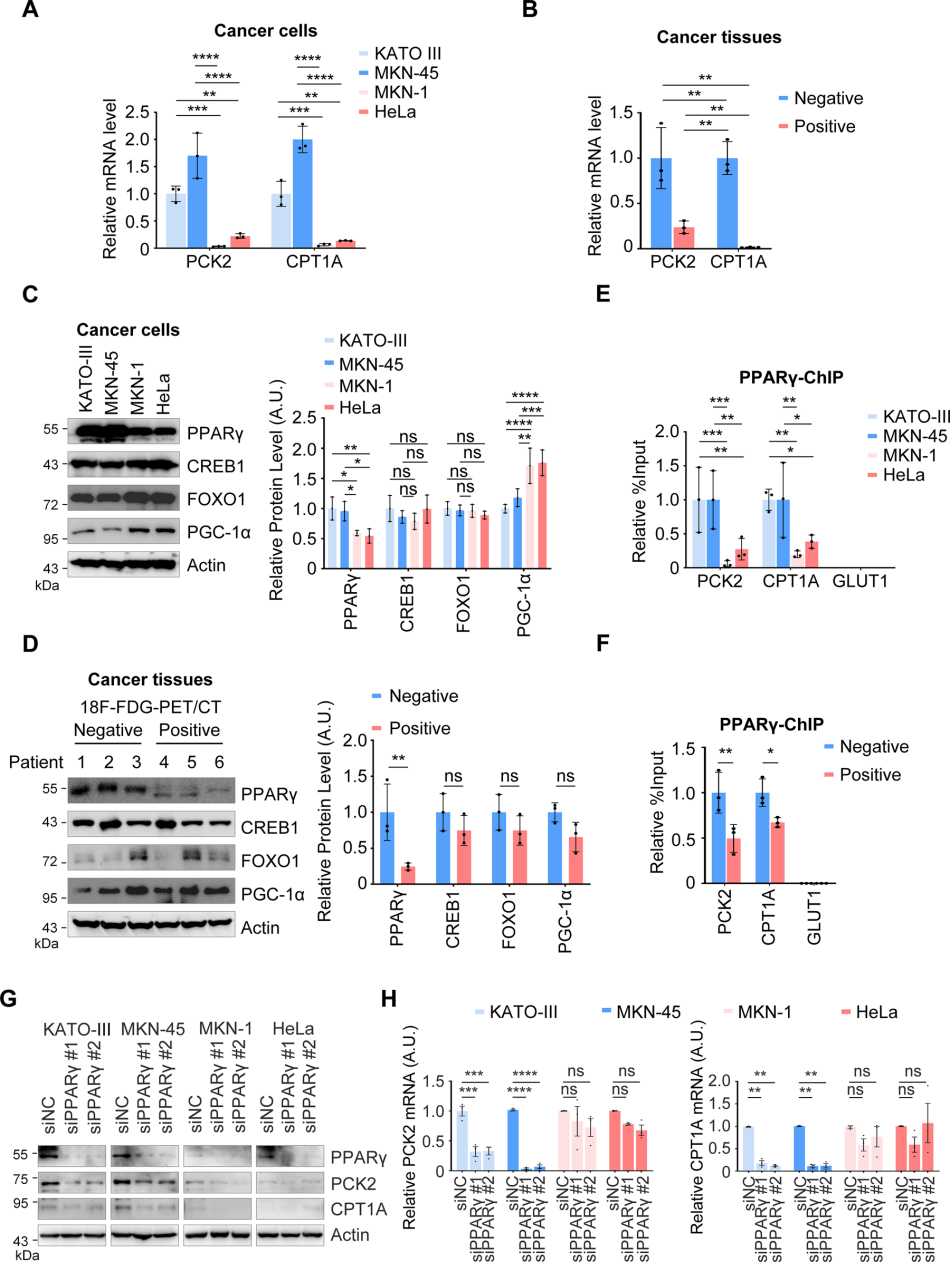
PPARγ upregulation drives the transcription of PCK2 and CPT1A in 18F-FDG-PET/CT-negative gastric cancer. Assay of PCK2 and CPT1A Expression. The mRNA levels of PCK2 and CPT1A were measured in KATO-III, MKN-45, MKN-1, HeLa cell lines (**A**) and 18F-FDG-PET/CT-negative and positive gastric cancer tissues (**B**) by RT-qPCR. Data are presented as mean ± SD (*n* = 3). Statistical analysis was performed using two-way ANOVA: ^**^P < 0.01; ^***^P < 0.001; ^****^P < 0.0001. A.U., arbitrary units. Assay of the Expression of Transcription Factors of PCK2 and CPT1A Genes. KATO-III, MKN-45, MKN-1 and HeLa cell lines (**C**) and 18F-FDG-PET/CT-negative gastric cancer tissues (**D**) were subjected to the expression assays of PPARγ, CREB1, FOXO1 and PGC-1α by WB. Representative images (left panel) and quantification of proteins (right panel) were displayed. Mean ± SD, *n* = 3; Two-way ANOVA: ^*^P < 0.05; ^**^P < 0.01; ^***^P < 0.001; ^****^P < 0.0001; ns, not significant. A.U., arbitrary units. **E**, **F** PPARγ Upregulation Drives the Transcription of PCK2 and CPT1A in 18F-FDG-PET/CT-negative Gastric Cancer. The binding of PPARγ to PCK2, CPT1A and GLUT1 gene promoter was evaluated by ChIP in KATO-III, MKN-45, MKN-1 and HeLa cell lines (**E**) and 18F-FDG-PET/CT-negative gastric cancer tissues (**F**). Data are presented as mean ± SD (*n* = 3). Statistical analysis was performed using two-way ANOVA: ^*^P < 0.05; ^**^P < 0.01; ^***^P < 0.001; ns, not significant. A.U., arbitrary units. **G**, **H** PPARγ Downregulation Inhibits PCK2 and CPT1A Expression. Evaluation of the knockdown effect of PPARγ. KATO-III, MKN-45, MKN-1 and HeLa cell lines depleted for endogenous PPARγ were subjected to examination of PPARγ, CPT1A and PCK2 protein levels by WB (**G**) and RT-qPCR (**H**). Data are presented as mean ± SD (*n* = 3). Statistical analysis was performed using two-way ANOVA: ^**^P < 0.01; ^***^P < 0.001; ^****^P < 0.0001; ns, not significant. A.U., arbitrary units.

We next investigated the mechanisms underlying the metabolic reprogramming mediated by PCK2 and CPT1A in 18F-FDG-PET/CT-negative gastric cancer. Given the specific overexpression of PCK2 and CPT1A in these tumors, we hypothesized that their expression might be regulated at the transcriptional level. First, we examined their mRNA levels and observed significant upregulation of both PCK2 and CPT1A in gastric cancer cells and tissues (Fig. 5A, B). We then analyzed key transcription factors known to regulate these genes, including CREB (cAMP Response Element-Binding Protein), FOXO1 (Forkhead Box Protein O1), PPARγ (Peroxisome Proliferator-Activated Receptor gamma), and PGC-1α (PPARγ Coactivator-1 alpha) [28–34]. Among these, only PPARγ protein levels were elevated in 18F-FDG-PET/CT-negative samples (Fig. 5C, D). To determine whether PPARγ directly regulates PCK2 and CPT1A transcription, we performed chromatin immunoprecipitation assays, which confirmed PPARγ binding to the promoters of PCK2 and CPT1A but not to the GLUT1 promoter (Fig. 5E, F). Furthermore, knockdown of PPARγ reduced the expression of both PCK2 and CPT1A (Fig. 5G, H). These results indicate that PPARγ upregulation drives the increased expression of PCK2 and CPT1A in 18F-FDG-PET/CT-negative gastric cancer.

### Targeting the PCK2/CPT1A axis significantly inhibits tumor growth in 18F-FDG-PET/CT-negative gastric cancer

**Fig. 6:**
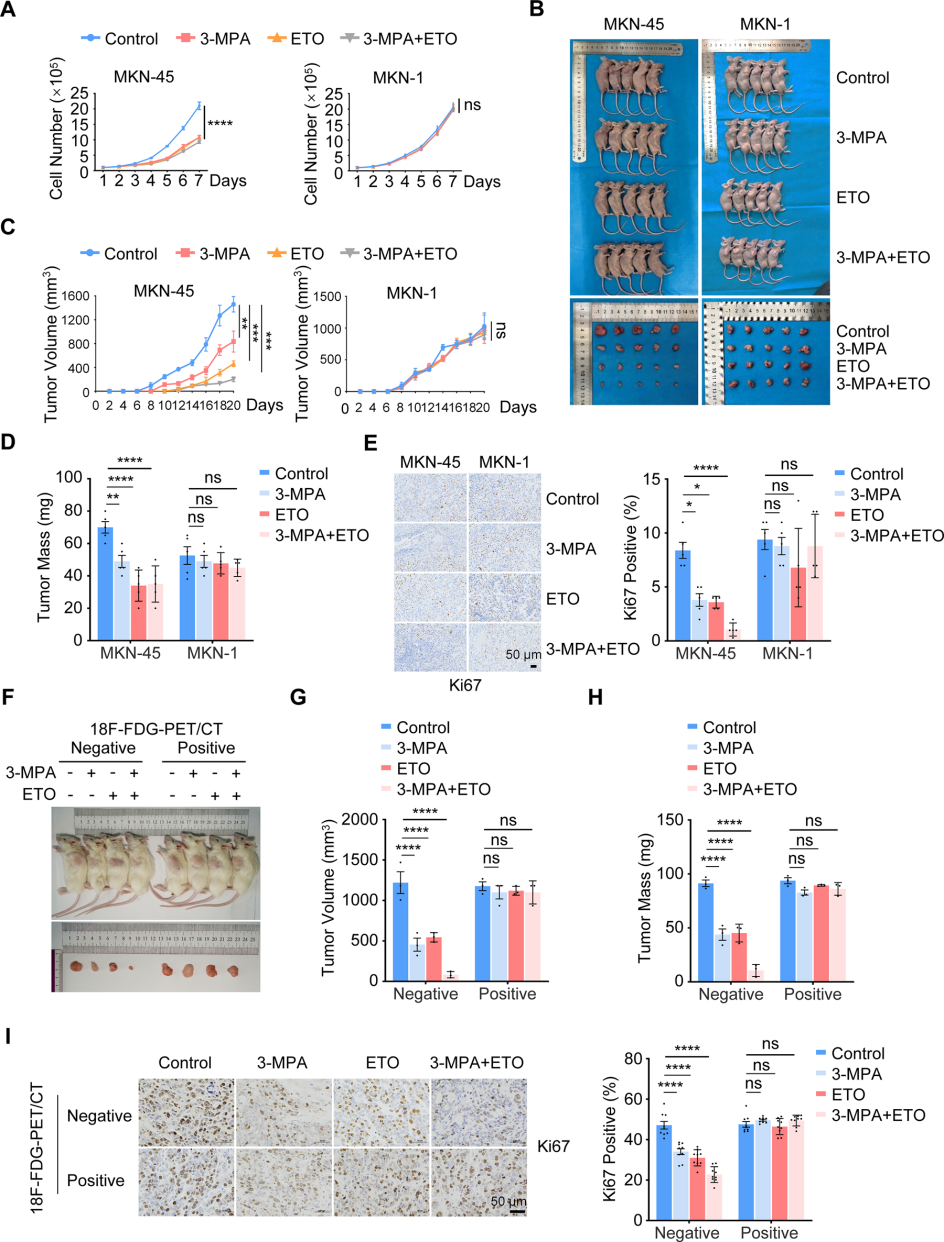
Targeting PCK/CPT1A axis slows down tumor growth of 18F-FDG-PET/CT-negative gastric cancer. **A** Analysis of the Effects of PCK2 and CPT1A on Cell Proliferation. Cell proliferation curves of MKN-45 and MKN-1 cell lines treated with 3-MPA (alone or in combination with ETO) were demonstrated. Data are presented as mean ± SD (*n* = 3). Statistical analysis was performed using two-way ANOVA: ^****^P < 0.0001; ns, not significant. **B**–**D** Assay for Tumor Formation. MKN-45 and MKN-1 cell lines were injected into athymic nude mice. The mice were intraperitoneally injected with PBS (10 mg/kg), 3-MPA (5 mg/kg), ETO (10 mg/kg), or a combination of 3-MPA and ETO every other day. After 22 days, the xenograft tumors were excised, and representative images are shown (**B**). Tumor volume (**C**) and mass (**D**) were quantified. Data are presented as mean ± SD (*n* = 5). Statistical analysis was performed using two-way ANOVA: ^**^P < 0.01; ^***^P < 0.001; ^****^P < 0.0001; ns, not significant. **E** Immunohistochemistry (IHC) Analysis of Cell Proliferation. Five pairs of mice injected with MKN-45 and MKN-1 cell lines were co-stained with Ki-67. Representative images of tumor tissues are shown (scale bars, 50 μm) (left panel), and the quantification of Ki-67 staining is presented (right panel). Data are presented as mean ± SD (*n* = 5). Statistical analysis was performed using two-way ANOVA: ^*^P < 0.05; ^****^P < 0.0001; ns, not significant. **F**, **H** Establishment and characterization of patient-derived xenograft (PDX) models of gastric cancer. Fresh tumor tissue from a gastric cancer patient was surgically implanted into the subcutaneous pocket of an immunodeficient mouse. Upon day 14, the mice were intraperitoneally injected with PBS (10 mg/kg), 3-MPA (5 mg/kg), ETO (10 mg/kg), or a combination of 3-MPA and ETO every other day. After 14 days, the xenograft tumors were excised, and representative images are shown (**F**). Tumor volume (**G**) and mass (**H**) were quantified. Data are presented as mean ± SD (*n* = 3). Statistical analysis was performed using two-way ANOVA: ^****^P < 0.0001; ns, not significant. **I** Immunohistochemistry (IHC) Analysis of Cell Proliferation. The mice injected with gastric cancer tissues were co-stained with Ki-67. Representative images of tumor tissues are shown (scale bars, 50 μm) (left panel), and the quantification of Ki-67 staining is presented (right panel). Data is presented as mean ± SD (three fields from each analyzed mouse). Statistical analysis was performed using two-way ANOVA: ^****^P < 0.0001; ns, not significant.

We next confirmed the role of metabolic reprogramming in the proliferation of 18F-FDG-PET/CT-negative gastric cancer cells. When the PCK inhibitor 3-MPA and the CPT1A inhibitor ETO were administered individually or in combination to inhibit gluconeogenesis and fatty acid oxidation, respectively, the proliferation of 18F-FDG-PET/CT-negative gastric cancer cells (MKN-45) was significantly impaired, whereas the proliferation of 18F-FDG-PET/CT-positive gastric cancer cells (MKN-1) remained unaffected (Fig. 6A). To further investigate the physiological functions of gluconeogenesis and fatty acid oxidation, we emplyed cell-derived xenografts (CDX) and injected equal numbers of MKN-45 and MKN-1 cells into mice. Tumor volume and mass were significantly reduced in mice injected with MKN-45 following treatment with 3-MPA or ETO, while no significant changes were observed in mice injected with MKN-1 cells (Fig. 6B–D). More importantly, the co-administration of 3-MPA and ETO further inhibited the proliferation of MKN-45 tumors in vivo (Fig. 6B–D). Subsequently, tumor xenografts isolated from mice injected with MKN-45 were subjected to histological analysis. The expression of Ki-67, a classic marker of cell proliferation, was significantly decreased following treatment with 3-MPA or ETO, confirming that inhibition of gluconeogenesis and fatty acid oxidation reduced tumor growth in 18F-FDG-PET/CT-negative gastric cancer (Fig. 6E). Importantly, further patient-derived xenografts (PDX) from both fresh 18F-FDG-PET/CT-positive and -negative gastric cancer tissues were employed to nude mice. On day 14 post-tumor inoculation, a combination of the PCK inhibitor 3-MPA and the CPT1A inhibitor ETO was administered by intraperitoneal injection, and the results showed a strong synergistic anti-tumor effect specifically in the PET-negative PDX (Fig. 6F–I). These findings suggest that targeting the PCK/CPT1A axis may represent a promising therapeutic strategy for 18F-FDG-PET/CT-negative gastric cancer.

## Discussion

Based on tumor metabolic feature, the 18F-FDG-PET/CT technology has been developed and widely used in clinical diagnostics. However, a considerable proportion of gastric cancer cases are negative on 18F-FDG-PET/CT, indicating that they undergo metabolic reprogramming beyond aerobic glycolysis. In the present study, we observed that 18F-FDG-PET/CT-negative gastric cancers exhibit decreased glucose uptake and glycolysis; instead, these cancers utilize glutamine-based gluconeogenesis and fatty acid oxidation to fulfill their metabolic demands for macromolecule biosynthesis and energy supply. Targeted inhibition of the PCK/CPT, which includes key enzymes in gluconeogenesis and fatty acid oxidation, significantly suppresses the growth of 18F-FDG-PET/CT-negative gastric tumors.

In most cancers, upregulated glycolysis directly enhances glycolytic shunts, such as the PPP and the serine synthesis pathway. These pathways support macromolecule synthesis by producing metabolic intermediates, including NADPH, R5P, and one-carbon units [2, 5–7]. NADPH is essential not only for the generation of reduced glutathione, a well-known antioxidant that neutralizes reactive oxygen species, but also for lipid and nucleotide synthesis [2, 5–7]. Both R5P and one-carbon units are critical building blocks for nucleotide biogenesis, which is essential for DNA synthesis and, consequently, cell proliferation [5, 6]. Gluconeogenesis, the reverse reaction of glycolysis, typically has an antagonistic effect on glycolysis and is often suppressed in most tumor cells. In the present study, we demonstrate that under conditions of limited glucose uptake, 18F-FDG-PET/CT-negative gastric cancer cells compensate for diminished glycolytic flux by activating gluconeogenesis. Mechanistically, PPARγ is highly expressed in these cells and transcriptionally upregulates PCK and CPT1A, the key metabolic enzymes that drive the metabolic reprogramming characteristic of FDG-PET/CT-negative gastric cancers. These findings align with recent reports showing elevated expression of gluconeogenic enzymes in certain tumor types, such as lung cancer [22]. Under glucose-restricted conditions, activation of gluconeogenesis helps sustain the biosynthesis of macromolecules by compensating for reduced glycolytic output [23, 35, 36]. Collectively, our work together with emerging evidence suggests that in tumors with low aerobic glycolytic activity, targeting gluconeogenesis may represent a promising therapeutic strategy.

Glutamine addiction is a hallmark of several types of tumors, including glioblastoma, colorectal, pancreatic, lung, and prostate cancers [37–41]. Once transported into cells, glutamine plays a pleiotropic role in cancer development [42]. For instance, glutamine can serve as a nitrogen donor for purine and pyrimidine synthesis, be exchanged for other amino acids, or fuel the tricarboxylic acid (TCA) cycle, thereby compensating for oxidative phosphorylation and ATP generation [43–45]. More importantly, in glucose-deficient conditions or in cancer cells that are insensitive to the glycolysis inhibitor 2-DG, glutamine can back up glycolytic intermediates through gluconeogenesis. These intermediates are used to synthesize nucleic acids or proteins required for rapid cell proliferation [43–45]. In this study, we demonstrate that glutamine represents the most abundant amino acid in 18F-FDG-PET/CT-negative gastric cancer. Glutamine enters gluconeogenesis through the TCA cycle and replenishes glycolytic intermediates, thereby supplying precursors for macromolecule biosynthesis. Mechanistically, the elevated expression of the key enzymes PCK1/2 in these cancer cells supports a glutamine-driven gluconeogenic pathway. This reveals an alternative, glutamine-dependent mode for macromolecule biosynthesis in 18F-FDG-PET/CT-negative gastric cancer.

It is important to note that gluconeogenesis is an energy-consuming process [22, 23, 36]. This raises the question of how 18F-FDG-PET/CT-negative gastric cancer cells meet their energy demands. Typically, cancer cells rely on glycolysis to support macromolecule biosynthesis and utilize glutamine anaplerosis to fuel the TCA cycle and energy production. However, our findings indicate that 18F-FDG-PET/CT-negative gastric cancer cells have a significantly higher dependence on mitochondrial oxidative metabolism from fatty acid oxidation compared to glutamine or glucose metabolism. Compared to glucose and glutamine, a single molecule of fatty acid can generate more ATP, making fatty acid oxidation a more efficient pathway for energy production. The elevated expression of the key enzyme CPT1A supports fatty acid oxidation in 18F-FDG-PET/CT-negative gastric cancer cells. This distinctive metabolic cooperation, in which glutamine-driven gluconeogenesis operates synergistically with activated fatty acid oxidation, represents a previously unrecognized dual metabolic adaptation in this 18F-FDG-PET/CT-negative cancer.

In summary, the present study elucidated the roles of glutamine-based gluconeogenesis and fatty acid oxidation in 18F-FDG-PET/CT-negative gastric cancer. It identified an alternative metabolic reprogramming mode under conditions of low glucose uptake, thereby expanding our understanding of cancer metabolism. Furthermore, targeting the PCK/CPT axis may represent a potential therapeutic strategy for 18F-FDG-PET/CT-negative gastric cancer.

## Materials and methods

### Cell lines and tissues

Human GES, KATO-III, MKN-45, and MKN-1 cell lines were cultured in RPMI 1640 medium (Sigma-Aldrich), supplemented with 10% fetal bovine serum (FBS; Gibco) and 1% penicillin/streptomycin (Thermo Fisher Scientific). Human A549 and AGS cell lines were cultured in DMEM/F12 medium (Sigma-Aldrich), supplemented with 10% FBS and 1% penicillin/streptomycin. Human DU145 and HeLa cell lines were cultured in DMEM medium (Sigma-Aldrich), supplemented with 10% FBS and 1% penicillin/streptomycin. All cell lines were routinely tested negative for mycoplasma contamination. Cell line authentication was performed using Short Tandem Repeat (STR) profiling within the past three years. Human specimens were obtained from the Department of Biobank, Division of Clinical Research, The First Hospital of Jilin University.

### Cell fractionations

Cells were lysed in cold WB-IP lysis buffer (Beyotime) for 30 min and then centrifuged at 4 °C for 5 min at 14,000 × *g* to remove cell debris. The supernatant was collected and used as whole-cell lysates. Whole-cell lysates were mixed with 2× loading buffer and boiled at 100 °C for 5 min, followed by Western blotting (WB). For WB, protein samples were resolved by SDS-PAGE and transferred to PVDF membranes (Millipore). Membranes were blocked with 5% (w/v) nonfat dried milk in TBS (pH 7.4) containing 0.1% Tween 20 (TBST) and incubated with the appropriate primary antibodies in 5% (w/v) BSA in TBST overnight at 4 °C. Mouse- or rabbit-conjugated HRP secondary antibodies were added to the membranes for 50 min at room temperature, followed by washing three times with 1× TBST. The proteins were visualized using Chemiluminescent HRP Substrate (Millipore) on a Chemiluminescence Image System (Tanon Science and Technology Co., Ltd.).

### Antibodies used in this study

Primary antibodies included: anti-GLUT1 (Proteintech, #21829-1-AP), anti-HK2 (Cell Signaling Technology, #2106), anti-PFKP (Cell Signaling Technology, #5412), anti-ALDOA (Cell Signaling Technology, #3188), anti-TPI (Cell Signaling Technology, #34088), anti-GAPDH (Cell Signaling Technology, #2118), anti-PGK1 (Cell Signaling Technology, #68540), anti-ENO (Cell Signaling Technology, #3810), anti-PKM2 (Cell Signaling Technology, #3198), anti-ASCT2 (ABclonal, #A20485), anti-GLS (ABclonal, #A20554), anti-FH (ABclonal, #A20451), anti-MDH1 (ABclonal, #A20885), anti-MDH2 (ABclonal, #A20674), anti-PCK1 (ABclonal, #A2036), anti-PCK2 (Proteintech, #14892-1-AP), anti-FBP1 (ABclonal, #A20564), anti-CPT1A (ABclonal, #A20193), anti-ACADVL (ABclonal, #A20187), anti-ACADM (ABclonal, #A20365), anti-ACADS (ABclonal, #A20458), anti-ECHS1 (ABclonal, #A20967), anti-HADH (ABclonal, #A20879), anti-GLUT1 (Proteintech, #21829-1-AP), anti-PGC1α (ABclonal, #A20995), anti-CREB1 (ABclonal, #A11989), anti-FOXO1 (ABclonal, #A2934), anti-PPARγ (Cell Signaling Technology, #2435S) and anti-β-Actin (Proteintech, #23660-1-AP). Secondary antibodies included: goat anti-mouse IgG secondary antibody HRP conjugated (SAB, #L3032) and goat anti-rabbit IgG secondary antibody HRP conjugated (SAB, #L3012).

### Quantification assay of metabolites

Glucose uptake was measured using the Glucose Uptake Fluorometric Assay Kit (BioVision) according to the manufacturer’s instructions. Levels of ATP and G6P were detected using the ATP Assay Kit (Beyotime) and G6P Assay Kit (Beyotime), respectively, in accordance with the manufacturer’s instructions.

### Chromatin immunoprecipitation (ChIP) assay

Cells were cross-linked with 1% formaldehyde for 10 min at room temperature, followed by incubation with glycine for 5 min to quench the reaction. After washing with ice-cold PBS, nuclei were isolated using the Chromatin IP Kit (#9003, Cell Signaling Technology) according to the manufacturer’s protocol. Chromatin was sheared using a Bioruptor (UCD-300, Diagenode) set to high mode, and the samples were centrifuged at 9400 × *g* for 10 min at 4 °C. The supernatant was subjected to immunoprecipitation overnight with an anti-PPARγ antibody, using an isotype IgG as a negative control. ChIP-grade Protein G magnetic beads were then added and incubated with rotation for 2 h at 4 °C. Following washing steps, bound complexes were eluted with ChIP Elution Buffer. Cross-links were reversed by heating at 65 °C, and proteins were digested with proteinase K for 2 h. Finally, DNA was purified, eluted in distilled water, and analyzed by RT-qPCR.

### Quantitative real-time PCR

Total RNA was isolated using Trizol reagent, and single-strand cDNA was synthesized from the RNA using a High-Capacity cDNA Reverse Transcription Kit (TransGen Biotech) according to the manufacturer’s instructions. Quantitative real-time PCR (qPCR) analysis was performed using the SYBR Real-Time PCR Premix (Takara) on a Roche LightCycler 480 sequence detection system. The thermal cycling conditions were as follows: initial denaturation at 95 °C for 5 min, followed by 40 cycles of amplification (95 °C for 30 s, 55 °C for 40 s, and 72 °C for 1 min). The primer sequences were shown in Supplementary table 1.

### Quantification assay of amino acids

A total of 3 × 10^5^ cells were cultured in DMEM medium containing 10% fetal bovine serum for 24 h. Subsequently, 100 µL of supernatant was collected from the culture medium (experimental group), and an additional 100 µL of DMEM medium containing 10% fetal bovine serum was prepared as the control group. To each sample, 500 µL of acetonitrile was added to the supernatant of both the experimental and control groups. The mixtures were shaken and vortexed thoroughly, followed by centrifugation at 14,000 rpm for 5 min at 4 °C. The supernatant was then collected and subjected to LC-MS analysis. Agilent eclipse plus C18 (2.1 × 150 mm, 3.5 μm, Agilent, USA); column temperature: 4 °C; Injection volume: 20 µL; The mobile phase in the experiment was: mobile phase a, water + 0.1% formic acid; Mobile phase B, acetonitrile + 0.1% formic acid. A triple toftm 5600 high-resolution mass spectrometers equipped with electrospray ionization (ESI) mode was used to scan in positive ion time-of-flight mass spectrometry (TOF scan) mode with an optimized decluster voltage (DP) of 100 V and an injection voltage (CE) of 10 EV in positive ion mode. The mass spectrometry scanning parameters were set as follows: curtain gas: 30 psi; Atomization gas: 50 psi; heating gas: 50 psi; ion spray voltage: 5500 V; source temperature: 500 °C. The raw data obtained by high-performance liquid chromatography time of flight tandem mass spectrometry (HPLC-TOFMS) were collected by the data acquisition software in the analyst 1.5.1 software package, and the data were further denoised and normalized by MaekerView software.

### ^13^C-tracing assessment

5 × 10^6^ cells were cultured in DMEM medium containing 10% fetal bovine serum and 4 mM [^13^C_5_]-Glutamine for 24 h. Quickly pour out the medium, add PBS after ice bath and wash it again, use a pipette to suck up the residual PBS, mix methanol with double distilled water at a volume of 4:1, and freeze it in a −80 °C refrigerator for 5 h. Add 500 µL of the above solution to the Petri dish, and place the Petri dish at −80 °C for 20 min. Place the Petri dish on dry ice and scrape off the cells with a cell knife. The cells and methanol water suspension were collected and stored in 1.5 mL EP tubes with a pipette gun and stored at −80 °C; Take another 500 µL of methanol water mixture at −80 °C, rinse the Petri dish once, collect the rinsing solution in 1.5 mL EP tube, and store it at −80 °C. The cells and methanol water suspension were incubated in a centrifuge at 1500 rpm for 30 min at 4 °C; Centrifuge the sample with a high-speed centrifuge to take the supernatant, and set the working conditions: 12,000 rpm, 4 °C, centrifugation for 10 min; Take the supernatant and put it in a 1.5 mL EP tube; The supernatant was dried by centrifugal concentrator, and the sample powder was prepared by removing water; Redissolve the powder with 100 µL of 1% acetonitrile, and take the supernatant to be tested (LC-MS). Acquity UPLC HSS T3 1.8 μm column was used for reverse phase chromatographic analysis; Acquity UPLC BEH amide 1.7 μm column was used for normal phase chromatographic analysis (2.1 × 100 mm columns, Waters, Dublin,Ireland). The instrument used an ultra-high-pressure liquid chromatograph (Agilent 1290 II, Agilent Technologies, Germany) in series with a high-resolution mass spectrometer (5600 triple TOF plus, AB Sciex, Singapore). All analyses were performed in electrospray ionization (esi-) mode with the following conditions: air curtain gas = 35, negative ion mode particle spray voltage = 4500 V, source temperature = 450 °C, ion source gas 1 = 50, ion source gas 2 = 50; Data acquisition: time of flight mass spectrometry (TOF) full scan was used for primary mass spectrometry; The secondary mass spectrum information dependent acquisition (IDA), collision energy (-) 30 ± 15 EV.

### EdU Click-iT assay

Cells were seeded onto glass coverslips and cultured for 24 h. Subsequently, the cells were treated with EdU (40 µM) for 20 min. After treatment, the cells were washed and fixed in 4% paraformaldehyde for 7 min. Following fixation, EdU staining was performed using the Click-iT™ Cell Reaction Buffer Kit (Invitrogen, C10269) and Alexa Fluor™ 647 Azide (Invitrogen, A10277) according to the manufacturer’s instructions. Cell nuclei were washed three times with PBS and then stained with DAPI (Sigma, D9542). Fluorescent images were acquired using a confocal microscope (Carl Zeiss LSM880) and analyzed with NIS-Elements AR software version 5.01 (Nikon). Statistical analysis was performed using Prism 8 (GraphPad Software).

### Extracellular flux analysis

ECAR and OCR were measured using an XF24 Extracellular Flux Analyzer (Seahorse, Agilent). Briefly, cells from each group were seeded in an XF culture dish at a density of 1 × 10^4^ cells per well one day prior to the test.

### Micro-PET/CT analysis

Gastric cancer cell suspensions (200 µL, 1 × 10^6^ cells) were inoculated into the left forelimb armpit of nude mice. Micro-PET/CT imaging was performed when a tumor mass approximately 1 cm in size was observed at the injection site. Mice were fasted for 12 h prior to the examination, with water provided ad libitum. The ambient temperature was maintained at 25 °C. Mice were anesthetized using 3% isoflurane via inhalation, with an oxygen flow rate of 2 L/min in the anesthesia chamber. After successful anesthesia induction, 200 µL of the 18F-FDG imaging agent was administered via tail vein injection. After 40 min, the mice were placed in a Siemens Inveon micro-PET/CT scanner for imaging. The imaging procedure began with a 5-min CT scan using the device’s CT module, with a field of view of 8.5 cm × 5.7 cm. The CT image data were used to locate and delineate the tumor area and to correct for PET image attenuation. Following CT scanning, PET scanning and image acquisition were initiated. Image processing and data acquisition were performed by delineating the tumor area and other regions of interest (ROIs) under the guidance of CT images. The ROIs were analyzed using Inveon Research Workplace software to calculate the standardized uptake values (SUVs) of the tracer in the tumor site, liver, muscle, and brain.

### Single-cell suspension preparation

Single-cell suspensions were prepared from fresh human gastric cancer tissues. Briefly, collected tissues were minced into ~1 mm^3^ fragments and enzymatically digested using a Tumor Tissue Dissociation Solution (MCE, HY-K6011) according to the manufacturer’s protocol. The resulting digest was filtered through a 100-μm strainer, and the cells were washed, centrifuged, and resuspended. Final cell viability, confirmed by Trypan Blue exclusion to be >90%, was assessed prior to subsequent experiments.

### Flow cytometric cell sorting

Cells were resuspended in cold staining buffer (PBS with 2% FBS) at a density of 1 × 10^7^ cells/mL and incubated with a fluorochrome-conjugated anti-GLUT1 antibody for 30 min at 4 °C in the dark, using an isotype-matched antibody to define negative gating. Following two washes to remove unbound antibodies, the stained cells were subjected to sorting on a high-speed flow cytometer (BD FACSAria™ II). Distinct GLUT1^high^ and GLUT1^low^ populations, defined as the top 23.4% and bottom 39.8% of fluorescence intensity respectively, were sorted and collected into tubes containing DMEM with 10% FBS.

### Cell proliferation

For the cell proliferation assay, 1 × 10^5^ cells were seeded into culture vessels and incubated at 37 °C in a humidified incubator with 5% CO_2_. Cell counts were performed every 24 h over a 6-day period to generate the proliferation curve. Cell viability was assessed using the Cell Counting Kit-8 (CCK8; Beyotime) according to the manufacturer’s instructions. The absorbance was measured at 450 nm using a microplate reader.

### Xenograft analysis

For the xenograft assay, 1 × 10^7^ cells were resuspended in a mixture containing 50 µL of Matrigel (Corning) and 100 µL of PBS. The cell suspensions were then injected into the right flank of nude mice (8-week-old female). Tumor volumes were measured every 2 days. On day 22 after injection, the tumors were excised and analyzed. Tumor volumes were calculated based on the formula *V* = ab^2^/2, where a represents the length and b represents the width of the tumor. For Patient-Derived Xenograft (PDX) models of gastric cancer, the fresh tumor tissue was minced into small fragments (approximately 2–3 mm^3^) in a sterile dish. A single fragment was mixed 1:1 with Matrigel (Corning) and surgically implanted into the subcutaneous space on the left flank of an anesthetized 8-week-old female NCG (NOD/ShiLtJGpt-Prkdc^em26Cd52^Il2rg^em26Cd22^/Gpt) mouse (GemPharmatech Co.,Ltd). Upon day 14 post-tumor inoculation, the mice were intraperitoneally injected with PBS (10 mg/kg), 3-MPA (5 mg/kg), ETO (10 mg/kg), or a combination of MPA and ETO every other day. After 14 days, the xenograft tumors were excised and analyzed. Experiments were approved by the Ethics Committee of Northeast Normal University (No. 202502004) (Changchun, China), and strictly complied with the ethical requirements.

### Immunohistochemistry analysis

Mouse tumor tissues were fixed and prepared for immunohistochemistry (IHC) as previously described. Briefly, paraffin-embedded sections of xenograft tumors were prepared and stained with an antibody against Ki-67. To quantify the IHC results, five random areas of each tissue section were examined, analyzed, and imaged using a Leica DMi8 microscope. 18F-FDG-PET/CT-negative and positive gastric cancer tissues from patients were obtained from the Biobank of China–Japan Union Hospital of Jilin University during surgery and stored at −80 °C. The human gastric cancer tissue microarray (#D198020) used in this study was purchased from Zhongke Guanghua (Xi’an) Intelligent Biotechnology Co., Ltd. The present study was approved by the Ethics Committee of Science and Technology of Northeast Normal University and conducted with the informed consent of all patients.

### Quantification and statistical analysis

All experiments were conducted at least three times independently. The sample size for each experiment is provided in the corresponding figure legend. Statistical analysis was performed using GraphPad Prism version 9.0 (GraphPad Software). Data are presented as mean ± standard deviation (SD). For comparisons between two groups, Student’s *t* test was used. When comparisons involved more than two groups, one-way or two-way analysis of variance (ANOVA) was performed, as indicated in the figures. All statistical tests were two-sided, and no adjustments for multiple comparisons were made unless specifically stated. *P* values less than 0.05 were considered statistically significant. *P* values are indicated on the graphs. A.U., arbitrary units. For animal studies, sample sizes were chosen based on previous experience. Mice were randomly assigned to different treatment groups. Investigators were blinded to the group allocation during tumor volume measurement and subsequent data analysis. No animals or data points were excluded from the analysis.

## Supplementary information

Supplementary Figures and Figure Legends

Supplementary Table 1

Original Data

## Data Availability

Data generated in this study are available within the article and its supplementary information files. The raw data supporting the findings are available from the corresponding author upon reasonable request. Source data for the figures are provided with this paper.
