# Supplementary material for: 18F-FDG-PET/CT-negative gastric cancer employs glutamine-based gluconeogenesis and fatty acid oxidation to support tumor growth
Source: Cell Death Dis. 2026 Mar 26;17(1):365. doi: 10.1038/s41419-026-08662-9 (PMC13039690; doi:10.1038/s41419-026-08662-9)
Supplement: Supplementary file 1 — Supplementary Figures and Figure Legends [file 41419_2026_8662_MOESM1_ESM.pdf]

Figure S1

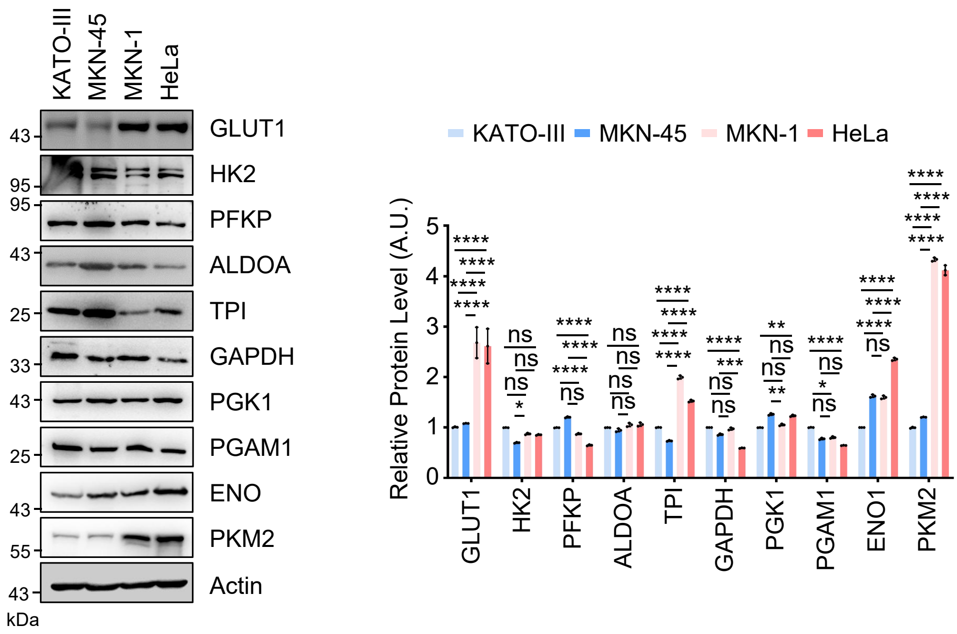

Figure S1. 18F-FDG-PET/CT-negative gastric cancer cell lines present glycolysis-independent metabolic pattern. Analysis of the expression of major metabolic enzymes in glycolysis. KATO-III, MKN-45, MKN-1 and HeLa cell lines were subjected to enzyme expression assays by WB. Representative images (left panel) and quantification of proteins (right panel) were displayed. Mean ± SD, n=3; One-way ANOVA: $^{*}P<0.05$; $^{**}P<0.01$; $^{***}P<0.001$; $^{****}P<0.0001$. ns, not significant. A.U., arbitrary units.

Figure S2

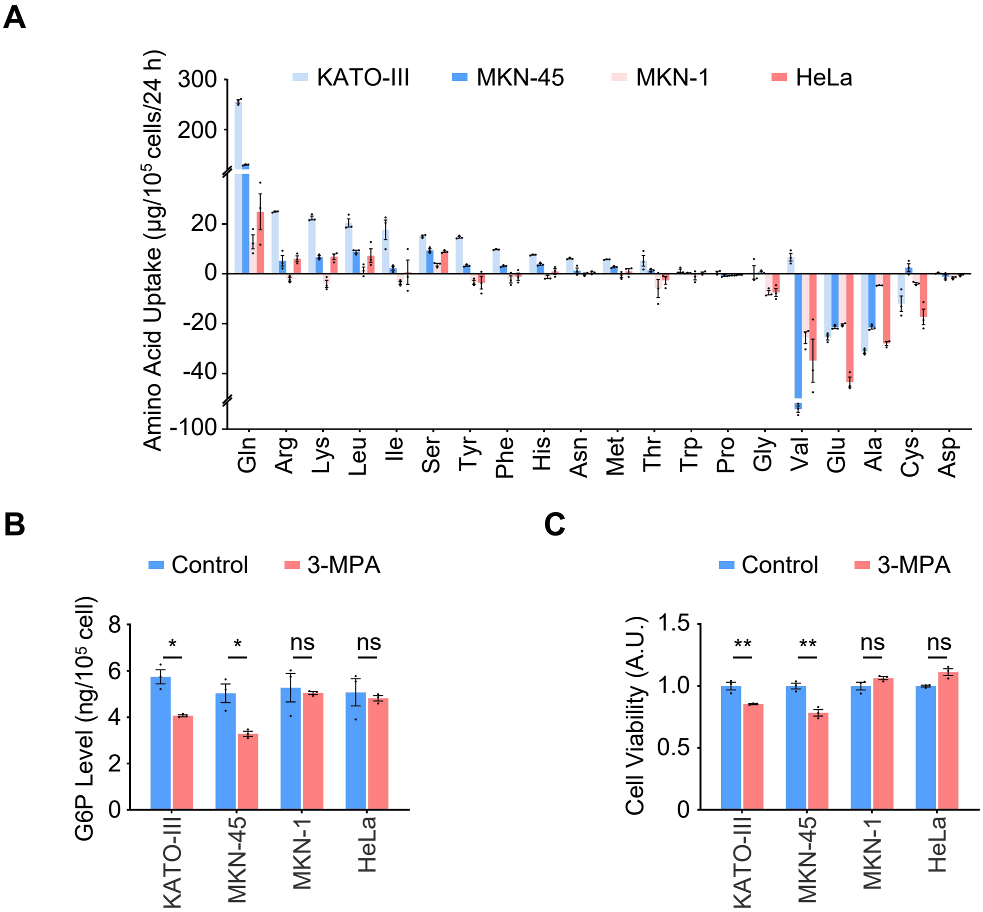

Figure S2. Glutamine-based gluconeogenesis supports macromolecule synthesis in 18F-FDG-PET/CT-negative gastric cancer. (A) Assay for Amino Acid Uptake. KATO-III, MKN-45, MKN-1, and HeLa cell lines were cultured for 24 hours, and the supernatants were analyzed for the content of 20 amino acids. Data are presented as mean ± SD (n=3). (B) Evaluation of G6P Levels. KATO-III, MKN-45, MKN-1, and HeLa cell lines were treated with or without the PCK1/2 inhibitor MPA for 48 hours. G6P levels were measured by ELISA. Data are presented as mean ± SD (n=3). Statistical analysis was performed using two-way ANOVA: $^{*}P$<0.05; ns, not significant. A.U., arbitrary units. (C) Analysis of Cell Viability. KATO-III, MKN-45, MKN-1, and HeLa cell lines were treated with or without the PCK1/2 inhibitor 3-MPA for 48 hours. Cell viability was assessed using the CCK8 Assay Kit. Data are presented as mean ± SD (n=3). Statistical analysis was performed using two-way ANOVA: $^{**}P$<0.01; ns, not significant.

Figure S3

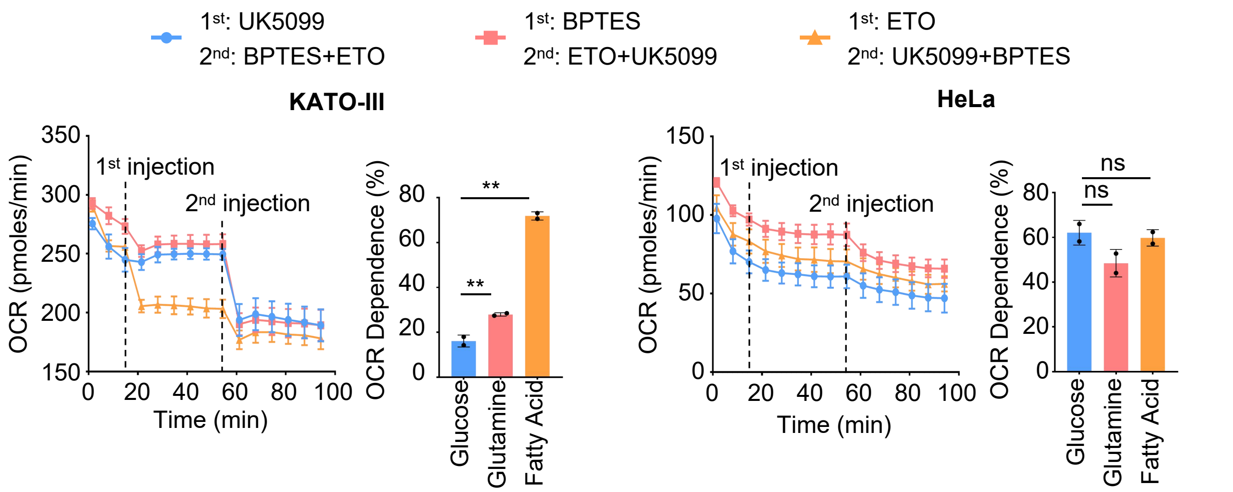

Figure S3. Evaluation of the contribution ratio of different nutrients to OCR. KATO-III and HeLa cell lines were treated with specific inhibitors of different metabolic pathways prior to OCR assessment. Real-time OCR under different conditions was plotted (left panel), and associated quantifications were shown (right panel). The inhibitors used were: UK5099 (30 μM, inhibitor of glucose metabolism), BPTES (40 μM, inhibitor of glutamine metabolism), and ETO (20 μM, inhibitor of fatty acid oxidation metabolism). Data are presented as mean ± SD (n=3). Statistical analysis was performed using one-way ANOVA: $^{**}P<0.01$; ns, not significant.

Figure S4

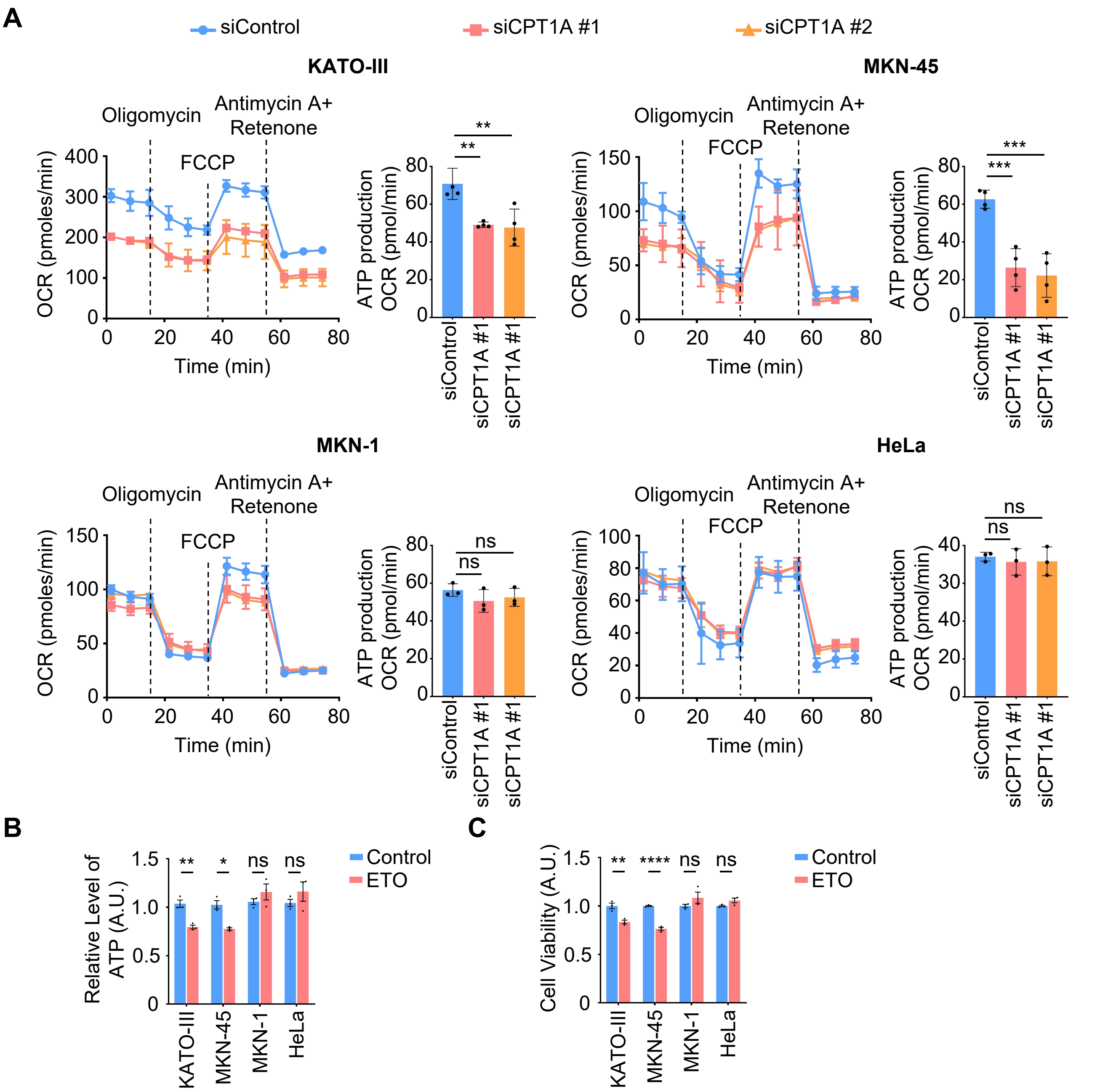

Figure S4. CPT1A is required for ATP production in 18F-FDG-PET/CT-negative gastric cancer cell lines. (A) Detection of oxygen consumption rate (OCR). KATO-III, MKN-45, MKN-1 and HeLa cell lines were seeded in a Seahorse XFp analyzer and treated with oligomycin, FCCP, and antimycin A/rotenone sequentially. A plot depicting real-time OCR under different conditions (left panel) and quantification of the rate of oxygen consumption for ATP production (right panel) were displayed. Mean ± SD, n=3. One-way ANOVA: ***P*<0.01; ****P*<0.001; ns, not significant. (B) Evaluation of ATP Levels. KATO-III, MKN-45, MKN-1, and HeLa cell lines were treated with or without CPT1A inhibitor ETO (20 μM) for 48 h. ATP levels were measured using the ATP Assay Kit. Data are presented as mean ± SD (n=3). Statistical analysis was performed using two-way ANOVA: **P*<0.05; ***P*<0.01; ns, not significant. (C) Analysis of Cell Viability. KATO-III, MKN-45, MKN-1, and HeLa cell lines were treated with or without the CPT1A inhibitor ETO for 48 h. Cell viability was assessed using the CCK8 Assay Kit. Data are presented as mean ± SD (n=3). Statistical analysis was performed using two-way ANOVA: ***P*<0.01; *****P*<0.0001; ns, not significant.

Figure S5

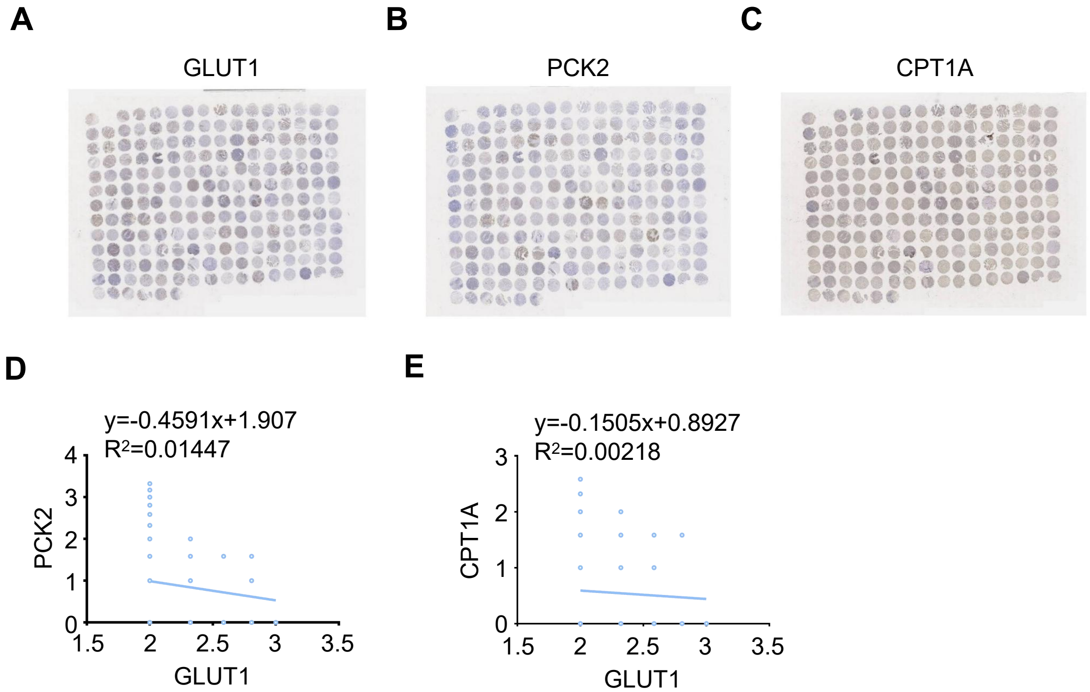

Figure S5. The expression of PCK2 or CPT1A negatively associate with GLUT1 in gastric cancer. (A-C) IHC Analysis of GLUT1, PCK2, and CPT1A in a Gastric Carcinoma Tissue Microarray. A tissue microarray containing 190 cases of gastric cancer was subjected to IHC analysis to evaluate the levels of GLUT1 (A), PCK2 (B), and CPT1A (C). (D and E) Correlation analysis of GLUT1 with PCK2 or CPT1A. Analysis of a public single-cell RNA-seq dataset of gastric cancer (GSE134520) revealed that PCK2 and CPT1A expression levels were higher in GLUT1^{low} tumor cells than in GLUT1^{high} cells.

Figure S6

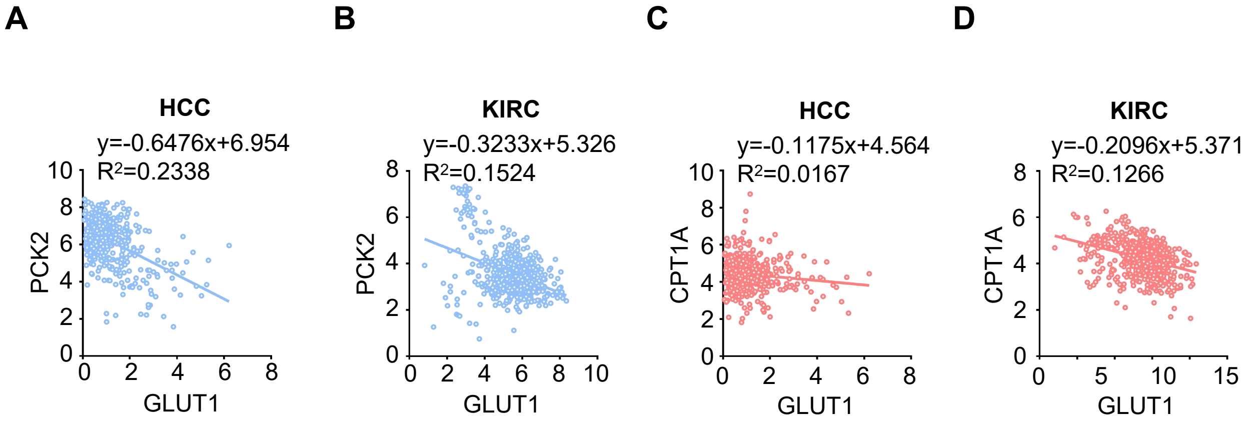

Figure S6. The expression of PCK2 or CPT1A negatively associate with GLUT1 expression in HCC and KIRC. Analysis of TCGA data to assess correlations between PCK2/CPT1A and GLUT1 expression in HCC and KIRC. HCC, Hepatocellular Carcinoma; KIRC, Kidney Renal Clear Cell Carcinoma.
