# Supplementary figures and images for: 18F-FDG-PET/CT-negative gastric cancer employs glutamine-based gluconeogenesis and fatty acid oxidation to support tumor growth
Source: Cell Death Dis. 2026 Mar 26;17(1):365. doi: 10.1038/s41419-026-08662-9 (PMC13039690; doi:10.1038/s41419-026-08662-9)

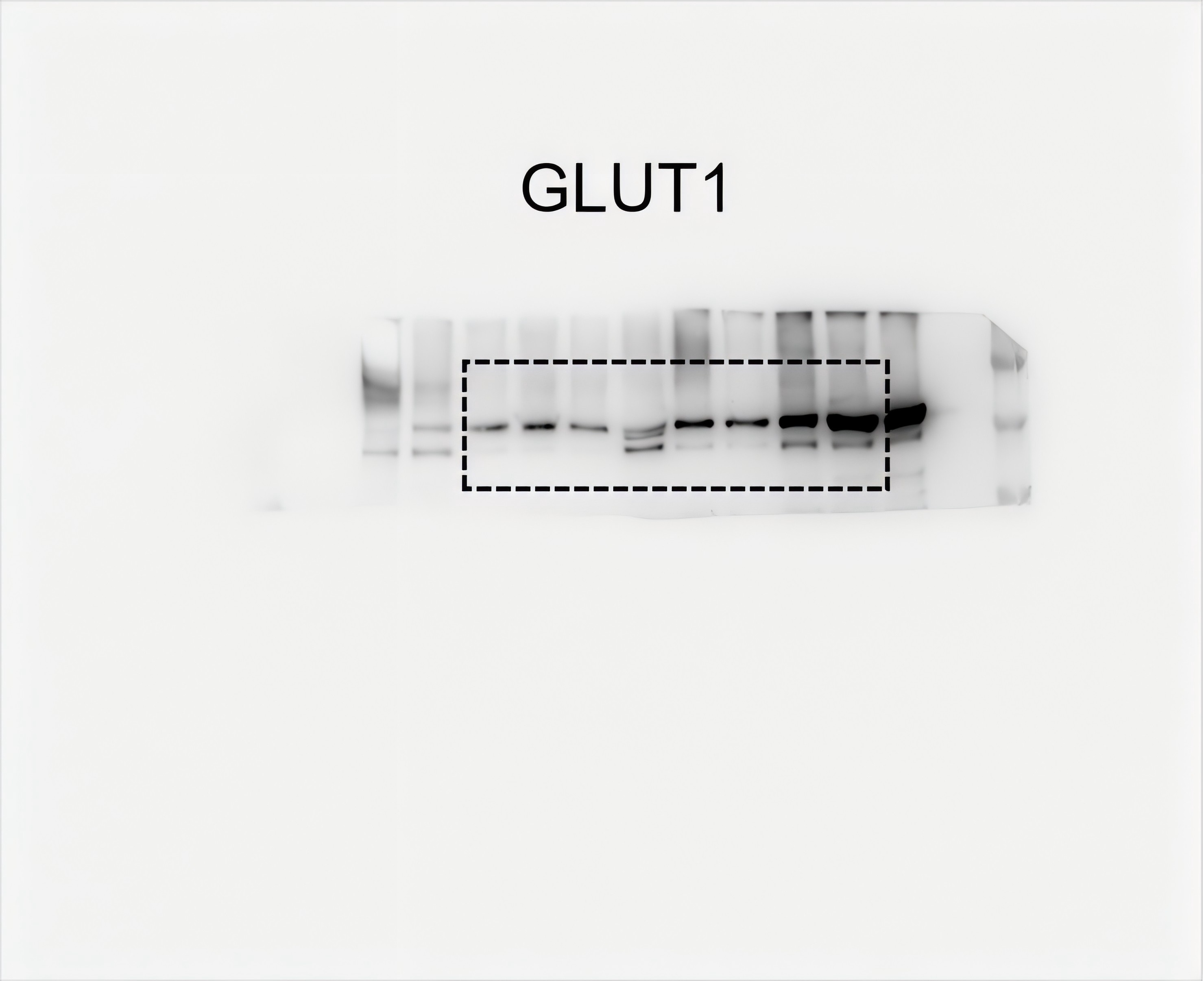

Supplement: Supplementary file 3 — Original Data [file 41419_2026_8662_MOESM3_ESM.zip › Original Data/Fig. 1C/1-GLUT1.tif]

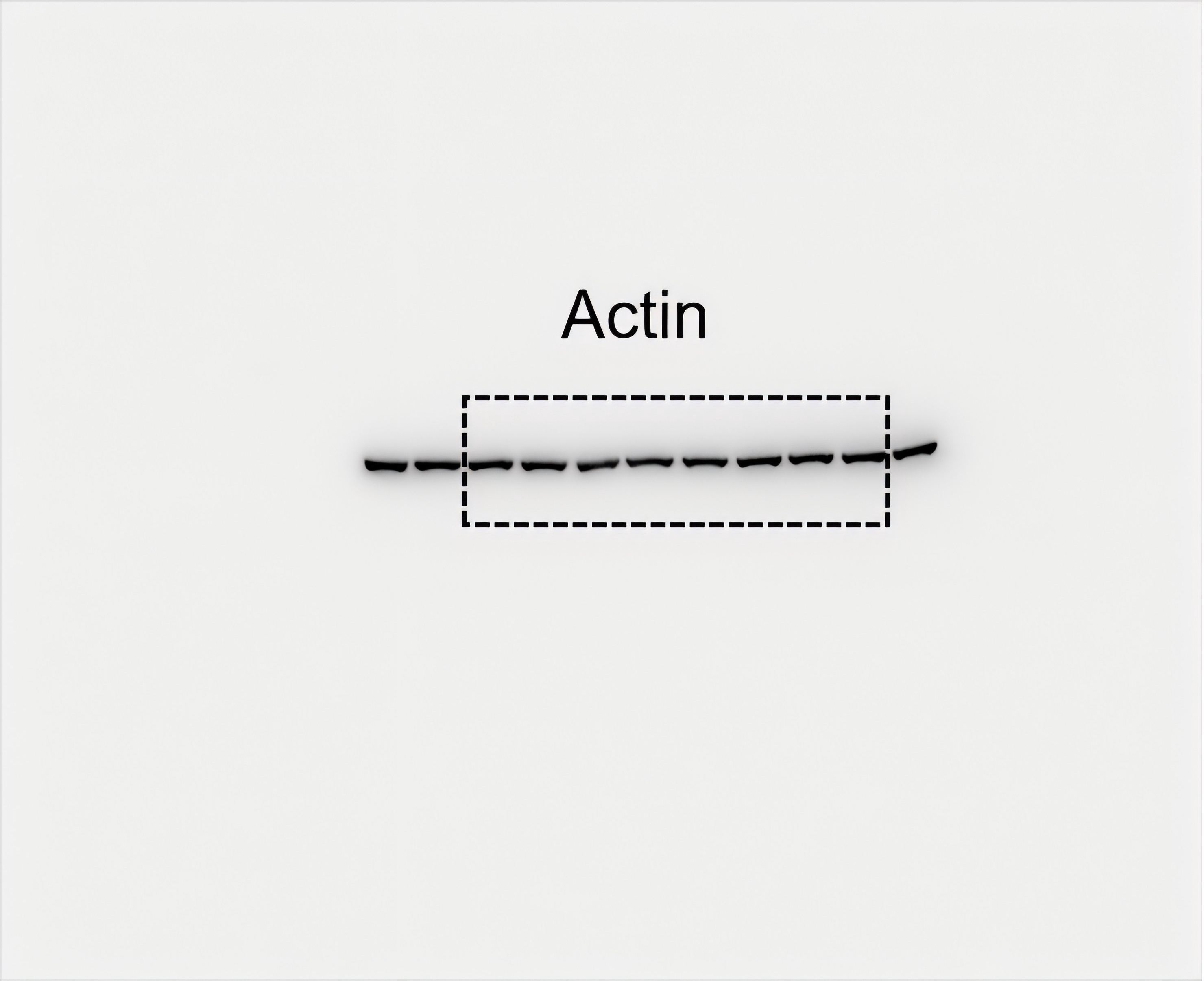

Supplement: Supplementary file 3 — Original Data [file 41419_2026_8662_MOESM3_ESM.zip › Original Data/Fig. 1C/2-Actin.tif]

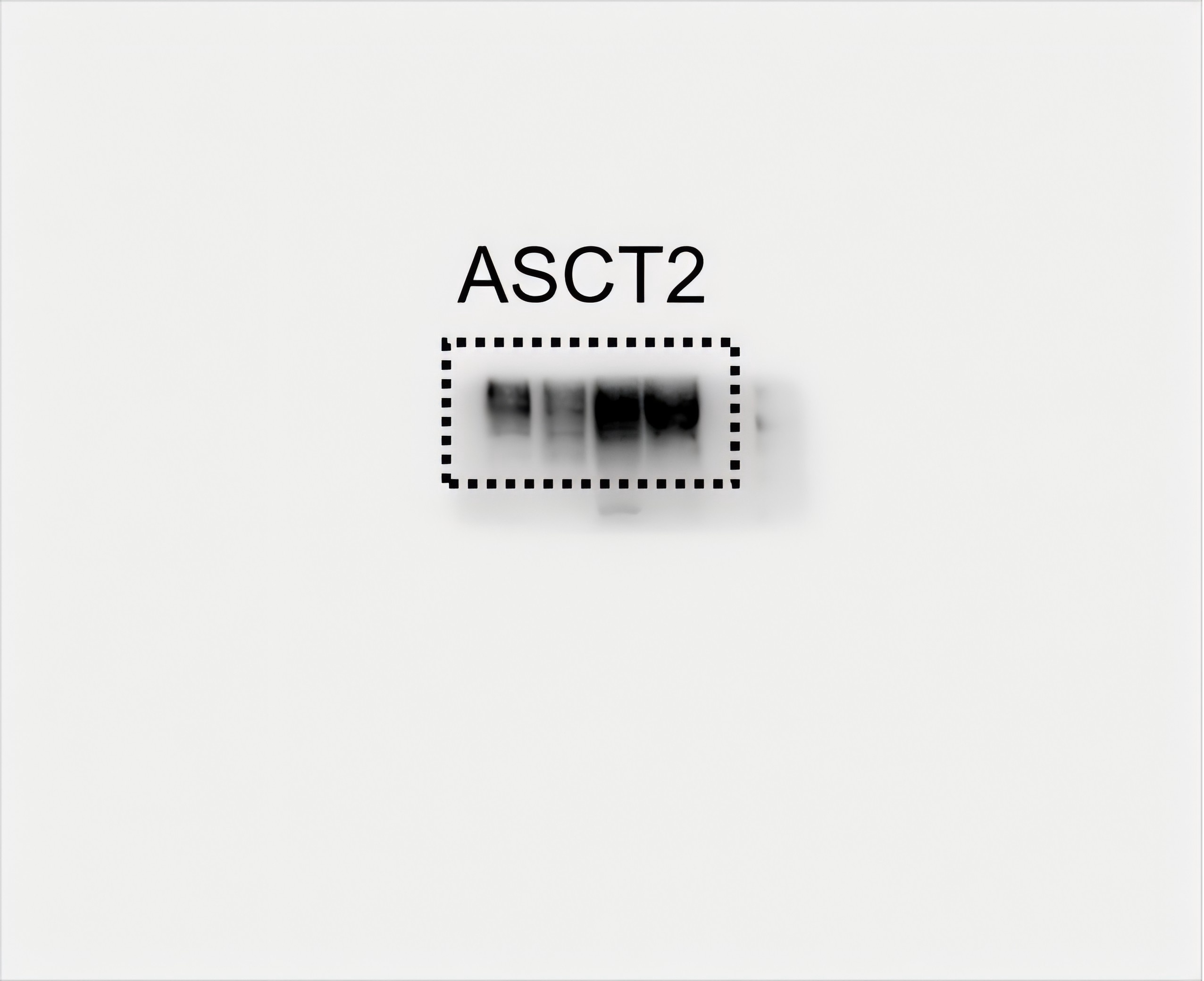

Supplement: Supplementary file 3 — Original Data [file 41419_2026_8662_MOESM3_ESM.zip › Original Data/Fig. 2D/1-ASCT2.tif]

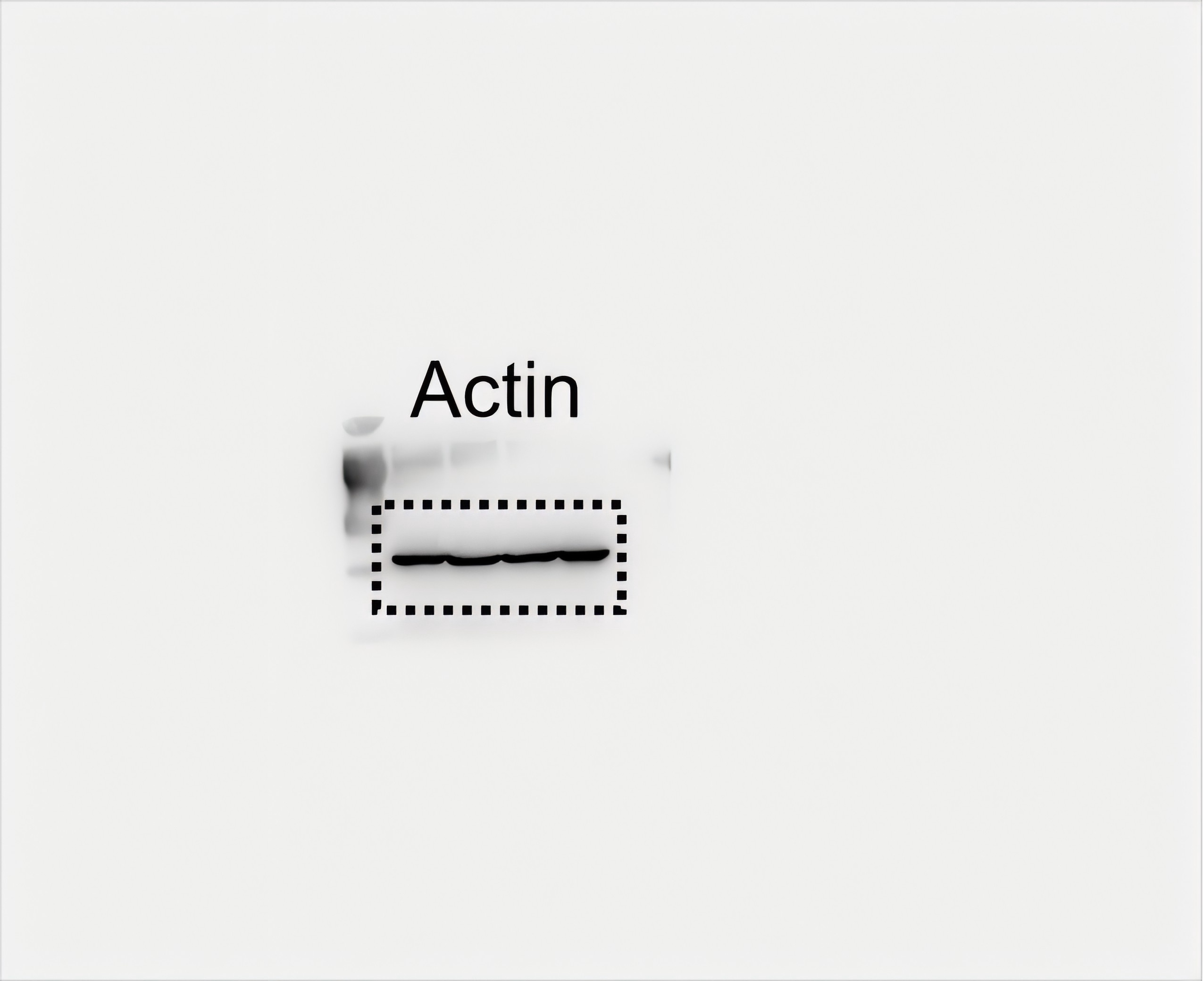

Supplement: Supplementary file 3 — Original Data [file 41419_2026_8662_MOESM3_ESM.zip › Original Data/Fig. 2D/10-Actin.tif]

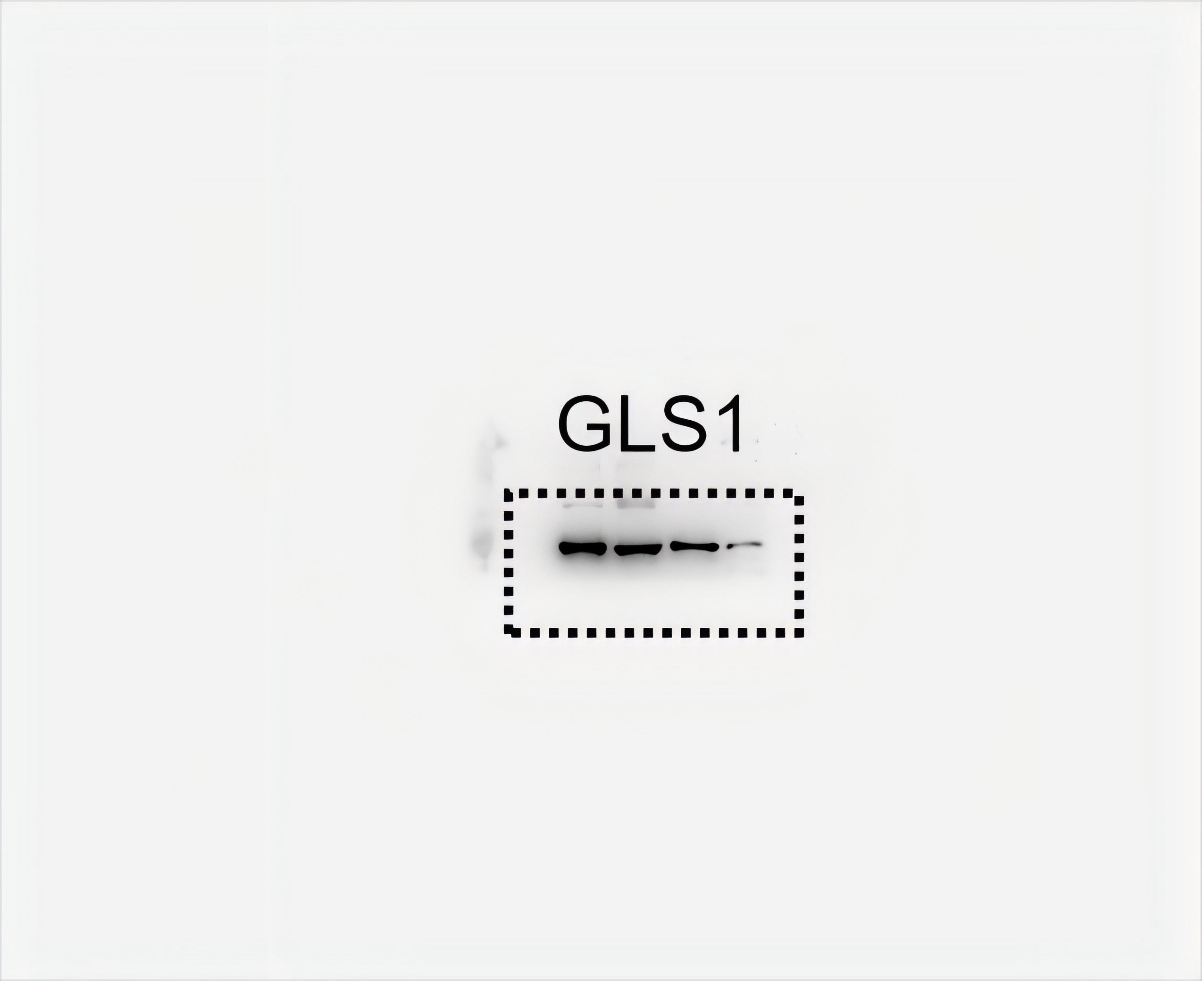

Supplement: Supplementary file 3 — Original Data [file 41419_2026_8662_MOESM3_ESM.zip › Original Data/Fig. 2D/2-GLS1.tif]

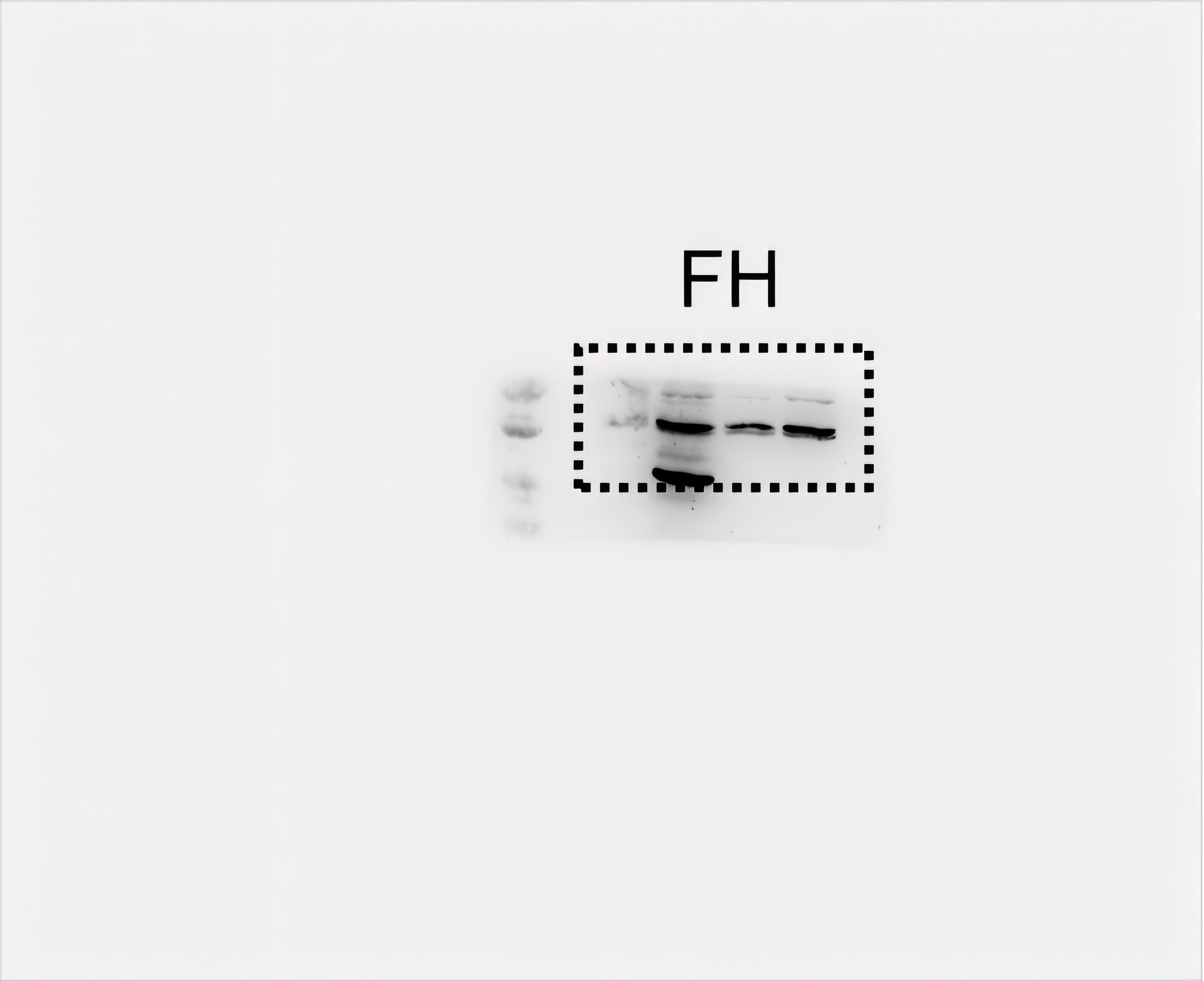

Supplement: Supplementary file 3 — Original Data [file 41419_2026_8662_MOESM3_ESM.zip › Original Data/Fig. 2D/3-FH.tif]

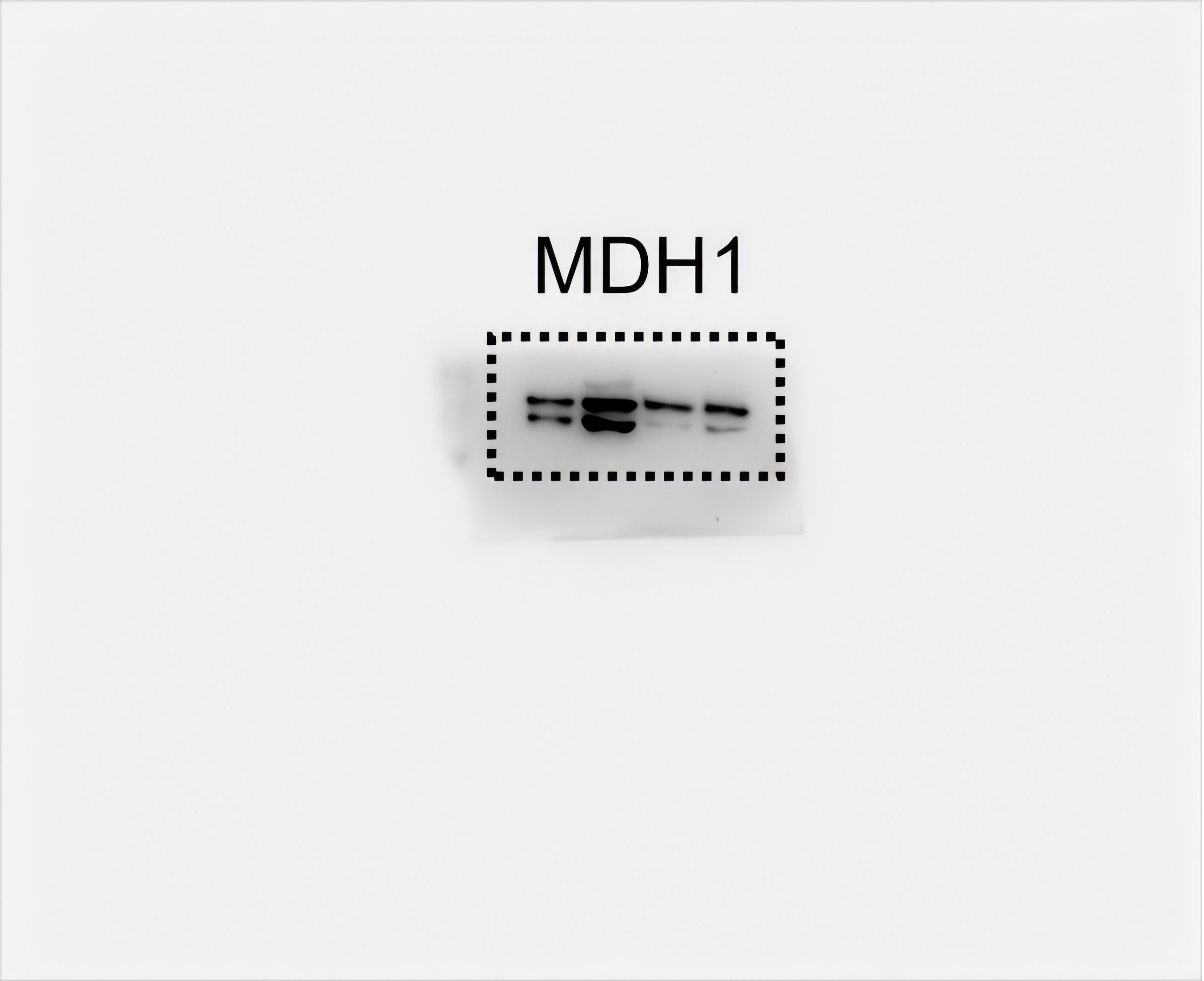

Supplement: Supplementary file 3 — Original Data [file 41419_2026_8662_MOESM3_ESM.zip › Original Data/Fig. 2D/4-MDH1.tif]

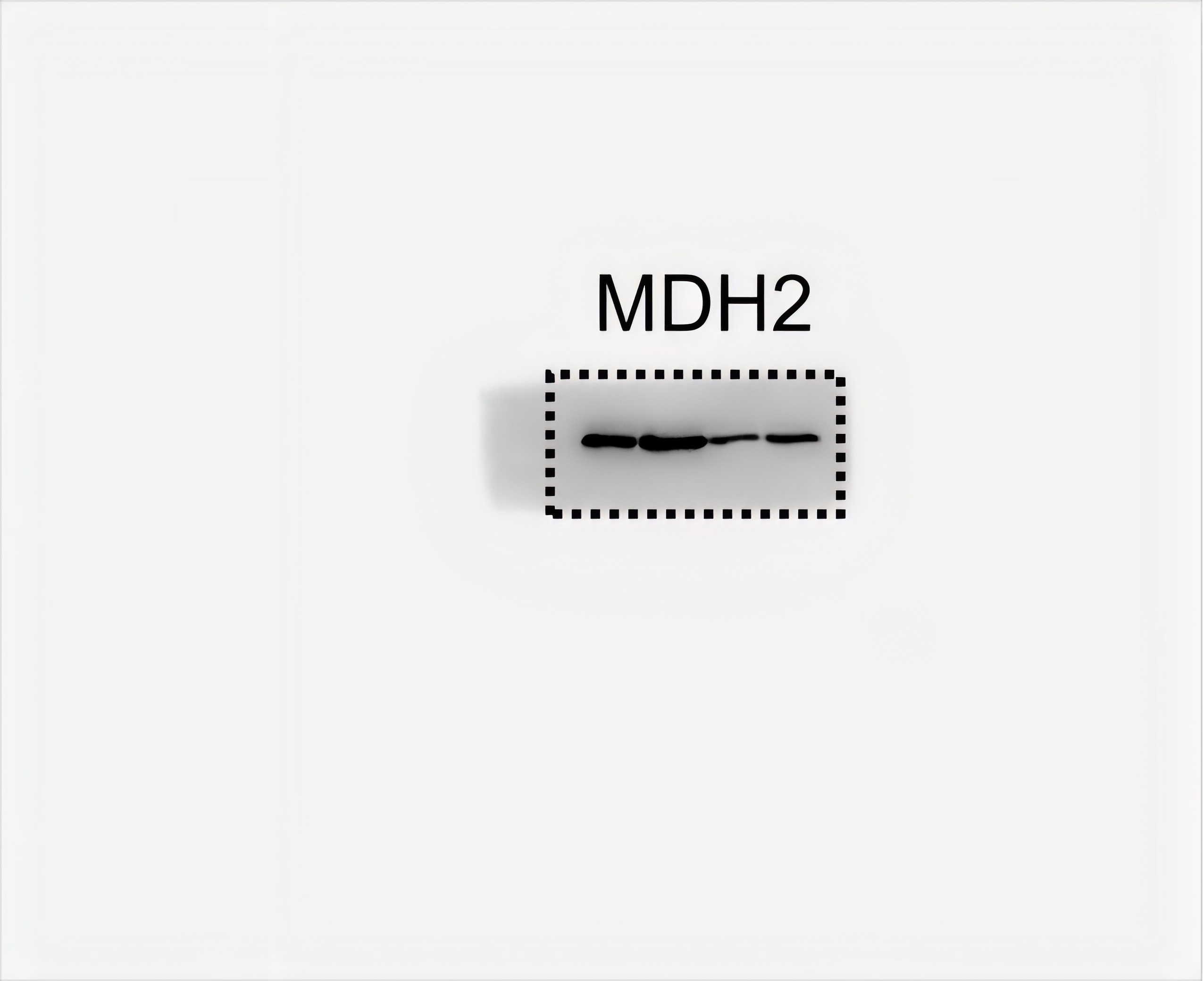

Supplement: Supplementary file 3 — Original Data [file 41419_2026_8662_MOESM3_ESM.zip › Original Data/Fig. 2D/5-MDH2.tif]

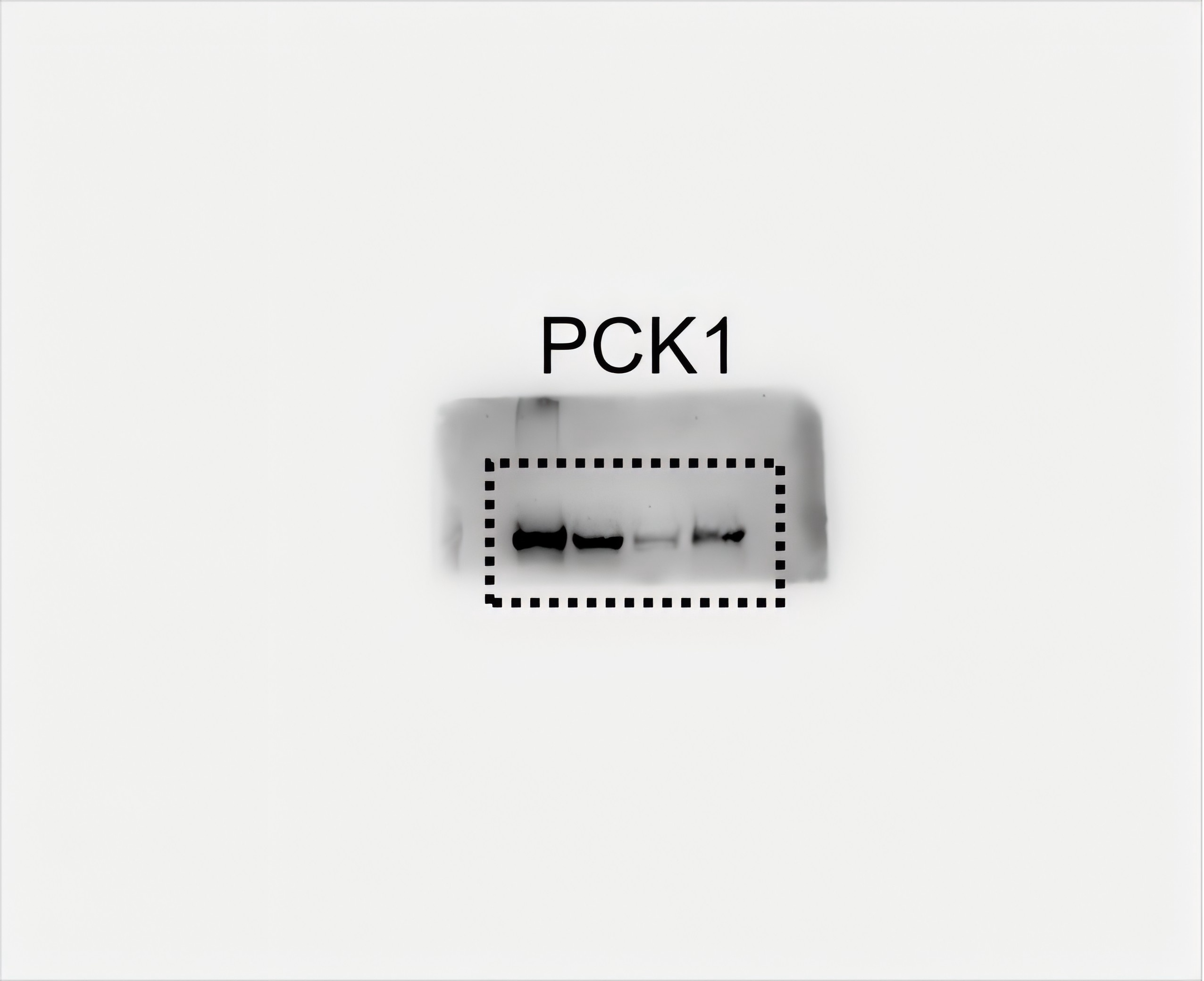

Supplement: Supplementary file 3 — Original Data [file 41419_2026_8662_MOESM3_ESM.zip › Original Data/Fig. 2D/6-PCK1.tif]

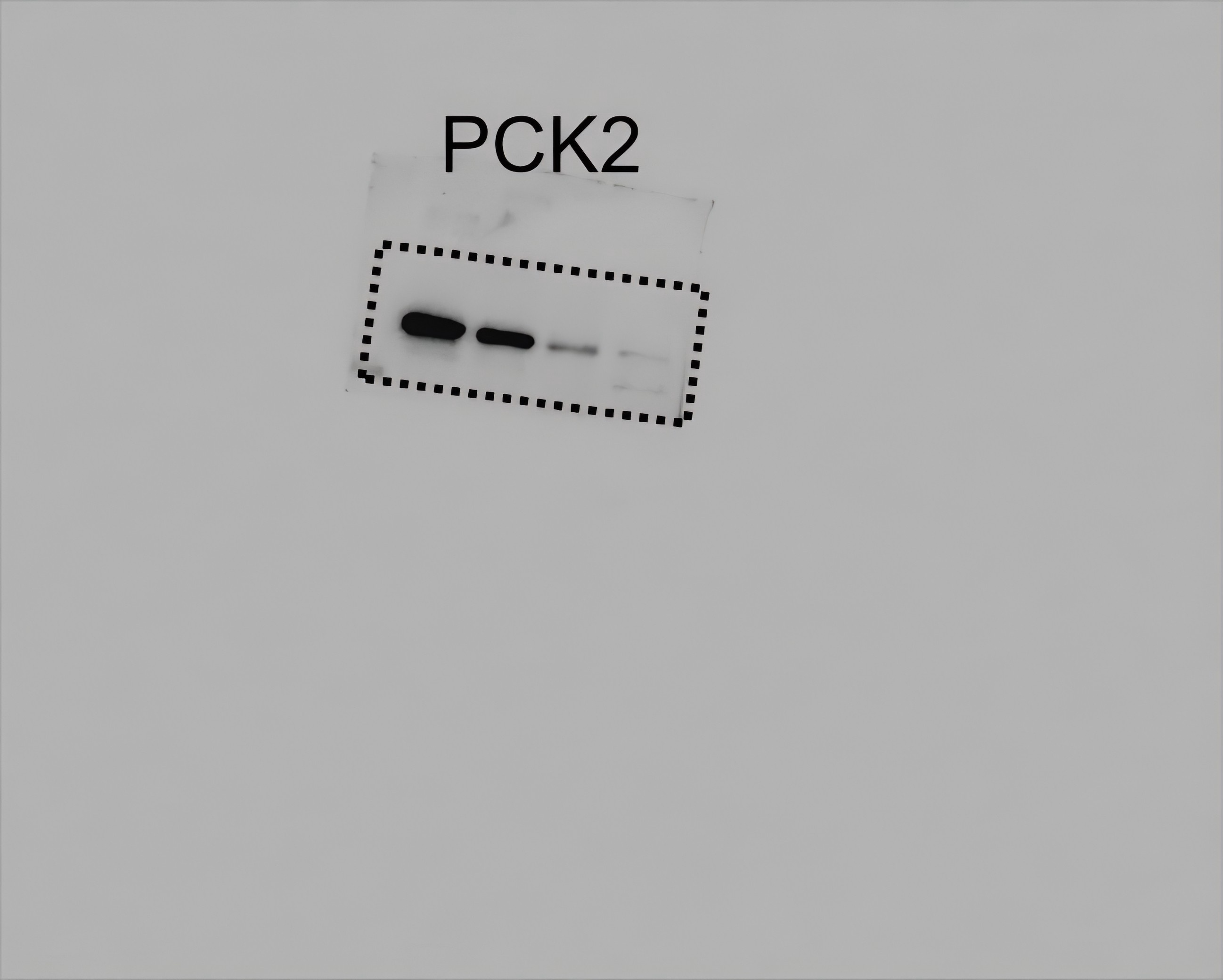

Supplement: Supplementary file 3 — Original Data [file 41419_2026_8662_MOESM3_ESM.zip › Original Data/Fig. 2D/7-PCK2.tif]

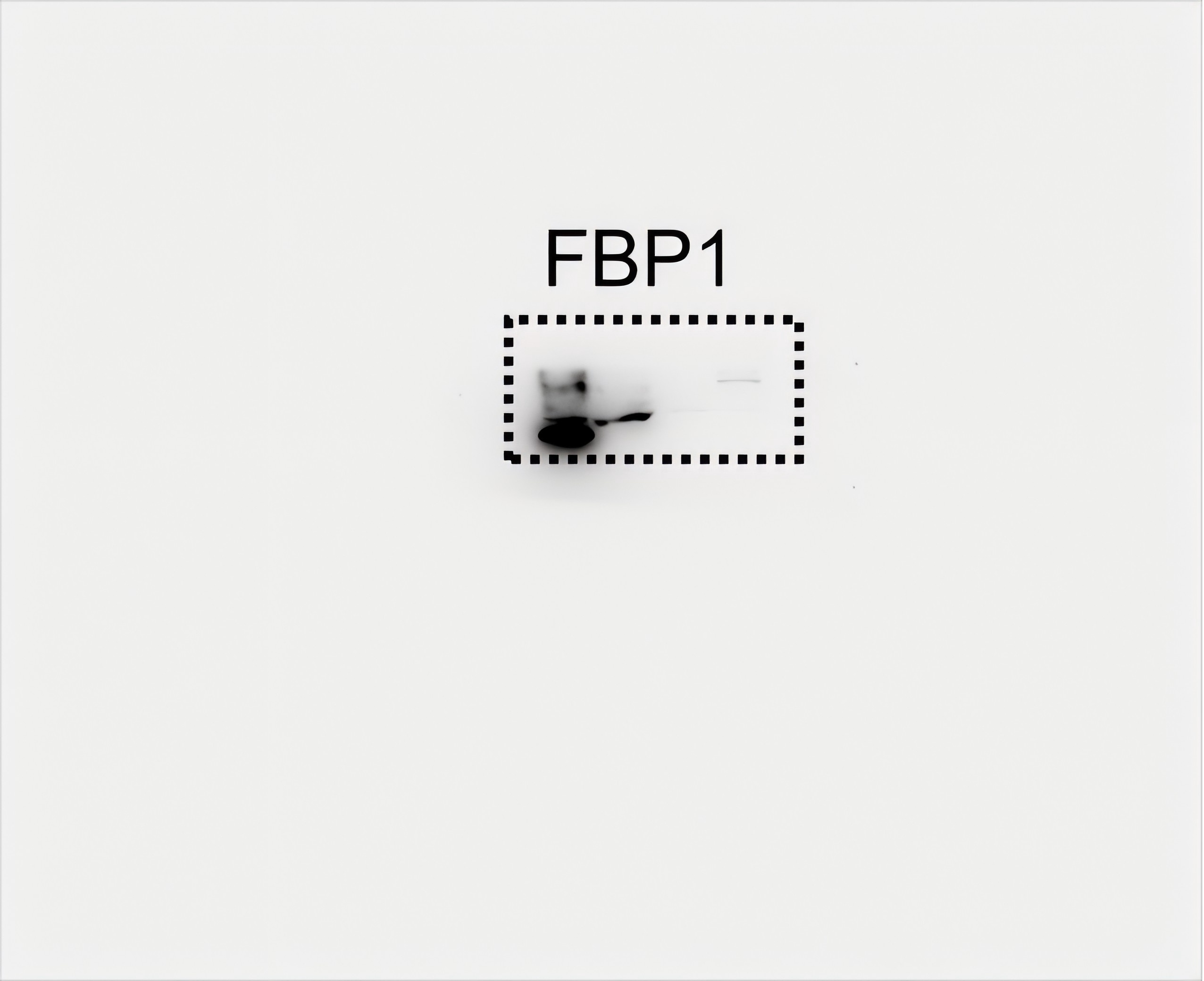

Supplement: Supplementary file 3 — Original Data [file 41419_2026_8662_MOESM3_ESM.zip › Original Data/Fig. 2D/8-FBP1.tif]

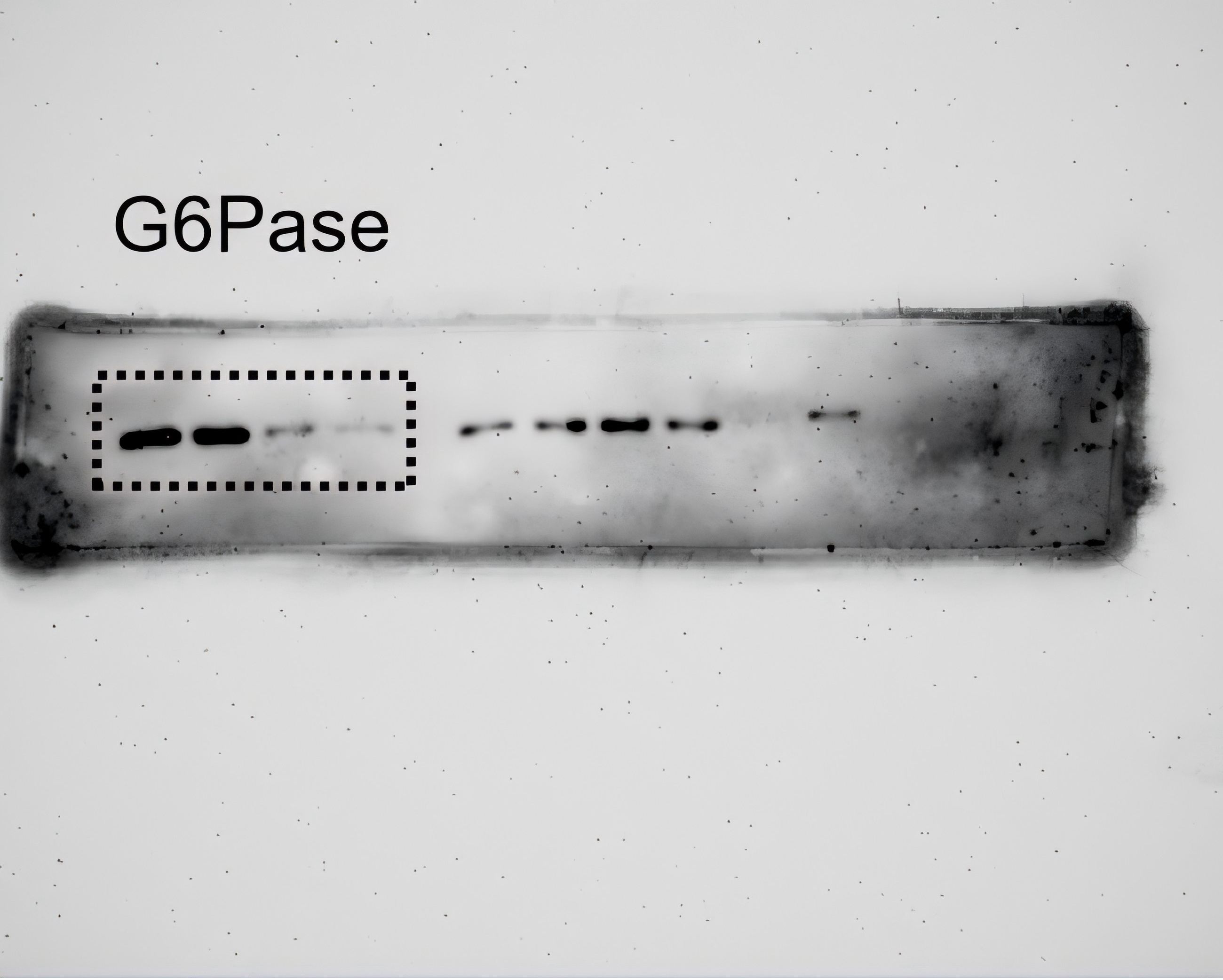

Supplement: Supplementary file 3 — Original Data [file 41419_2026_8662_MOESM3_ESM.zip › Original Data/Fig. 2D/9-G6Pase.tif]

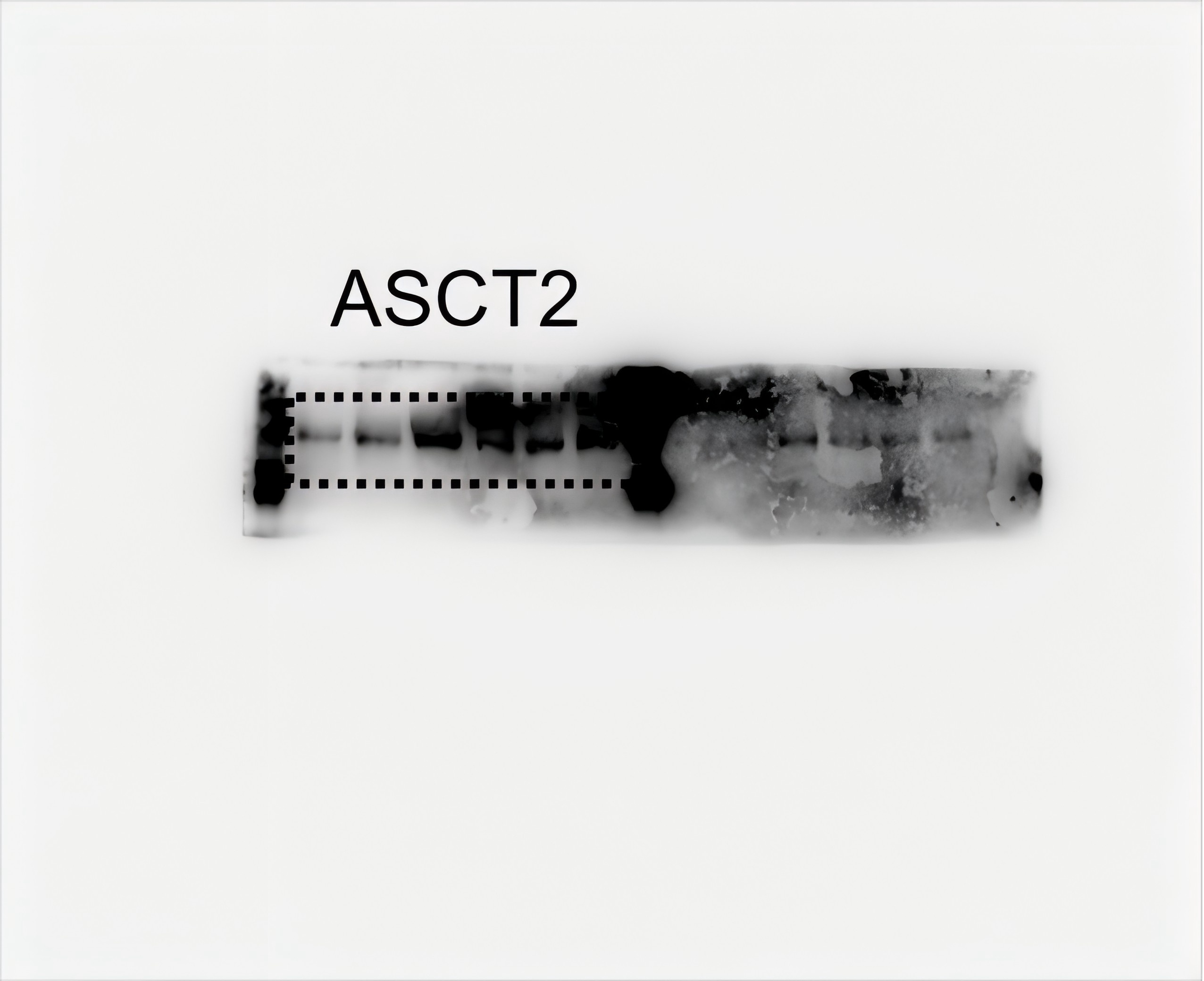

Supplement: Supplementary file 3 — Original Data [file 41419_2026_8662_MOESM3_ESM.zip › Original Data/Fig. 2E/1-ASCT2.tif]

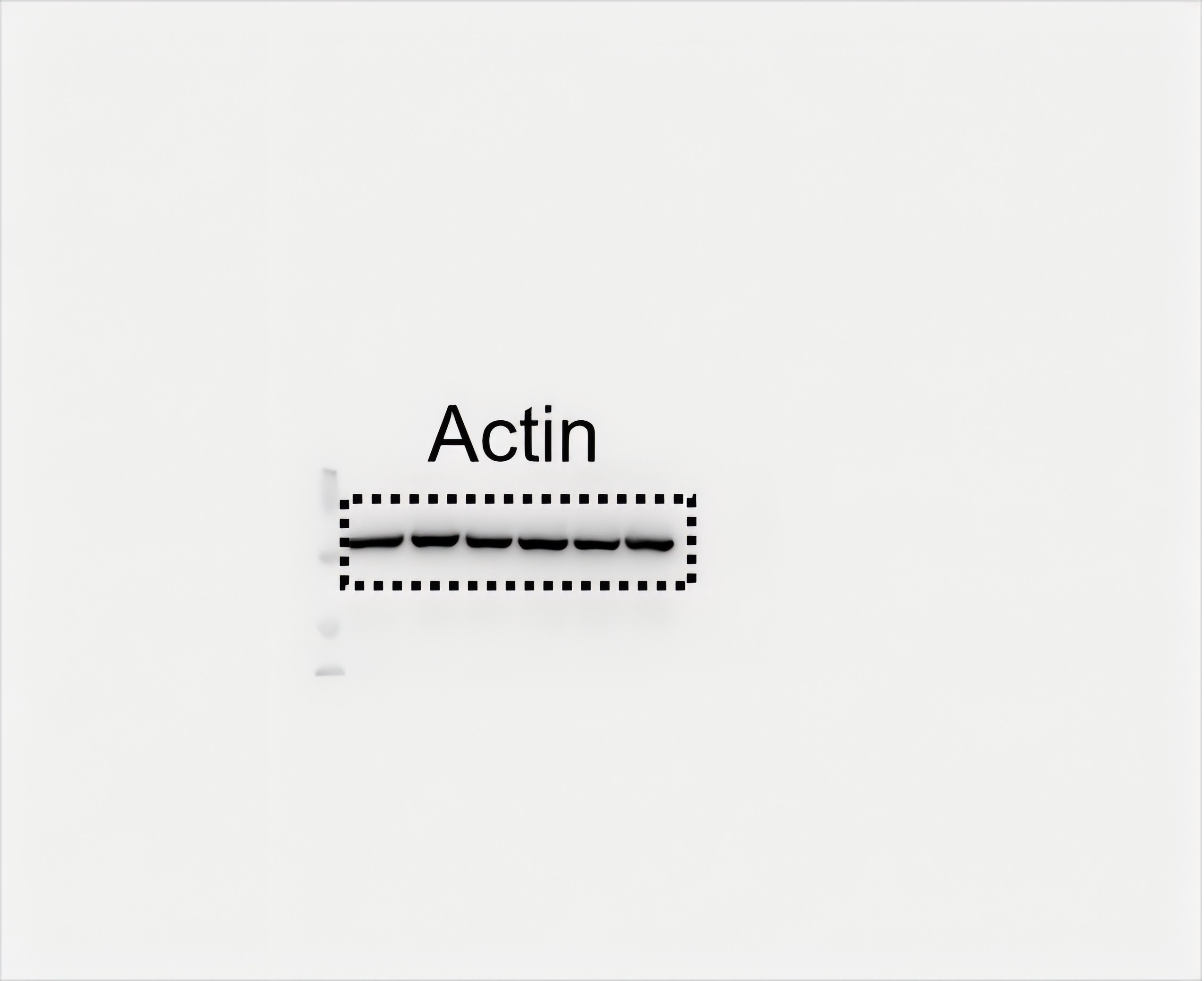

Supplement: Supplementary file 3 — Original Data [file 41419_2026_8662_MOESM3_ESM.zip › Original Data/Fig. 2E/10-Actin.tif]

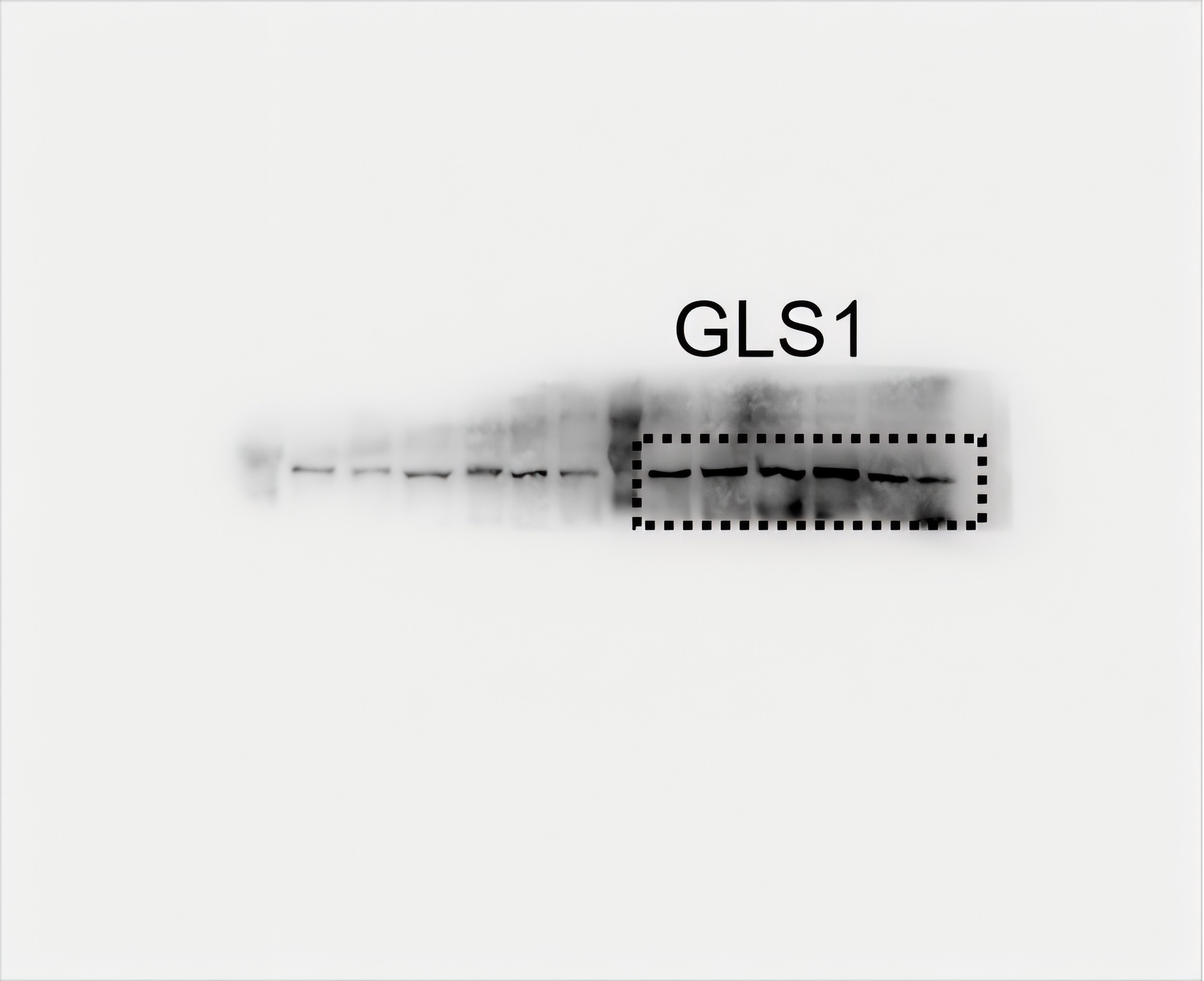

Supplement: Supplementary file 3 — Original Data [file 41419_2026_8662_MOESM3_ESM.zip › Original Data/Fig. 2E/2-GLS1.tif]

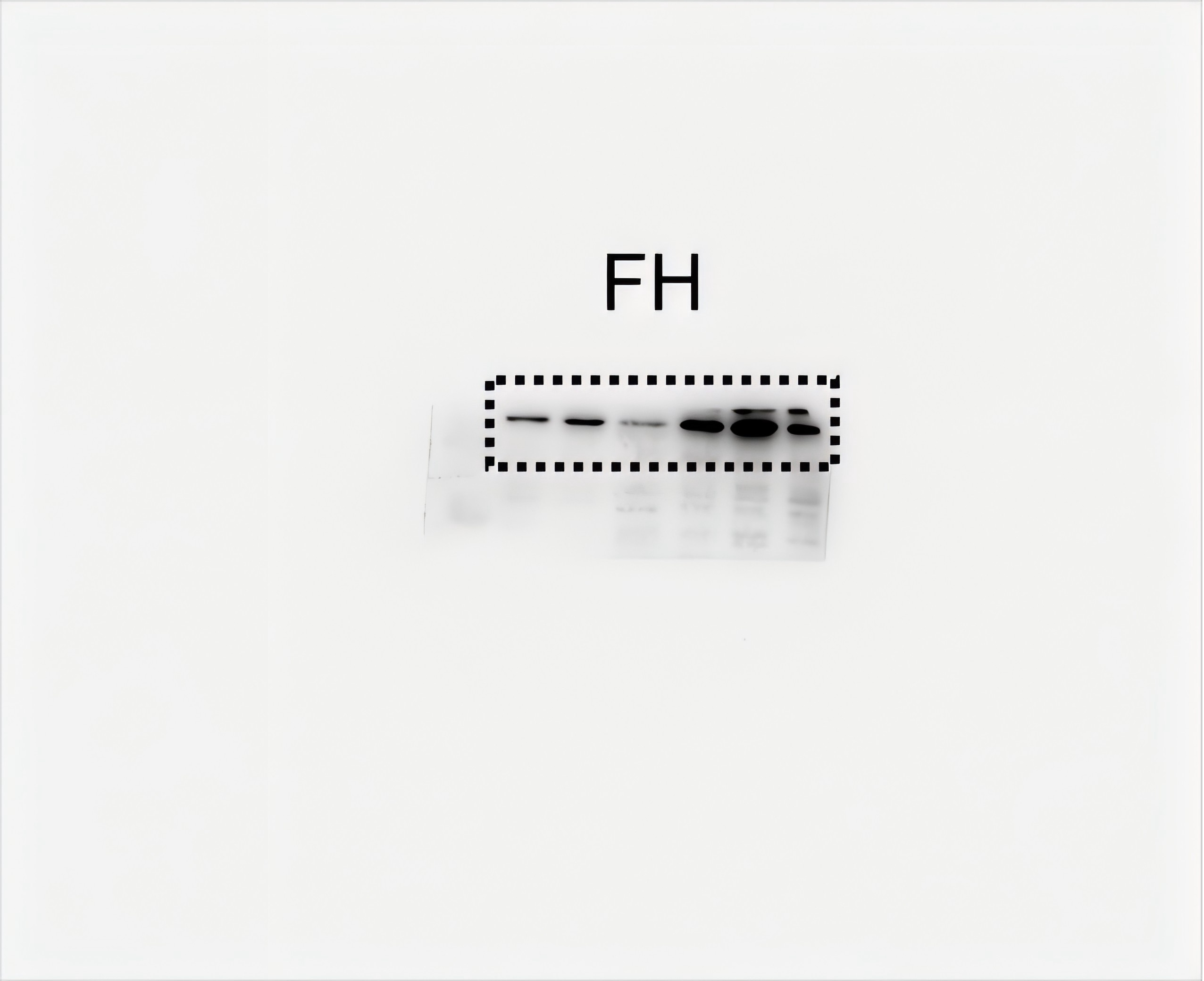

Supplement: Supplementary file 3 — Original Data [file 41419_2026_8662_MOESM3_ESM.zip › Original Data/Fig. 2E/3-FH.tif]

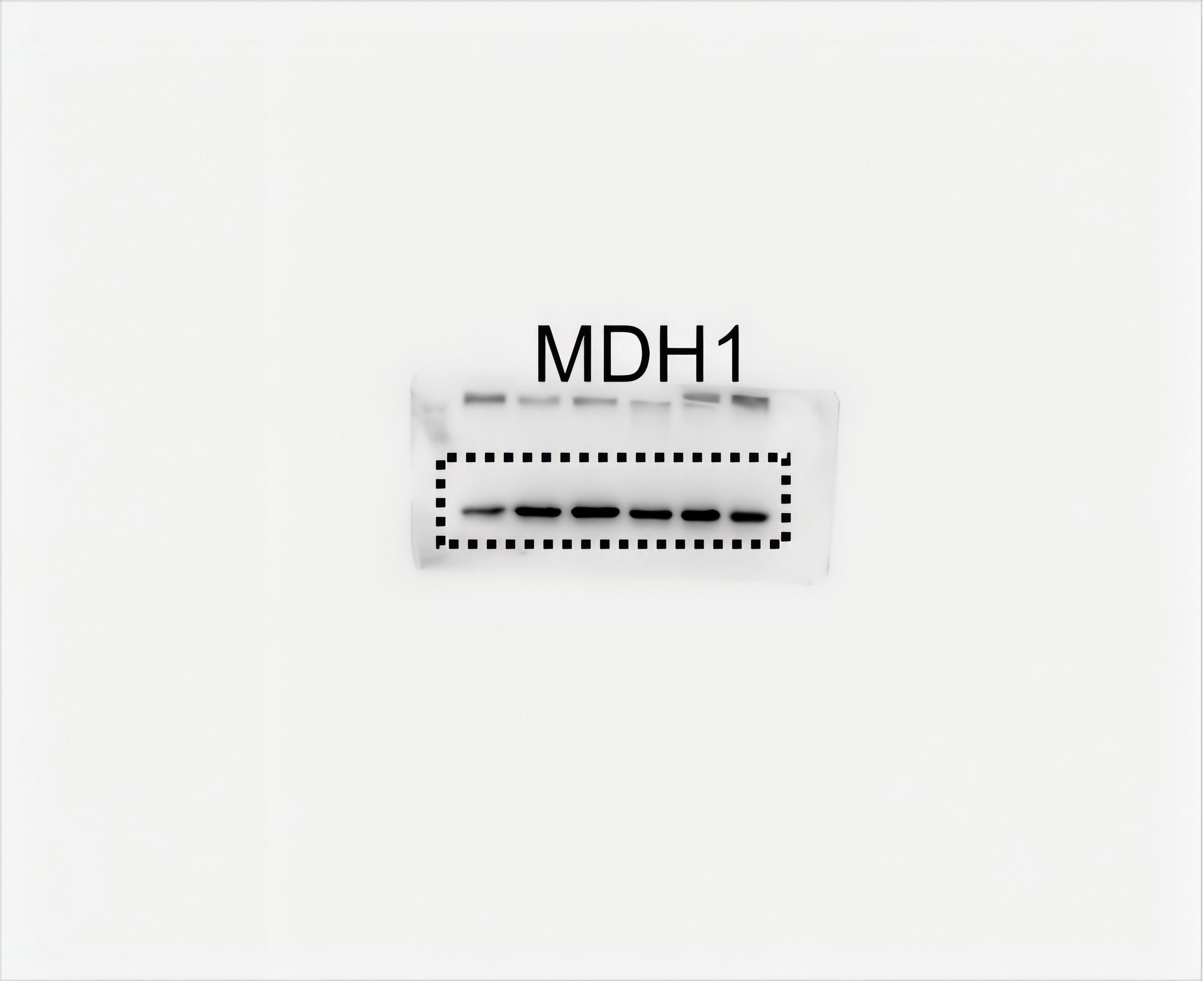

Supplement: Supplementary file 3 — Original Data [file 41419_2026_8662_MOESM3_ESM.zip › Original Data/Fig. 2E/4-MDH1.tif]

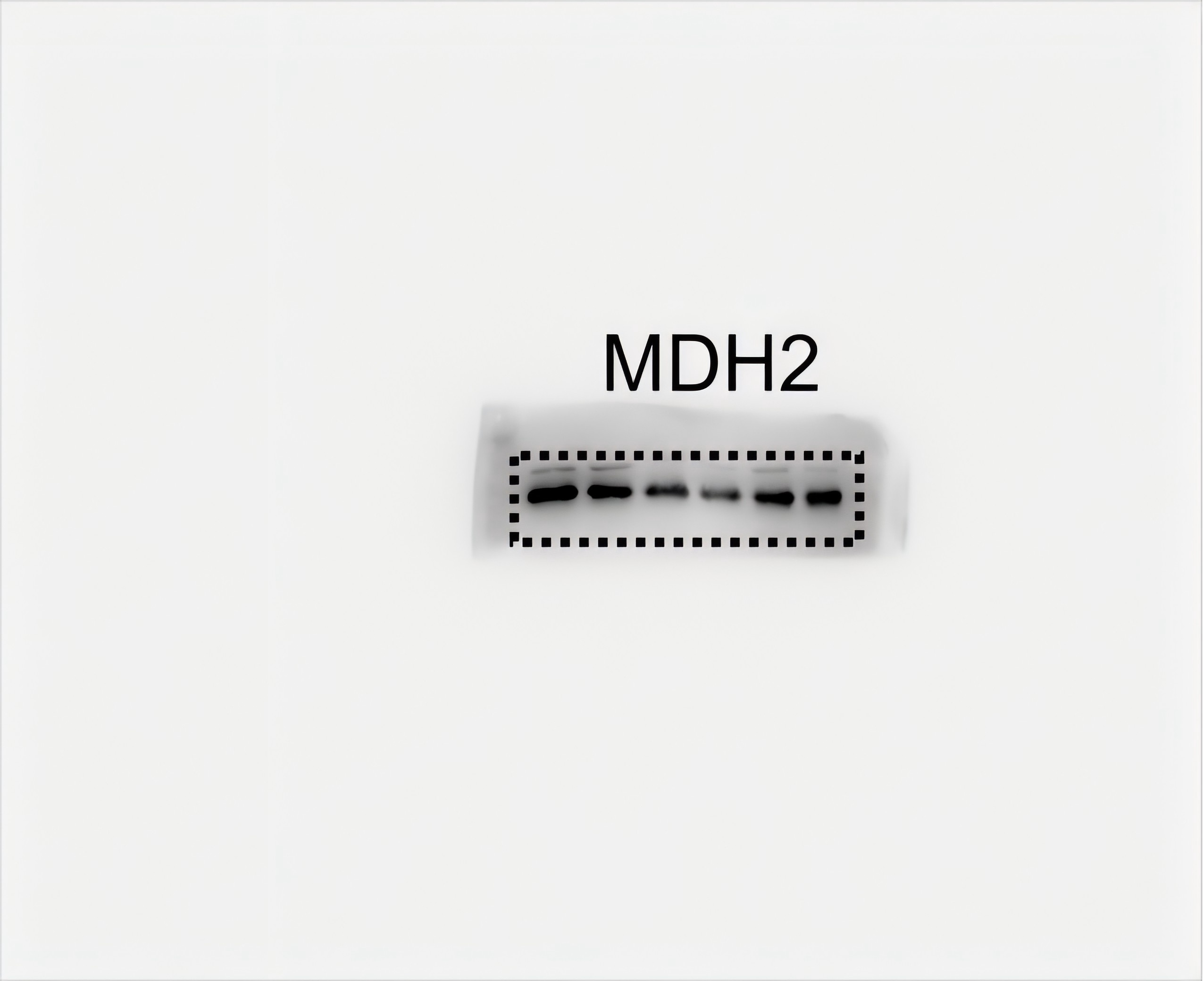

Supplement: Supplementary file 3 — Original Data [file 41419_2026_8662_MOESM3_ESM.zip › Original Data/Fig. 2E/5-MDH2.tif]

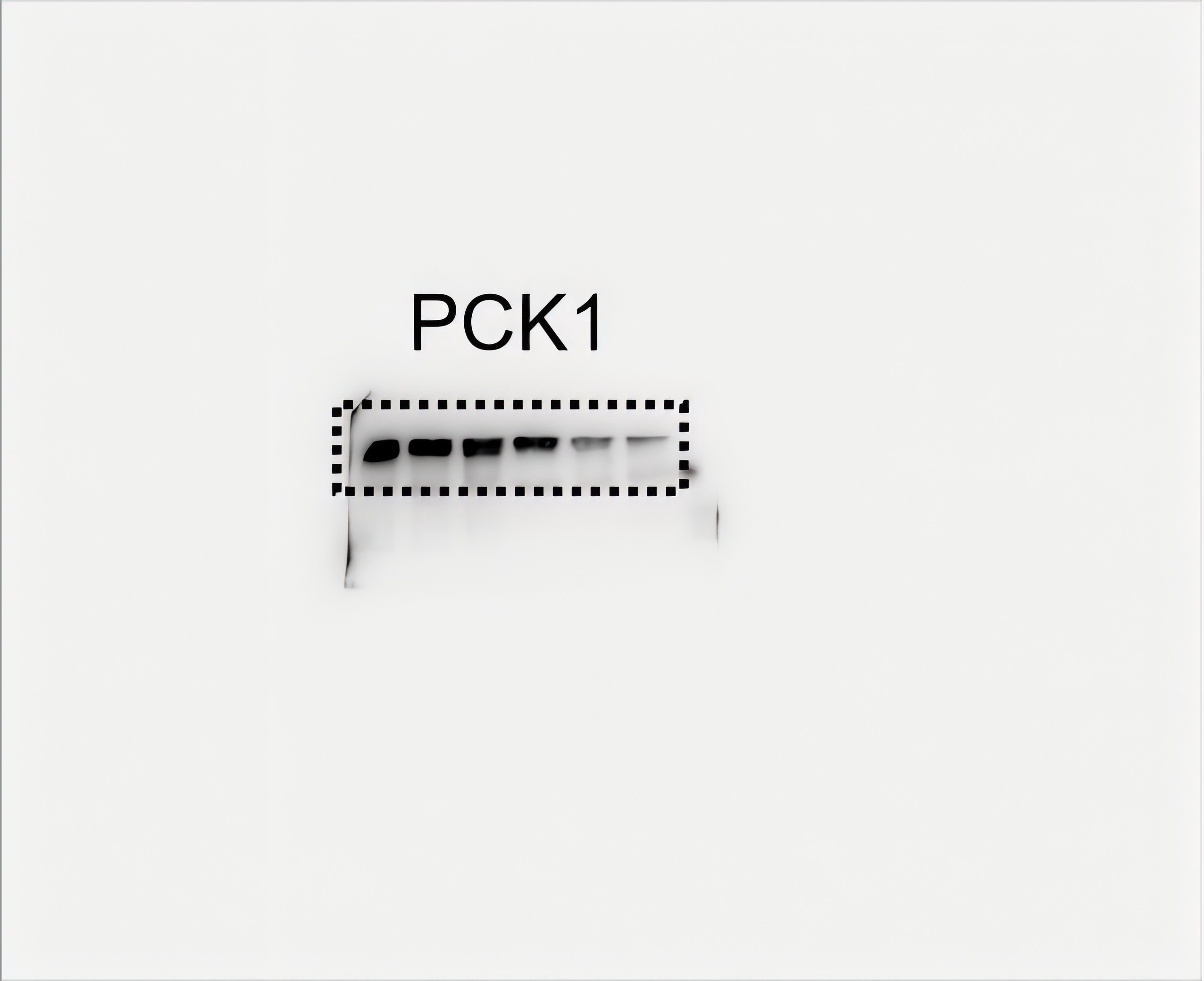

Supplement: Supplementary file 3 — Original Data [file 41419_2026_8662_MOESM3_ESM.zip › Original Data/Fig. 2E/6-PCK1.tif]

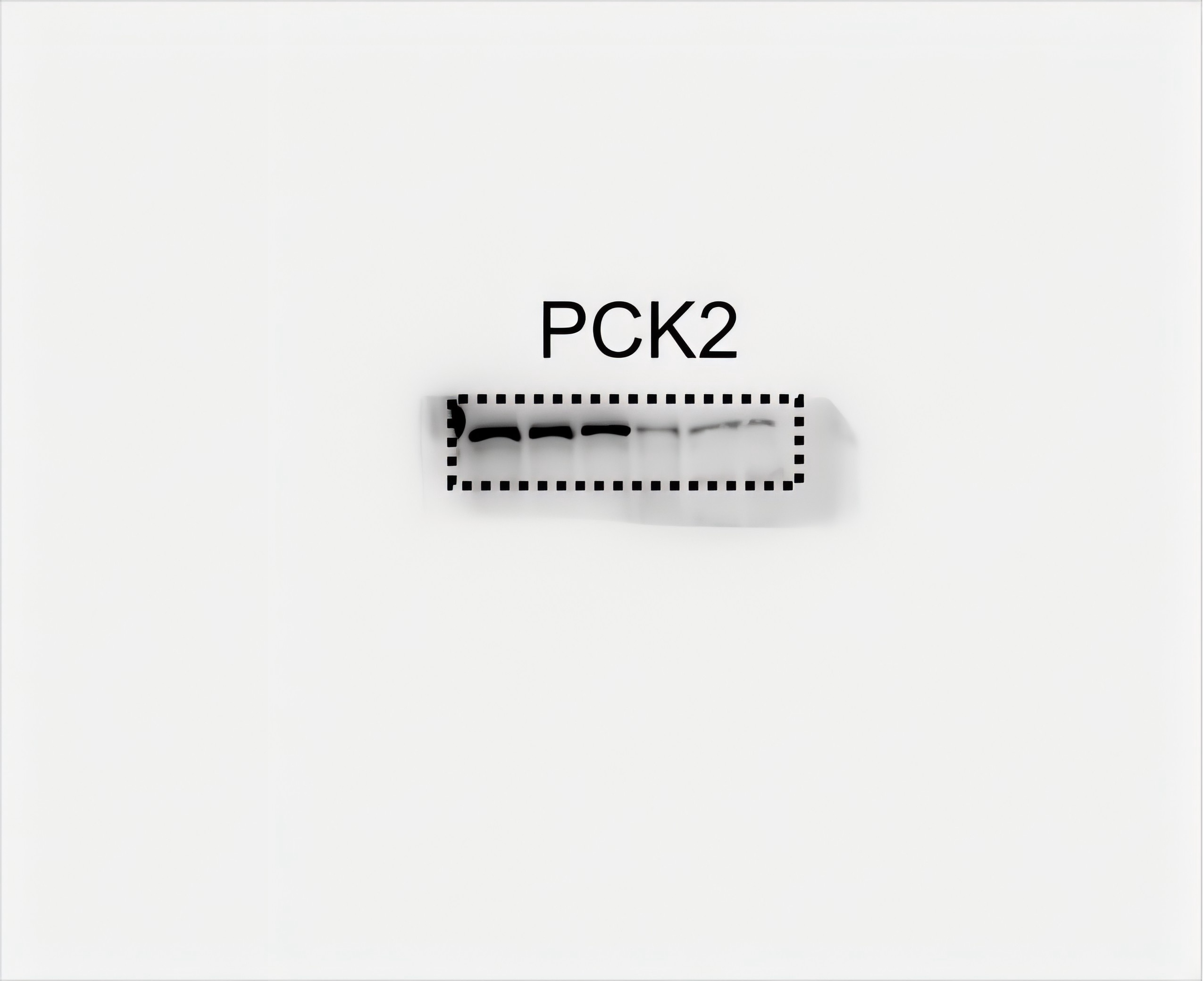

Supplement: Supplementary file 3 — Original Data [file 41419_2026_8662_MOESM3_ESM.zip › Original Data/Fig. 2E/7-PCK2.tif]

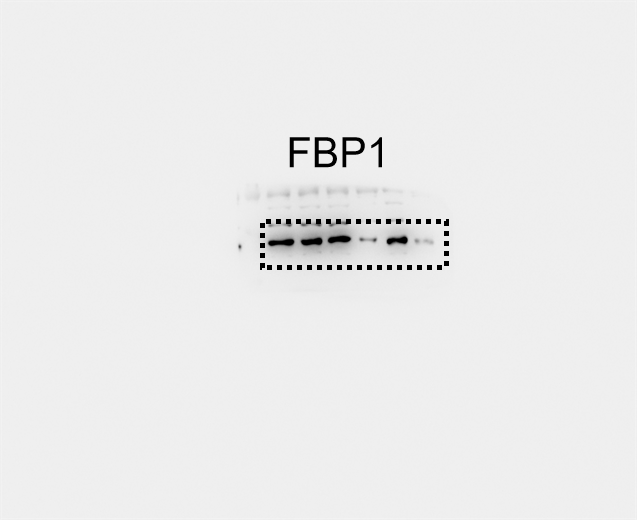

Supplement: Supplementary file 3 — Original Data [file 41419_2026_8662_MOESM3_ESM.zip › Original Data/Fig. 2E/8-FBP1.tif]

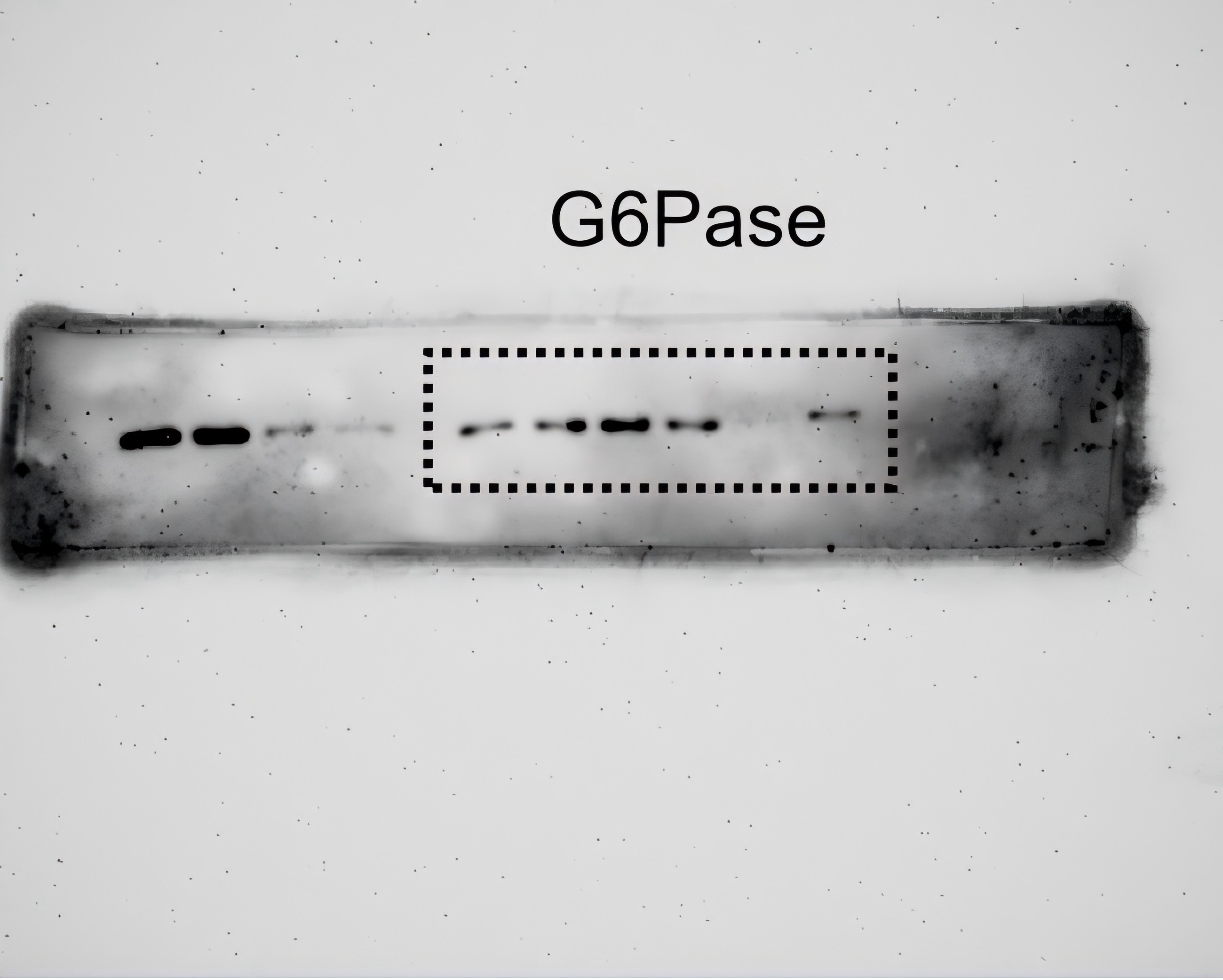

Supplement: Supplementary file 3 — Original Data [file 41419_2026_8662_MOESM3_ESM.zip › Original Data/Fig. 2E/9-G6Pase.tif]

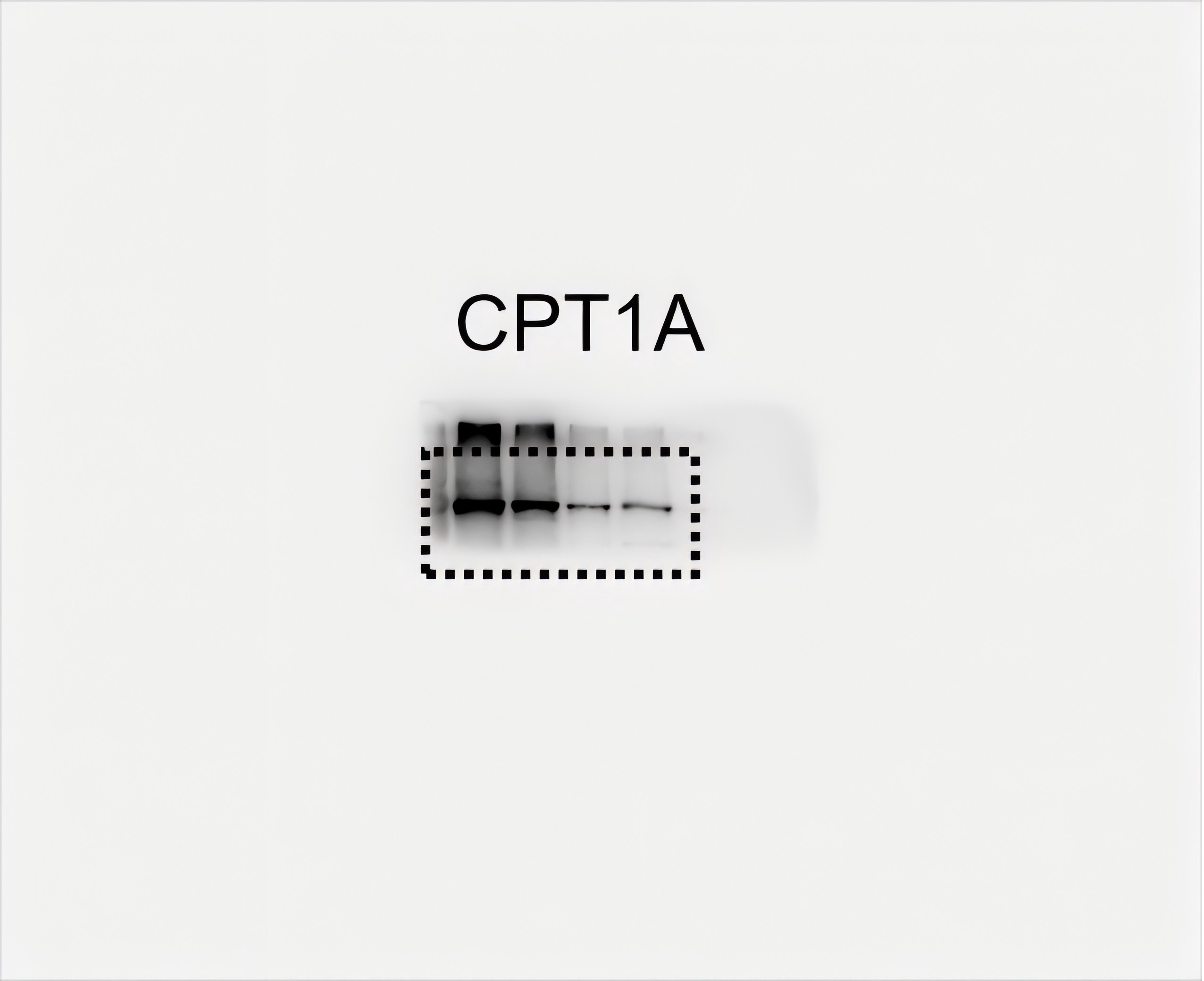

Supplement: Supplementary file 3 — Original Data [file 41419_2026_8662_MOESM3_ESM.zip › Original Data/Fig. 3C/1-CPT1A.tif]

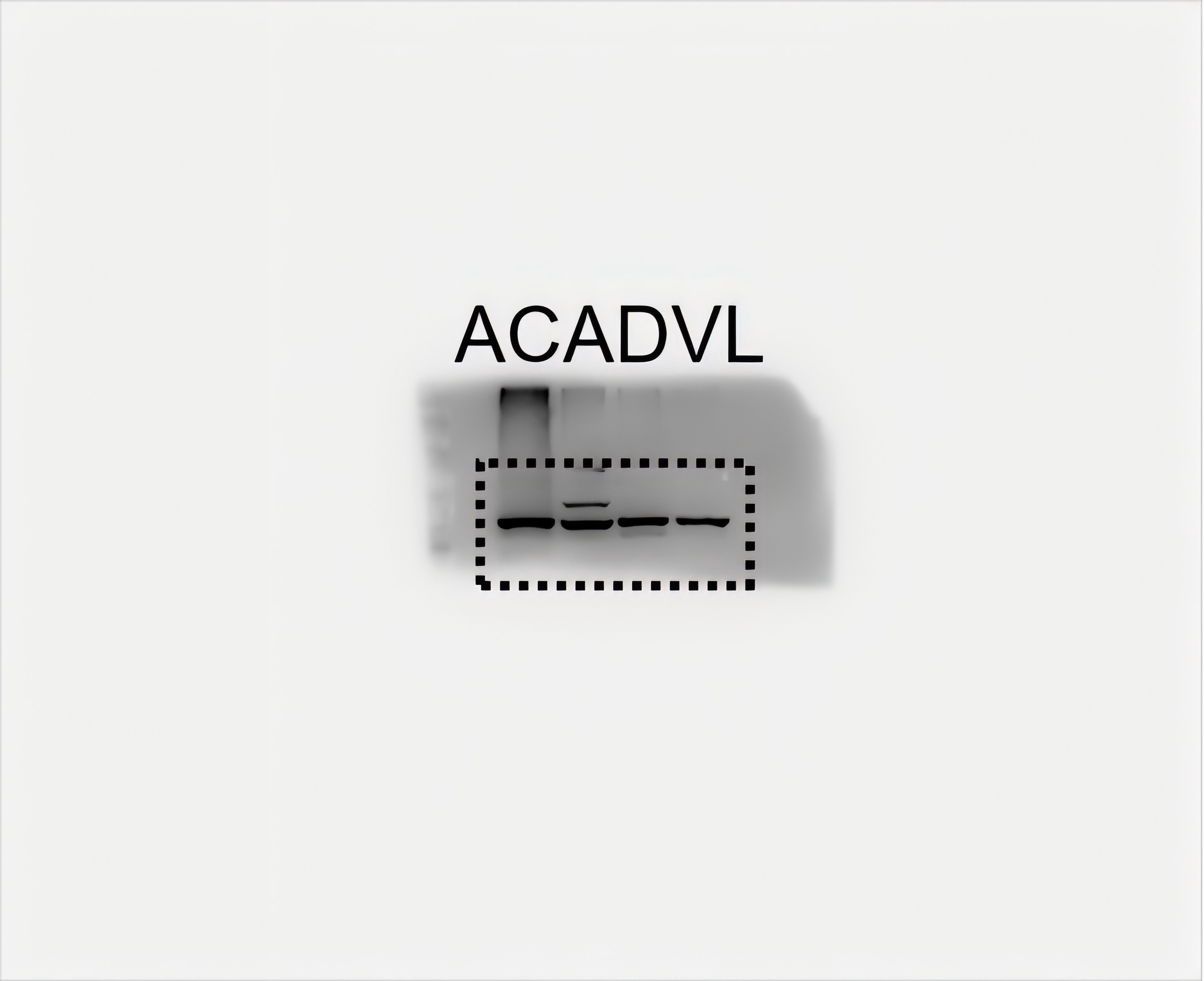

Supplement: Supplementary file 3 — Original Data [file 41419_2026_8662_MOESM3_ESM.zip › Original Data/Fig. 3C/2-ACADVL.tif]

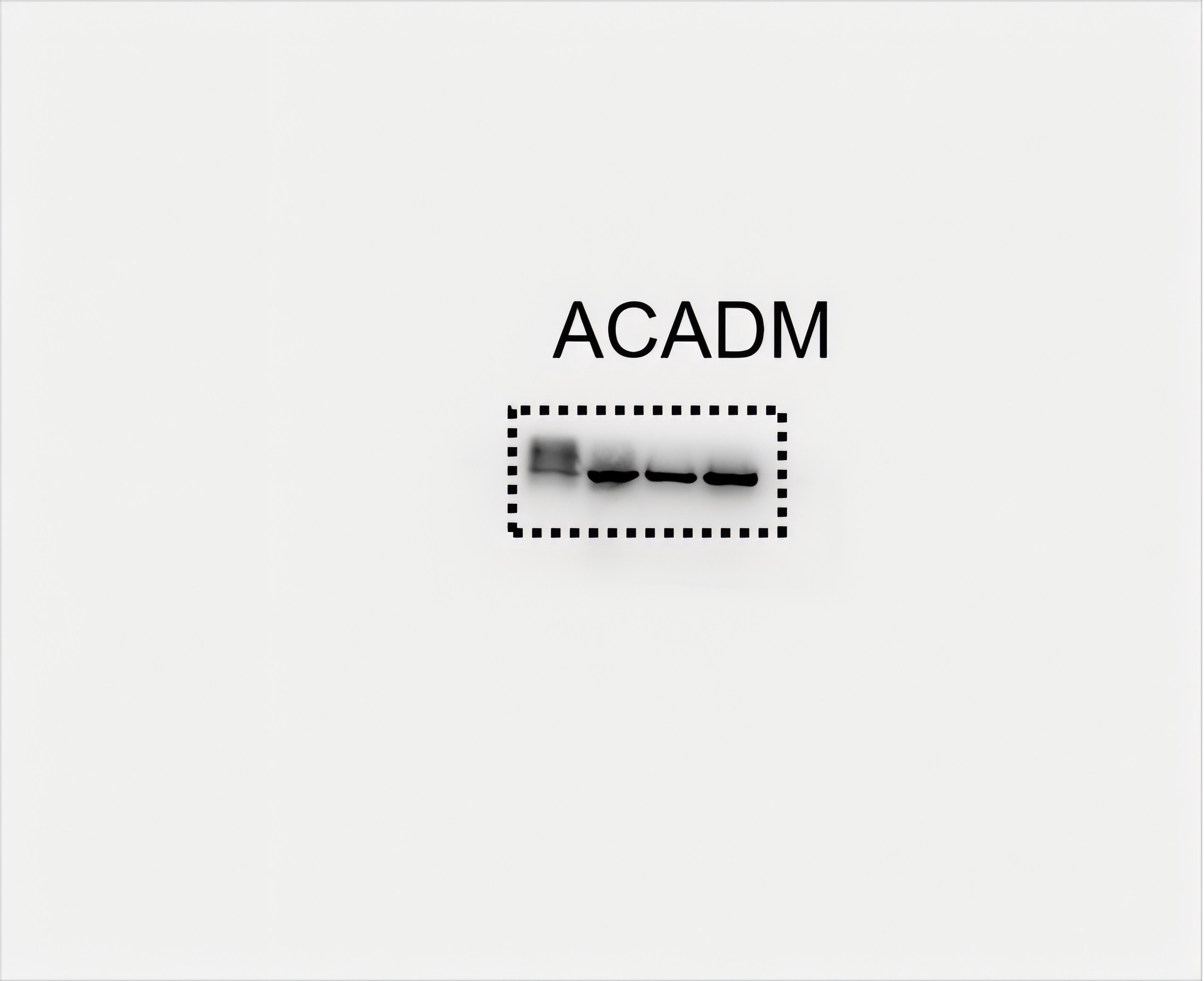

Supplement: Supplementary file 3 — Original Data [file 41419_2026_8662_MOESM3_ESM.zip › Original Data/Fig. 3C/3-ACADM.tif]

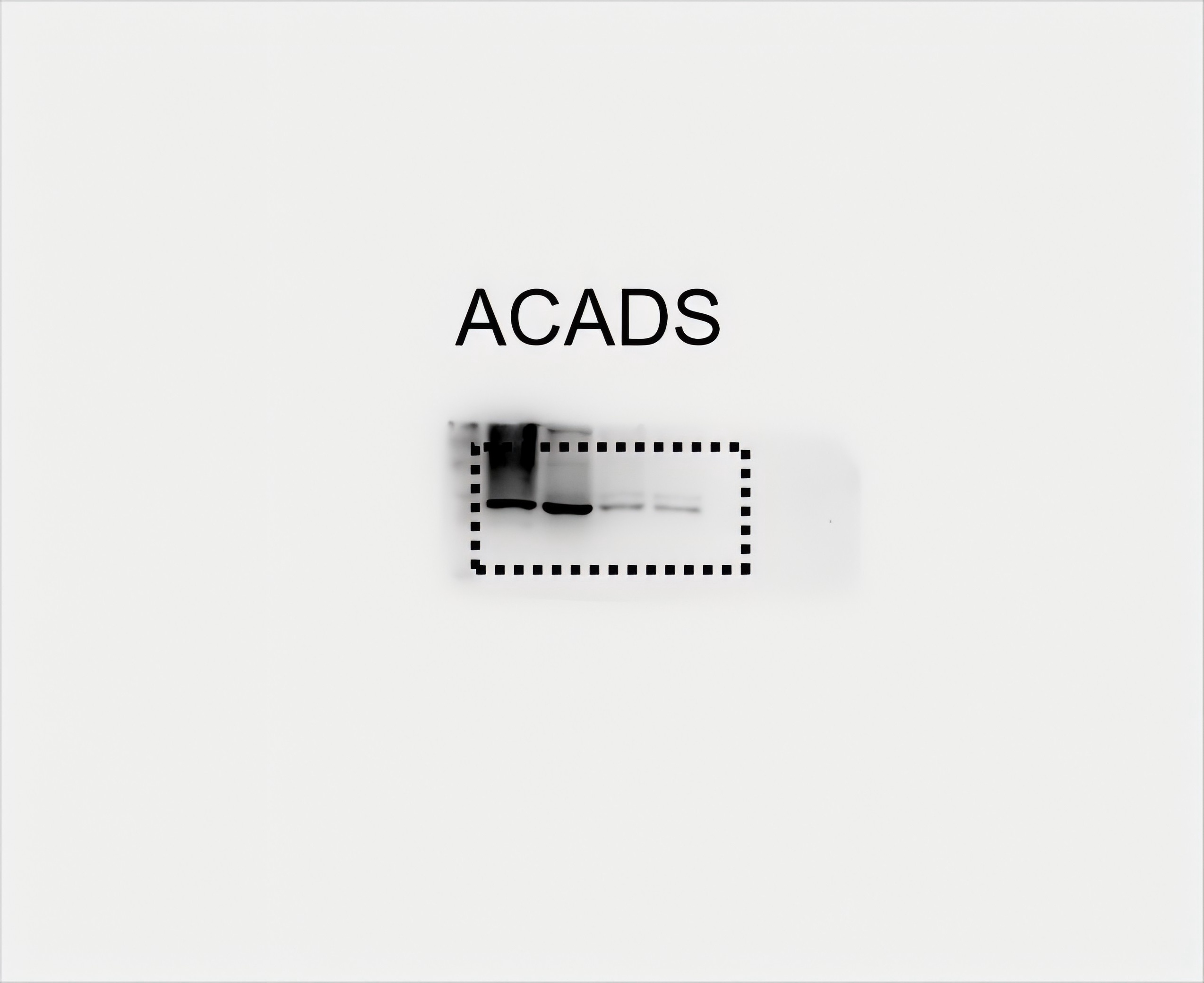

Supplement: Supplementary file 3 — Original Data [file 41419_2026_8662_MOESM3_ESM.zip › Original Data/Fig. 3C/4-ACADS.tif]

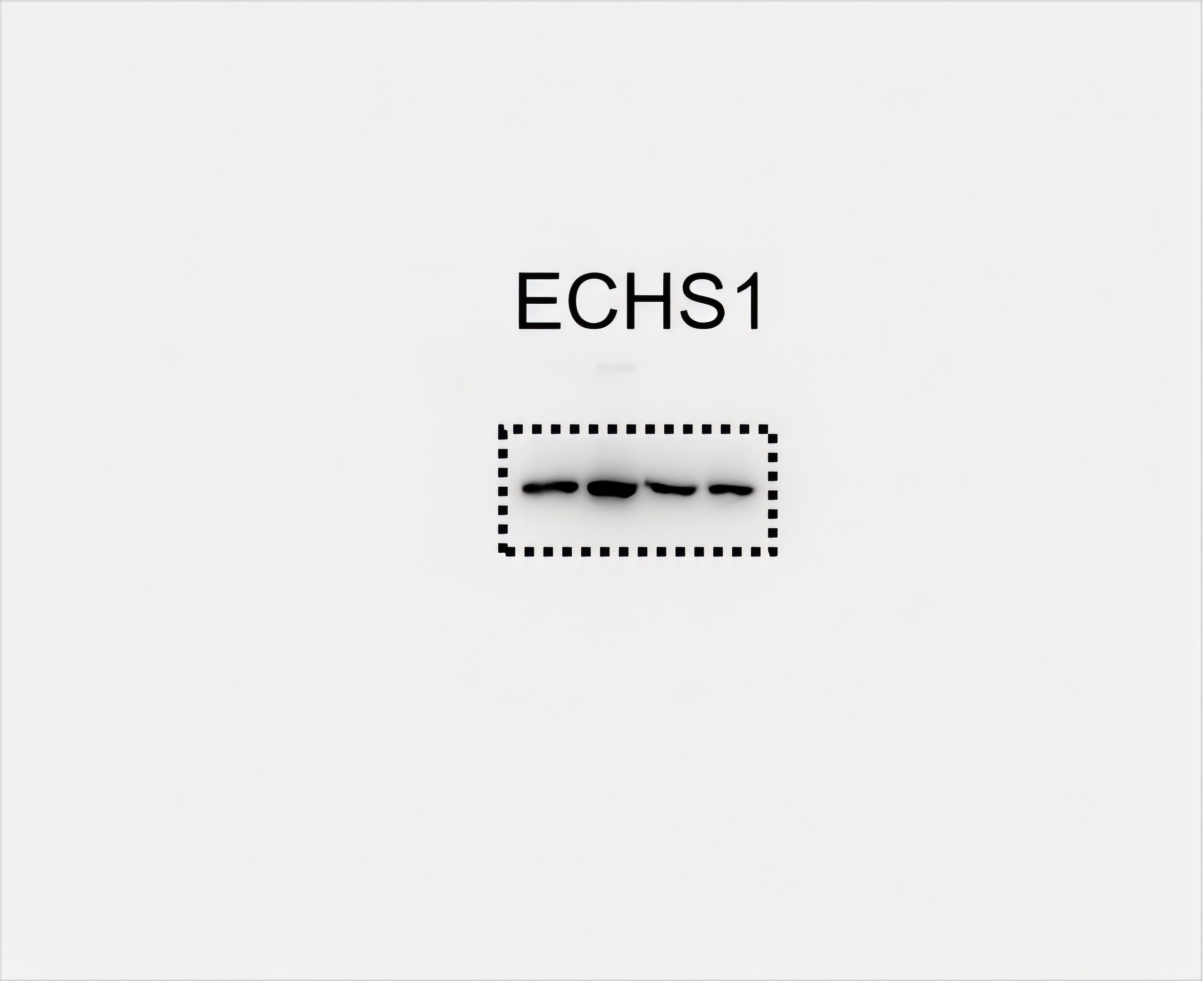

Supplement: Supplementary file 3 — Original Data [file 41419_2026_8662_MOESM3_ESM.zip › Original Data/Fig. 3C/5-ECHS1.tif]

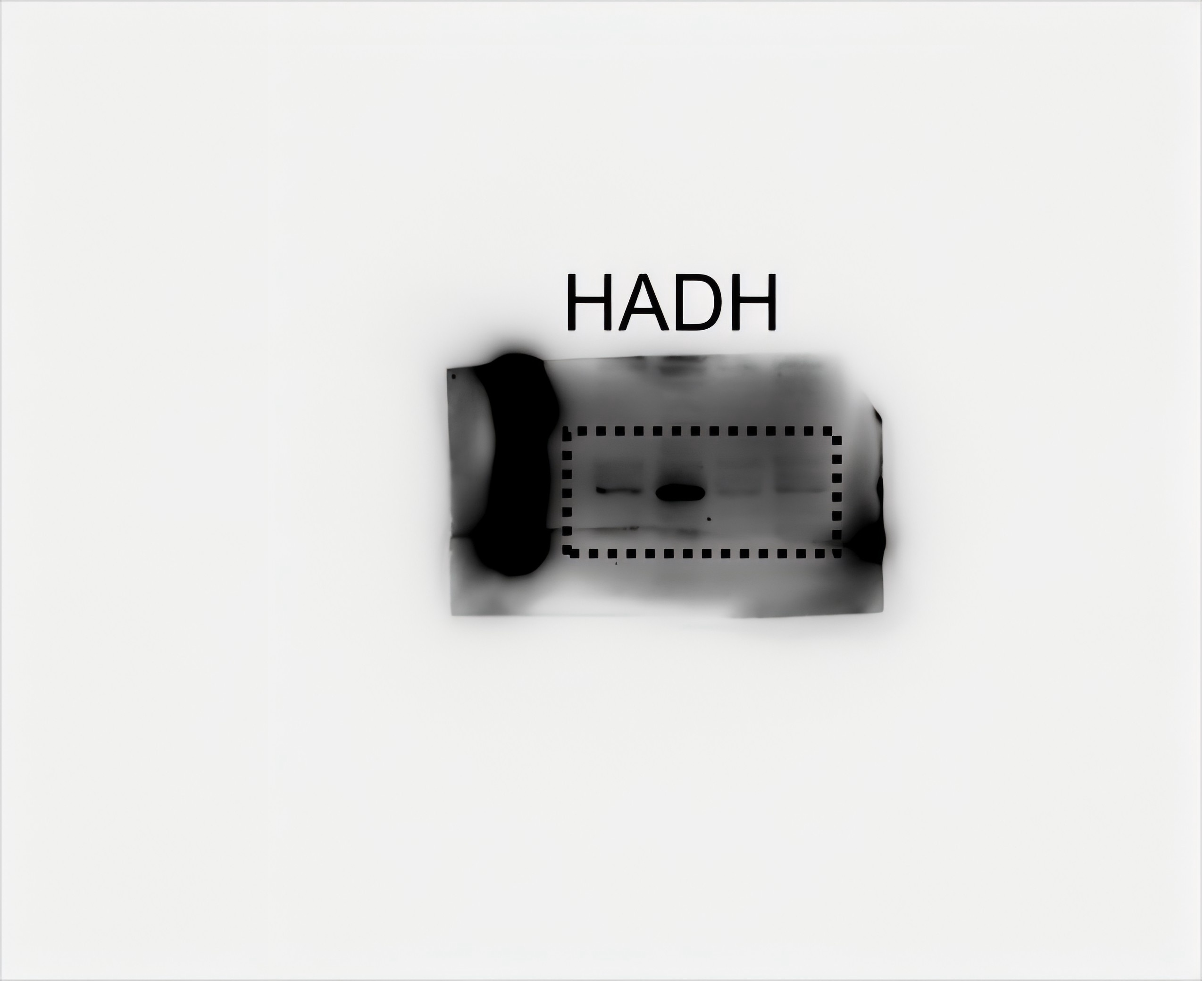

Supplement: Supplementary file 3 — Original Data [file 41419_2026_8662_MOESM3_ESM.zip › Original Data/Fig. 3C/6-HADH.tif]

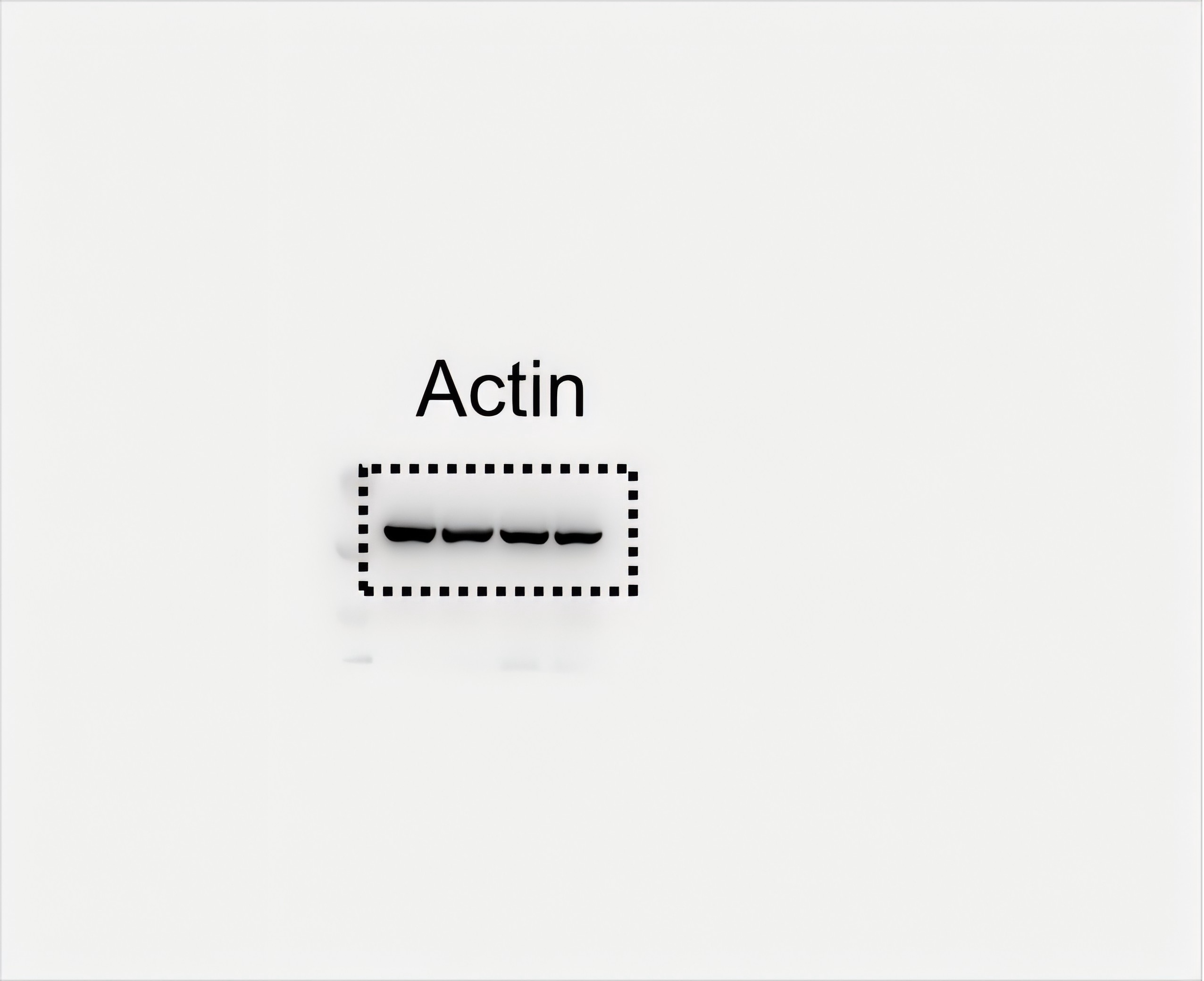

Supplement: Supplementary file 3 — Original Data [file 41419_2026_8662_MOESM3_ESM.zip › Original Data/Fig. 3C/7-Actin.tif]

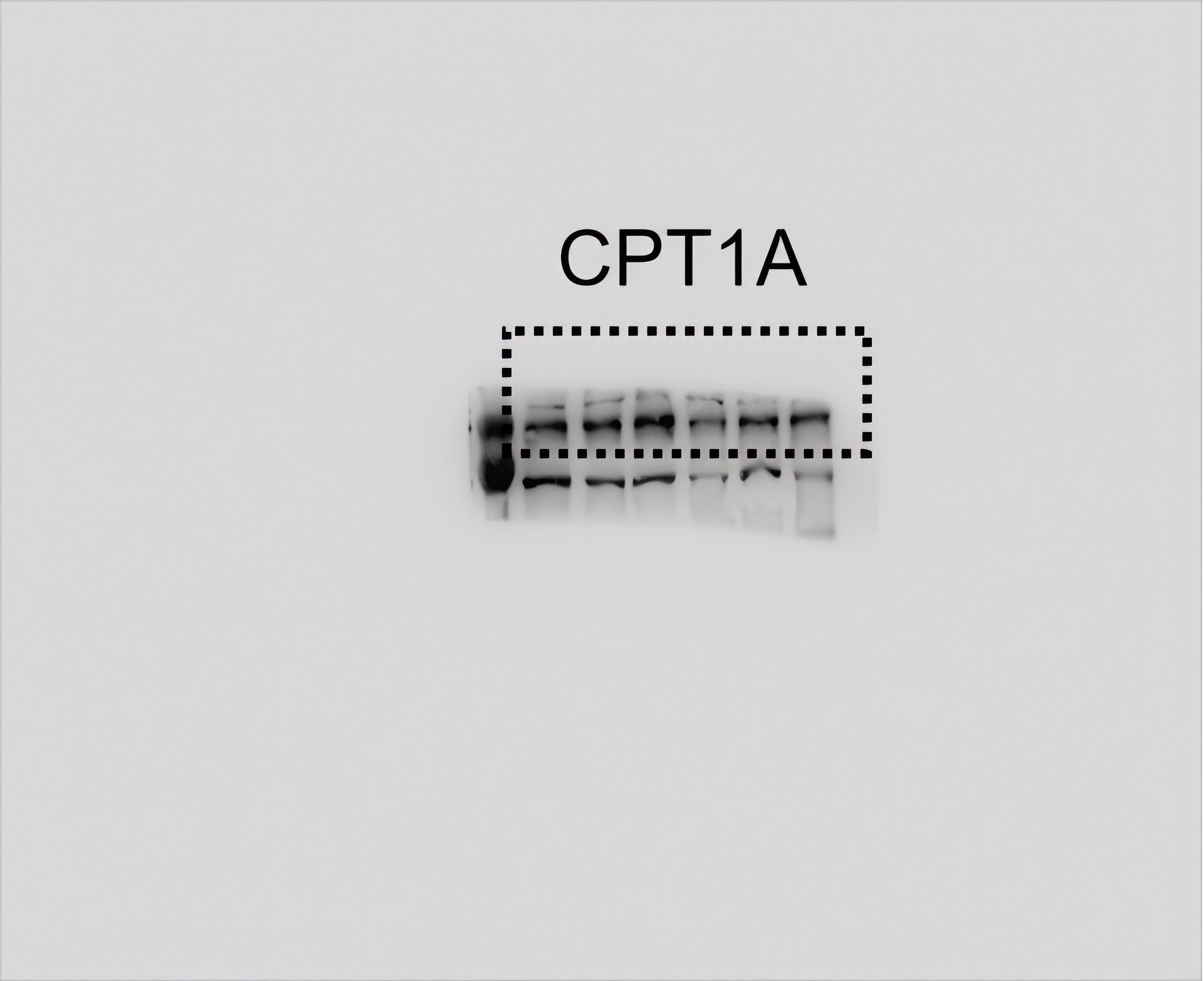

Supplement: Supplementary file 3 — Original Data [file 41419_2026_8662_MOESM3_ESM.zip › Original Data/Fig. 3D/1-CPT1A.tif]

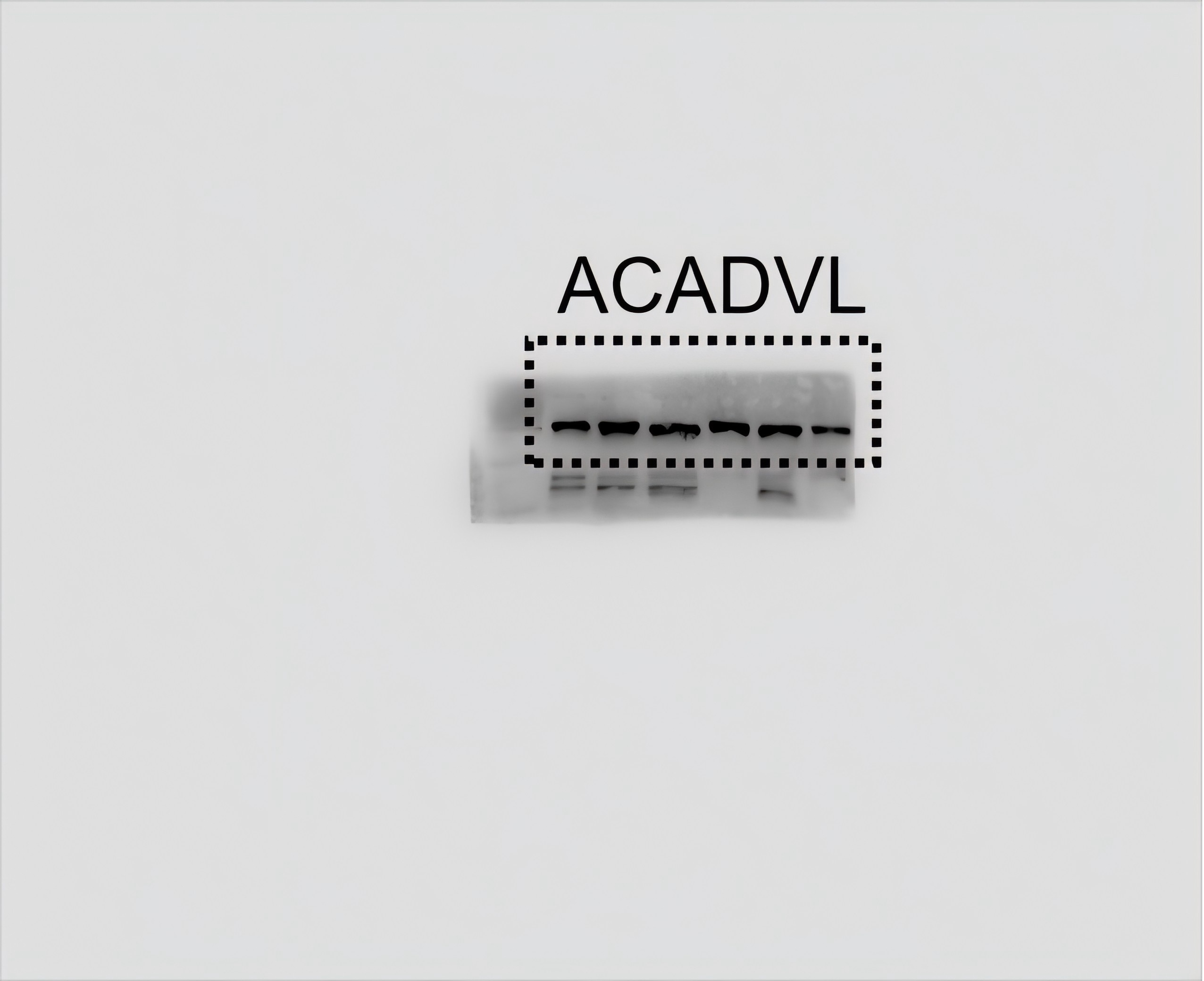

Supplement: Supplementary file 3 — Original Data [file 41419_2026_8662_MOESM3_ESM.zip › Original Data/Fig. 3D/2-ACADVL.tif]

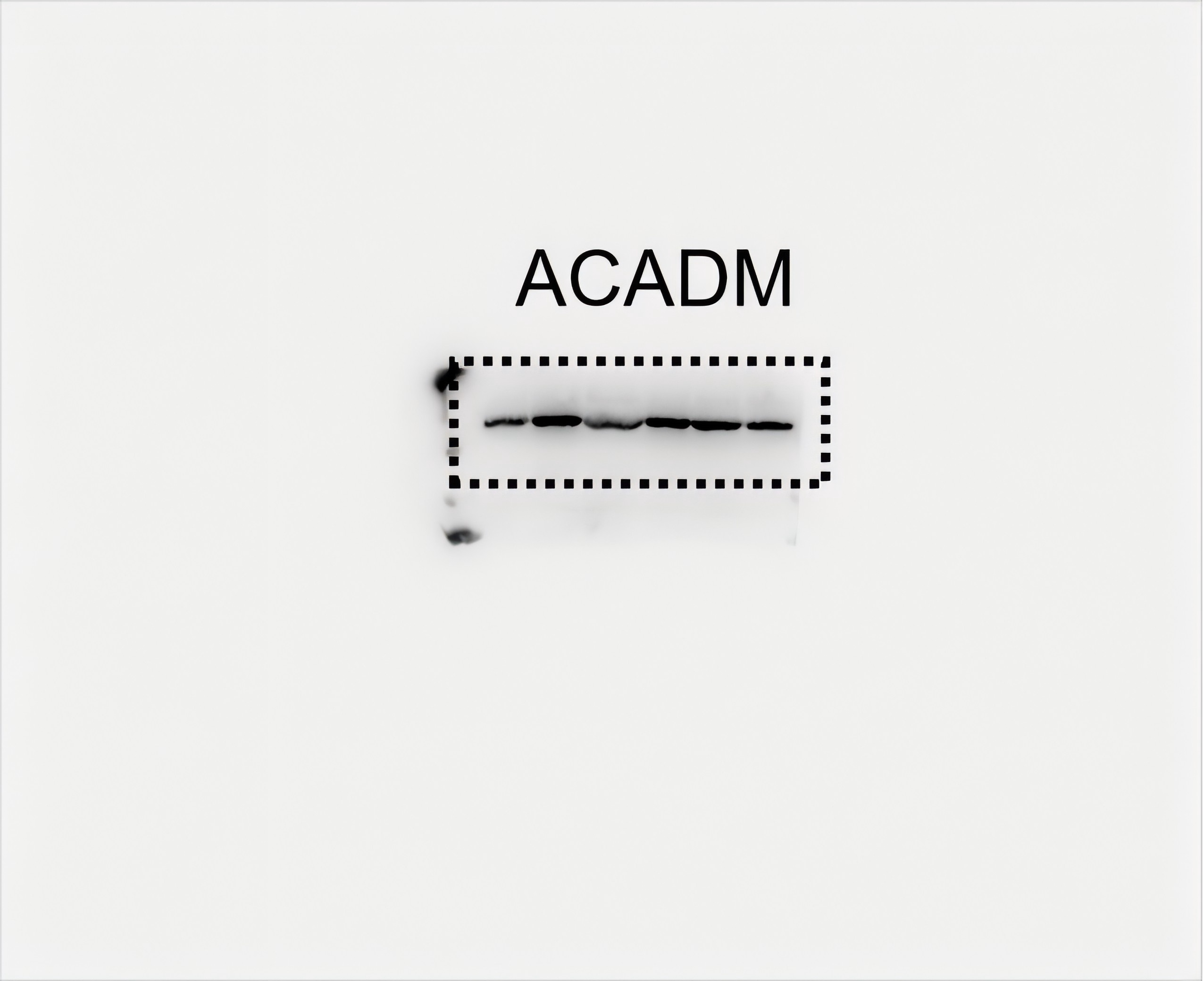

Supplement: Supplementary file 3 — Original Data [file 41419_2026_8662_MOESM3_ESM.zip › Original Data/Fig. 3D/3-ACADM.tif]

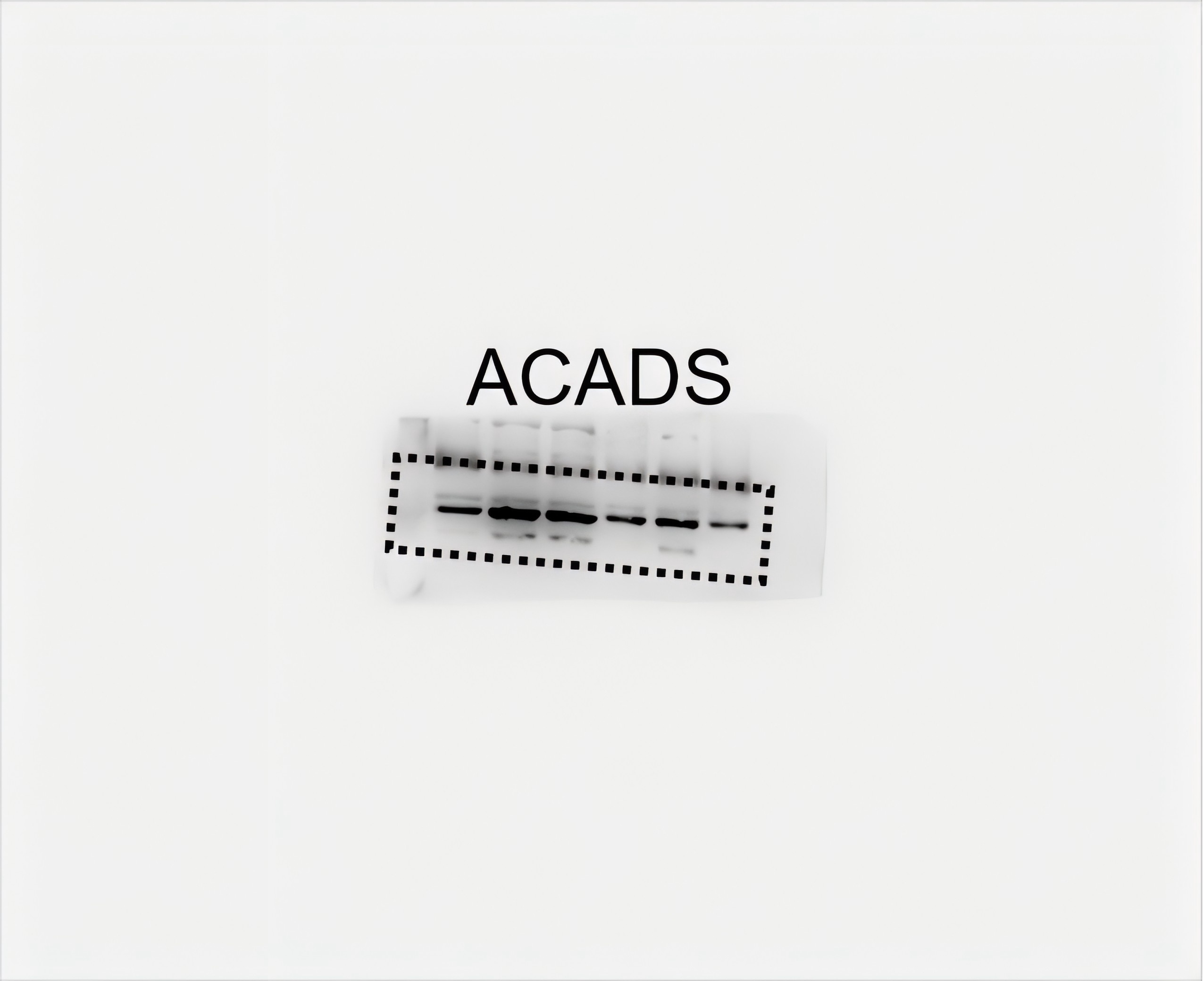

Supplement: Supplementary file 3 — Original Data [file 41419_2026_8662_MOESM3_ESM.zip › Original Data/Fig. 3D/4-ACADS.tif]

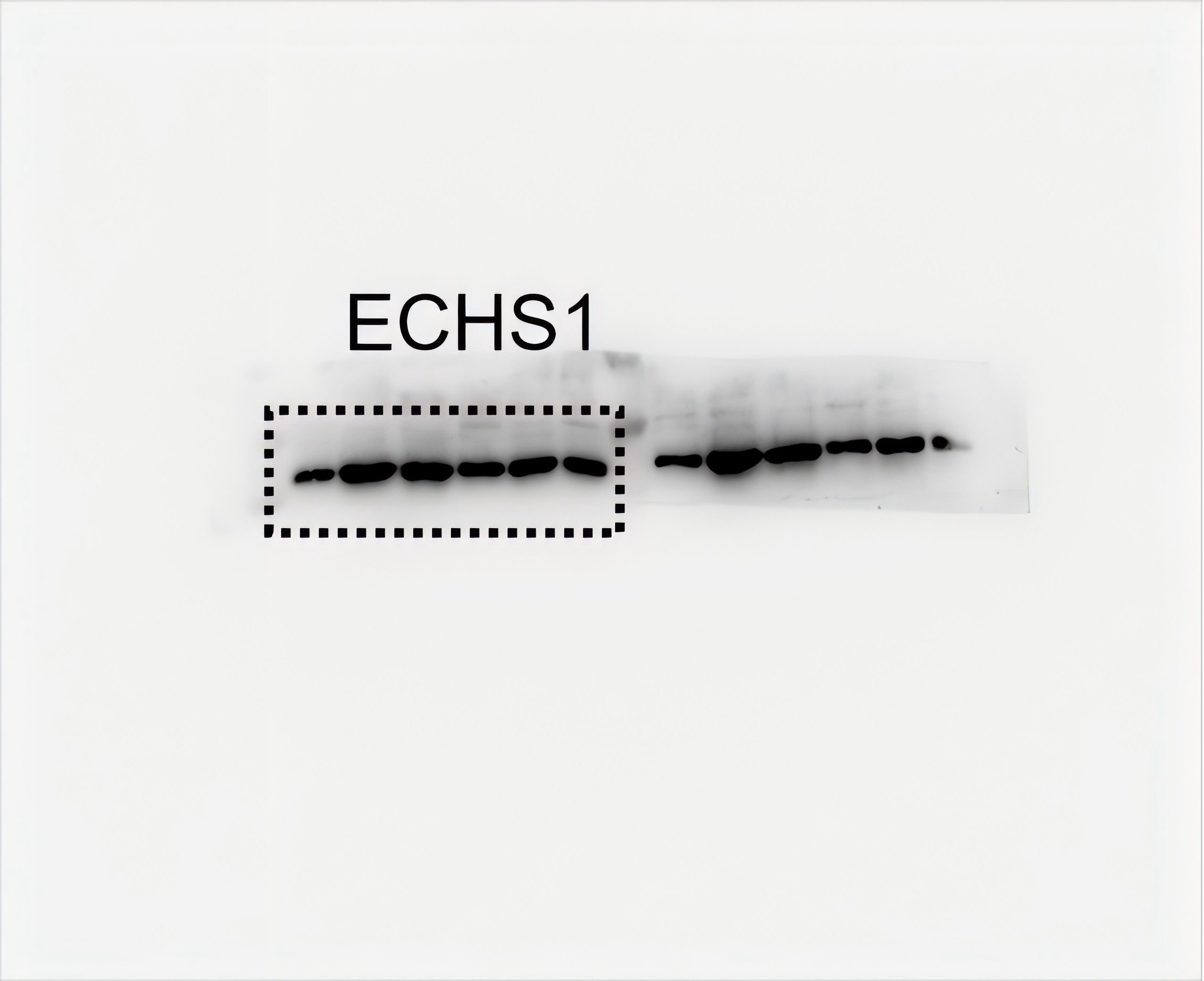

Supplement: Supplementary file 3 — Original Data [file 41419_2026_8662_MOESM3_ESM.zip › Original Data/Fig. 3D/5-ECHS1.tif]

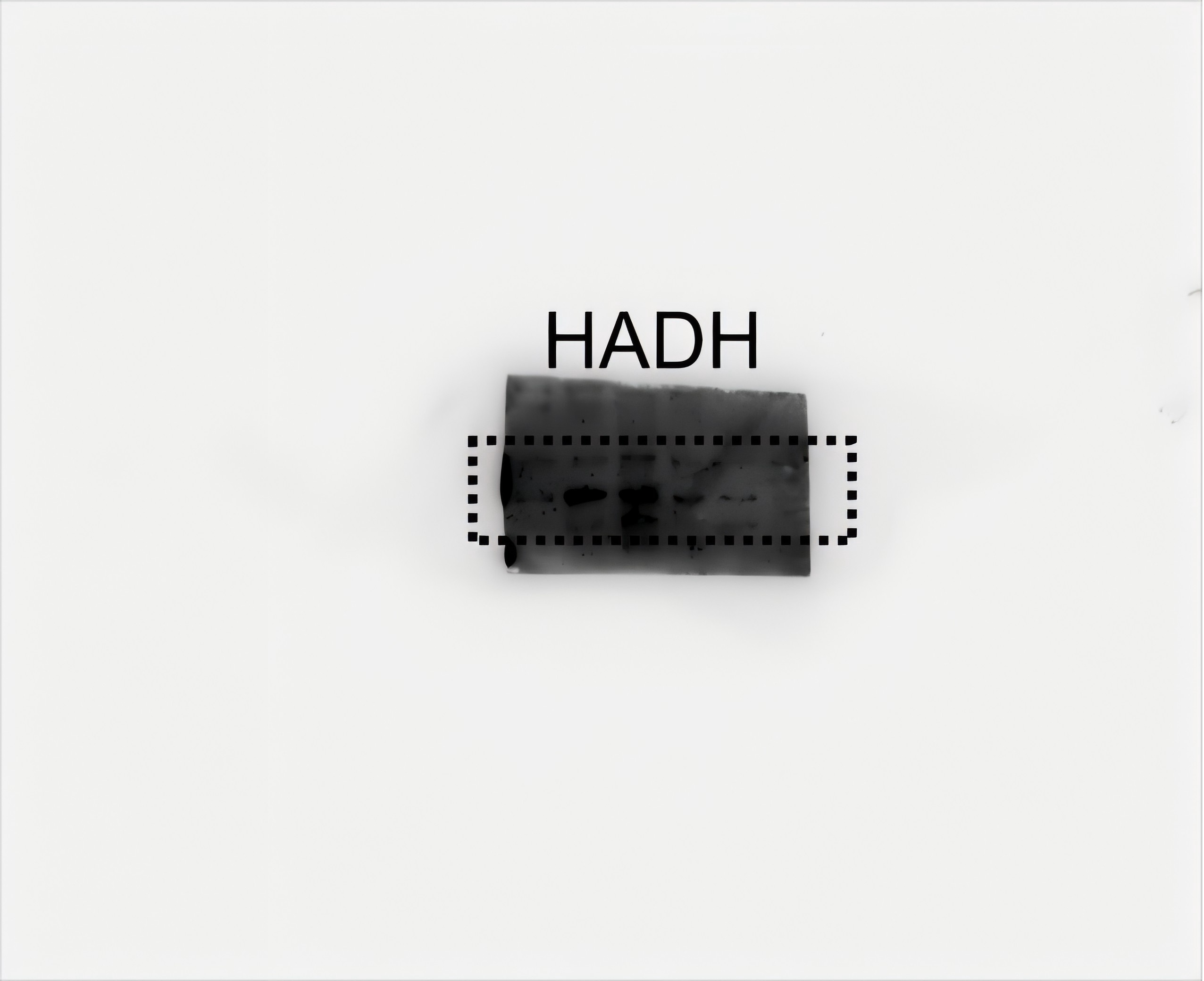

Supplement: Supplementary file 3 — Original Data [file 41419_2026_8662_MOESM3_ESM.zip › Original Data/Fig. 3D/6-HADH.tif]

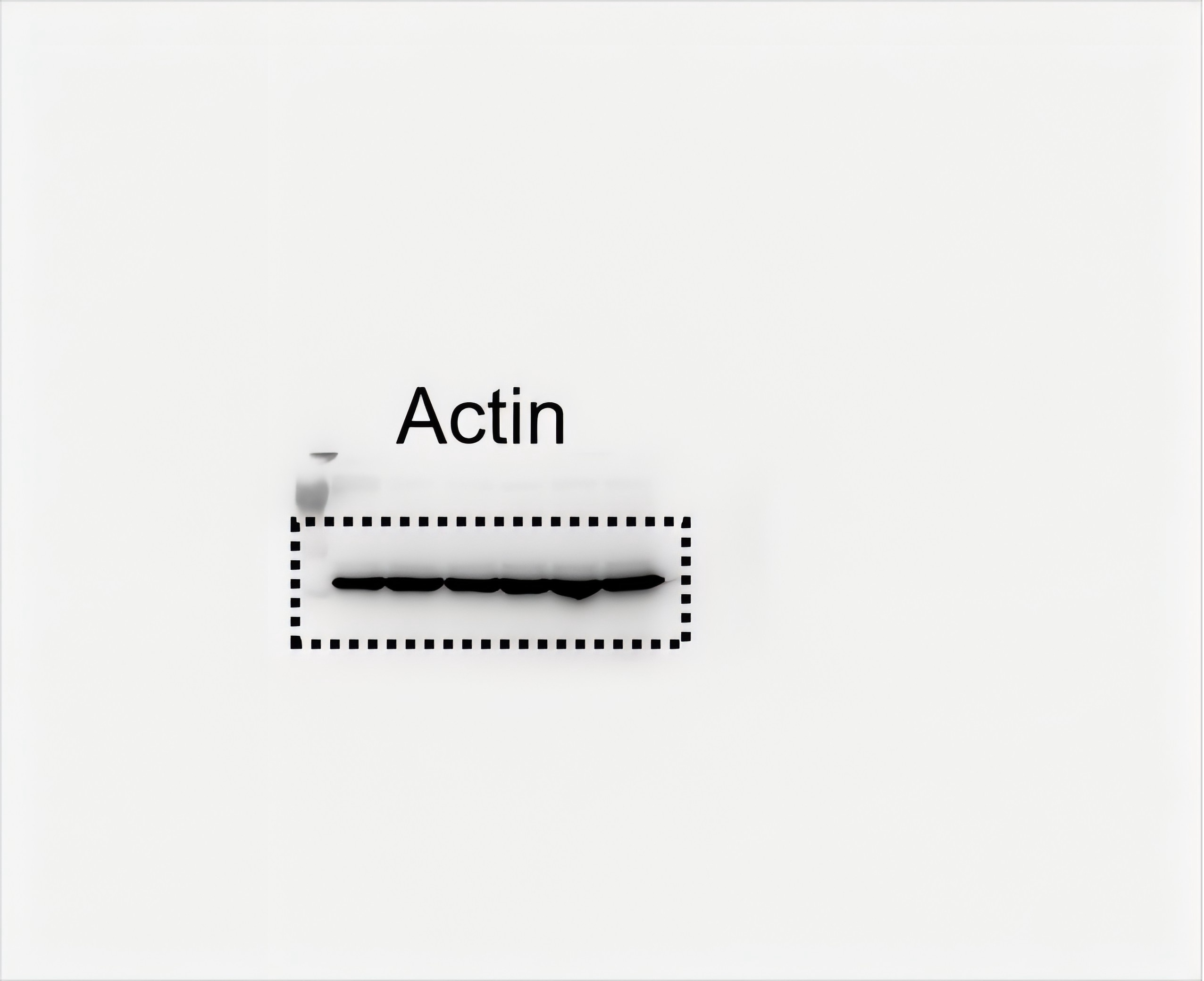

Supplement: Supplementary file 3 — Original Data [file 41419_2026_8662_MOESM3_ESM.zip › Original Data/Fig. 3D/7-Actin.tif]

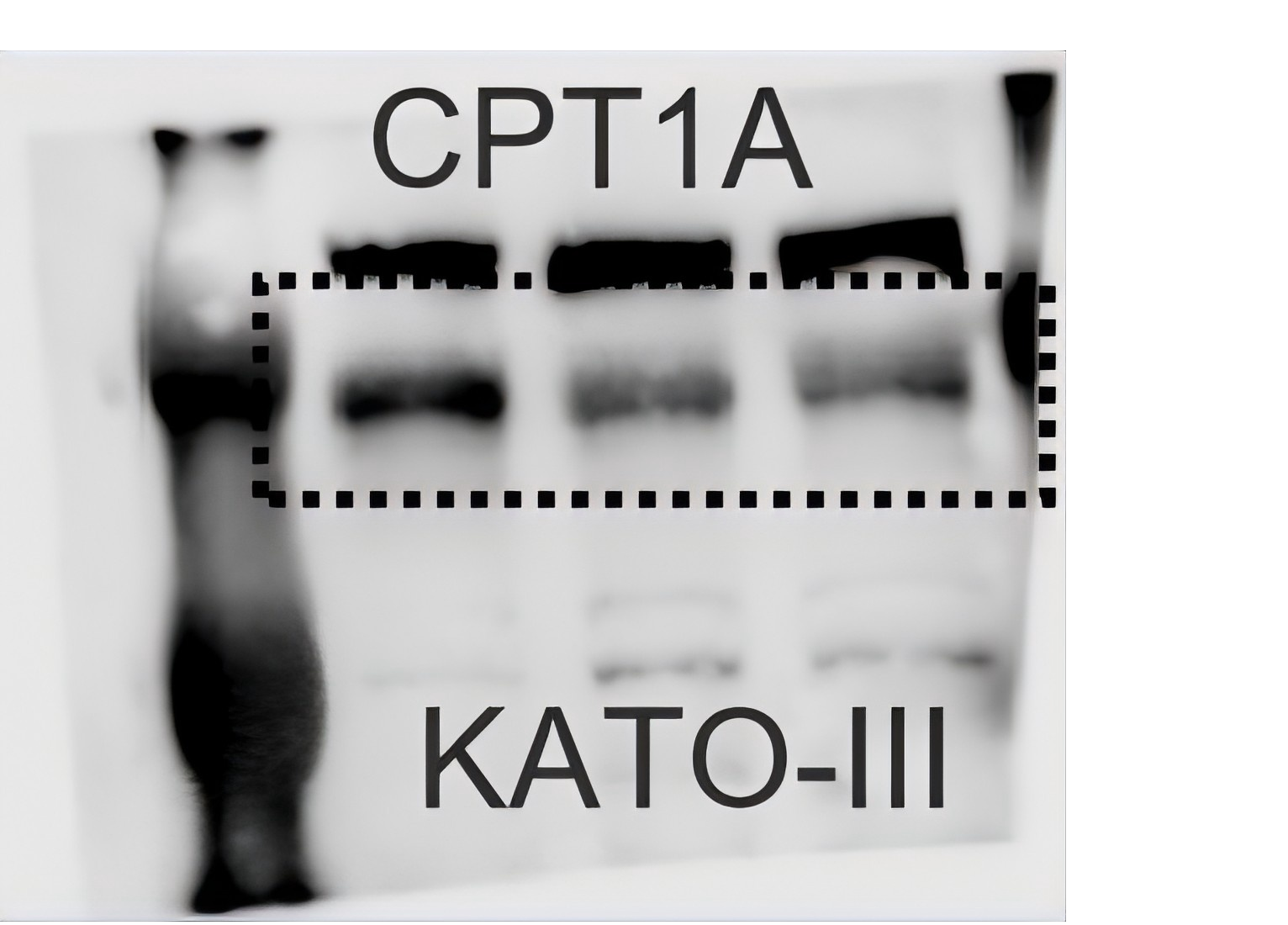

Supplement: Supplementary file 3 — Original Data [file 41419_2026_8662_MOESM3_ESM.zip › Original Data/Fig. 3E/1-CPT1A.tif]

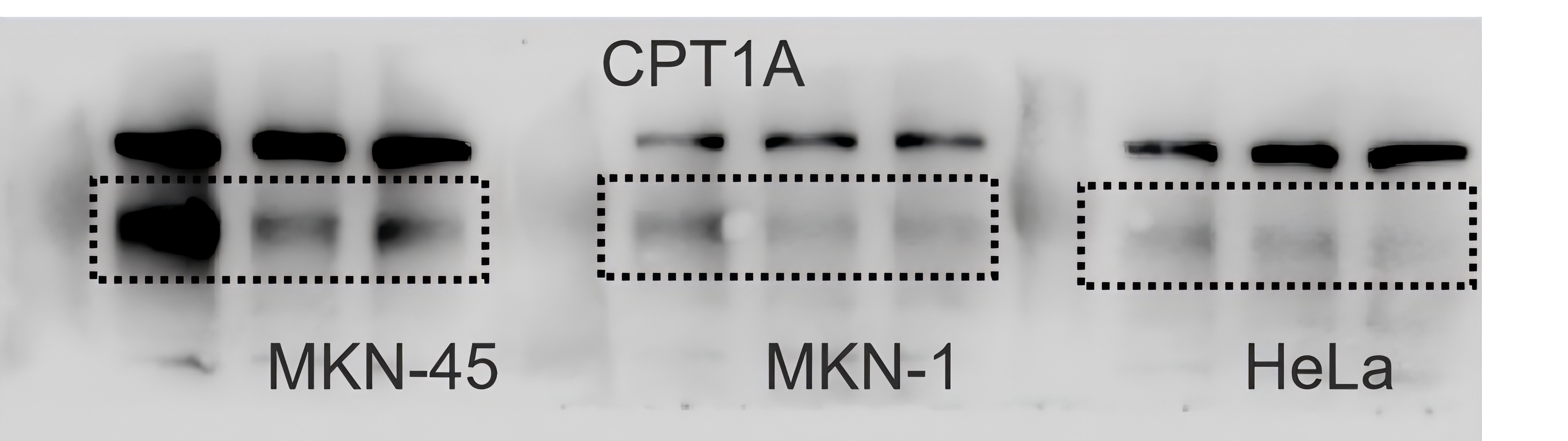

Supplement: Supplementary file 3 — Original Data [file 41419_2026_8662_MOESM3_ESM.zip › Original Data/Fig. 3E/2-CPT1A.tif]

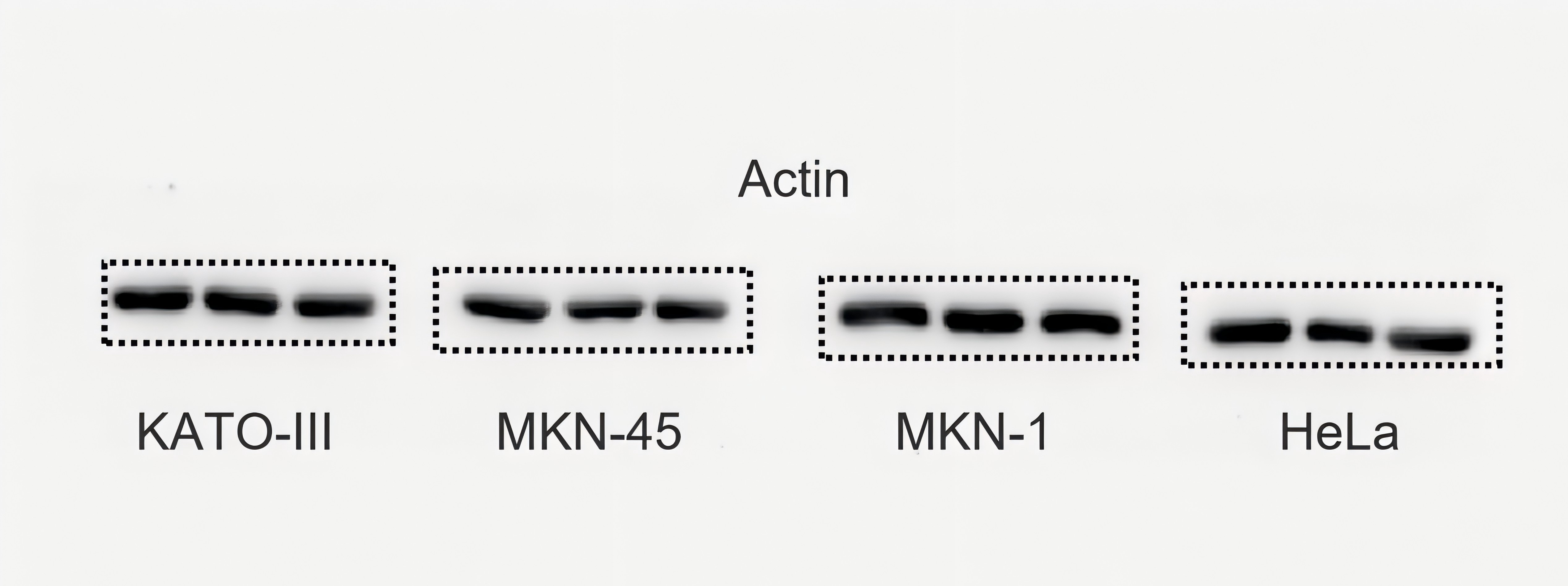

Supplement: Supplementary file 3 — Original Data [file 41419_2026_8662_MOESM3_ESM.zip › Original Data/Fig. 3E/3-Actin.tif]

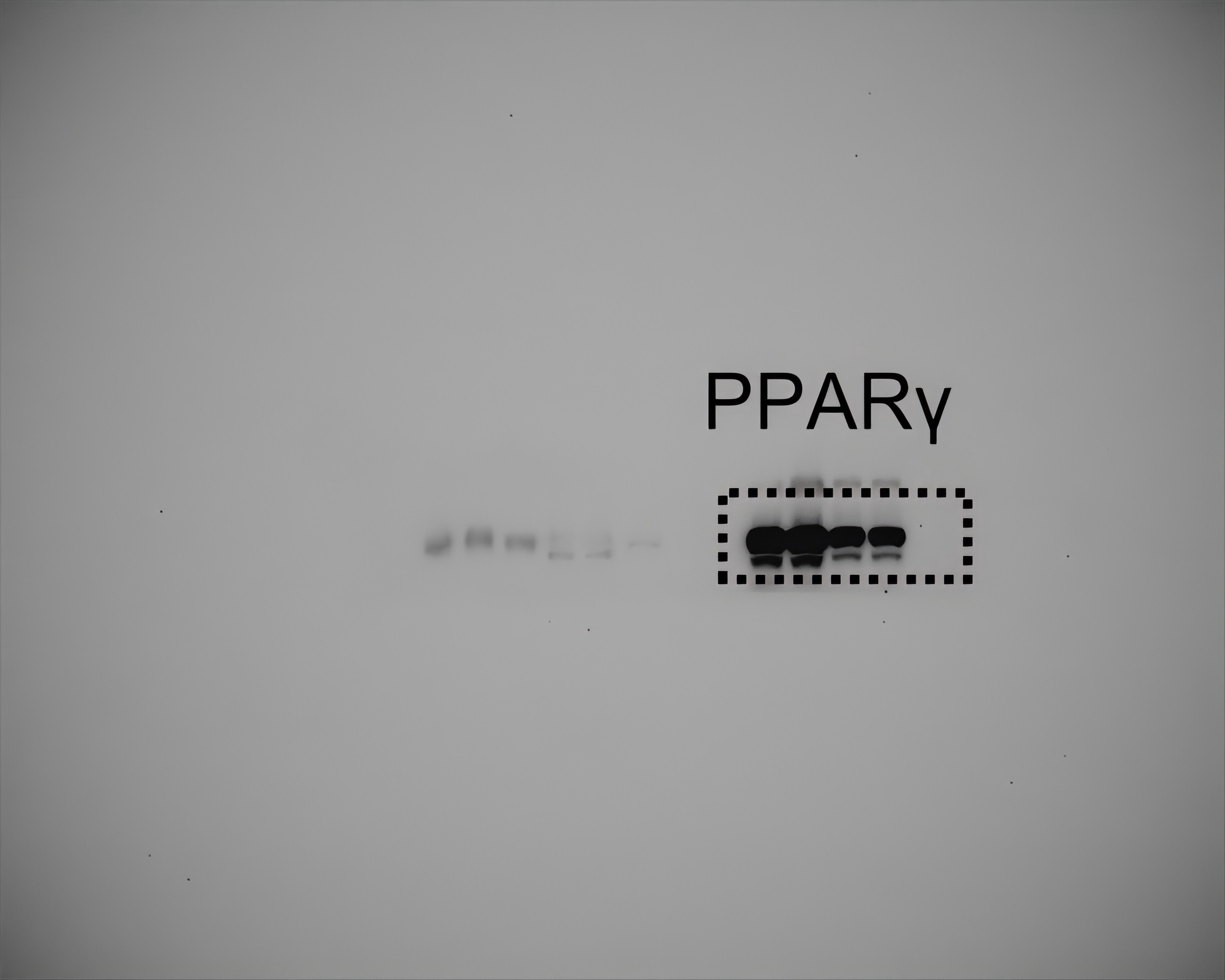

Supplement: Supplementary file 3 — Original Data [file 41419_2026_8662_MOESM3_ESM.zip › Original Data/Fig. 5C/1-PPARγ.tif]

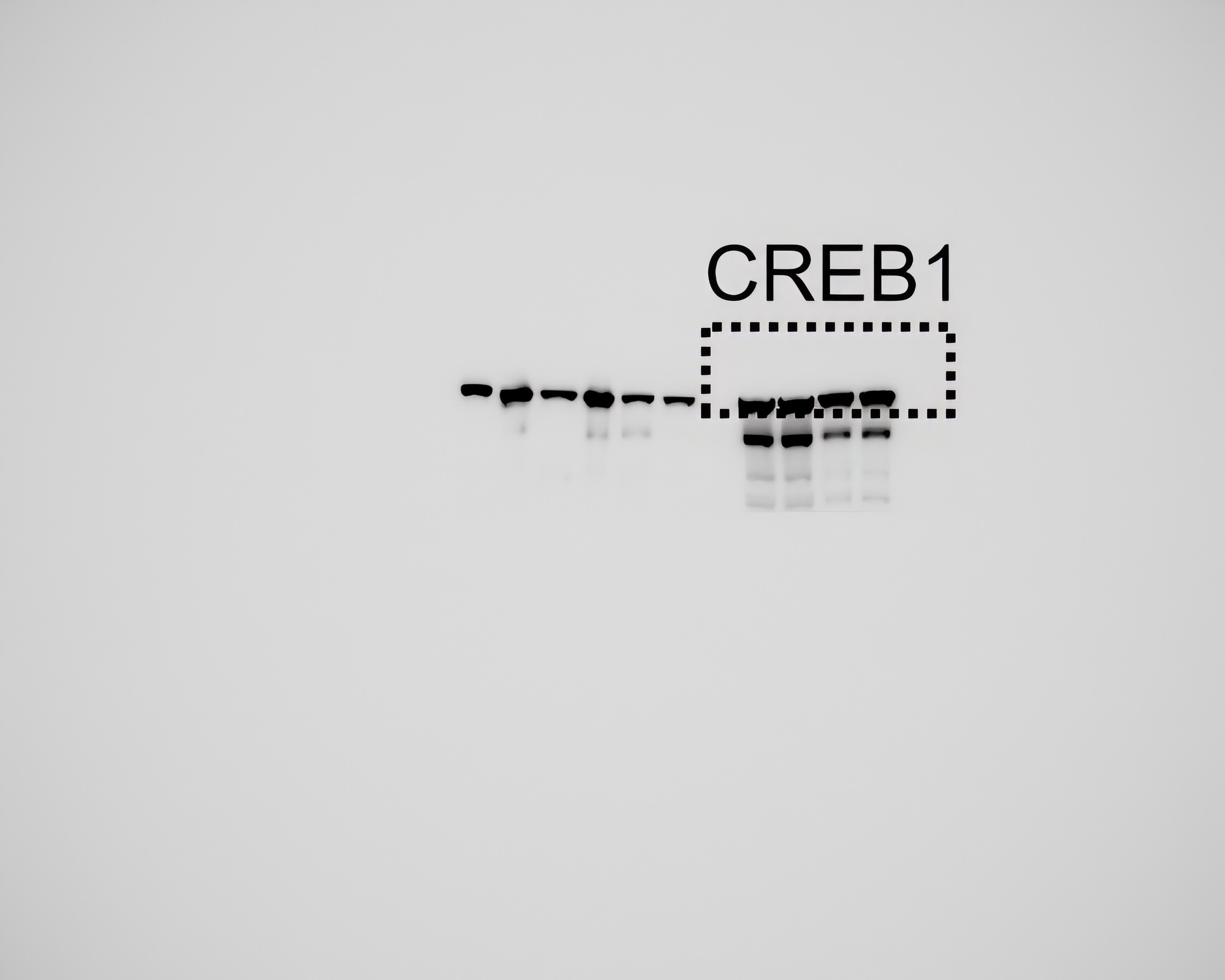

Supplement: Supplementary file 3 — Original Data [file 41419_2026_8662_MOESM3_ESM.zip › Original Data/Fig. 5C/2-CREB1.tif]

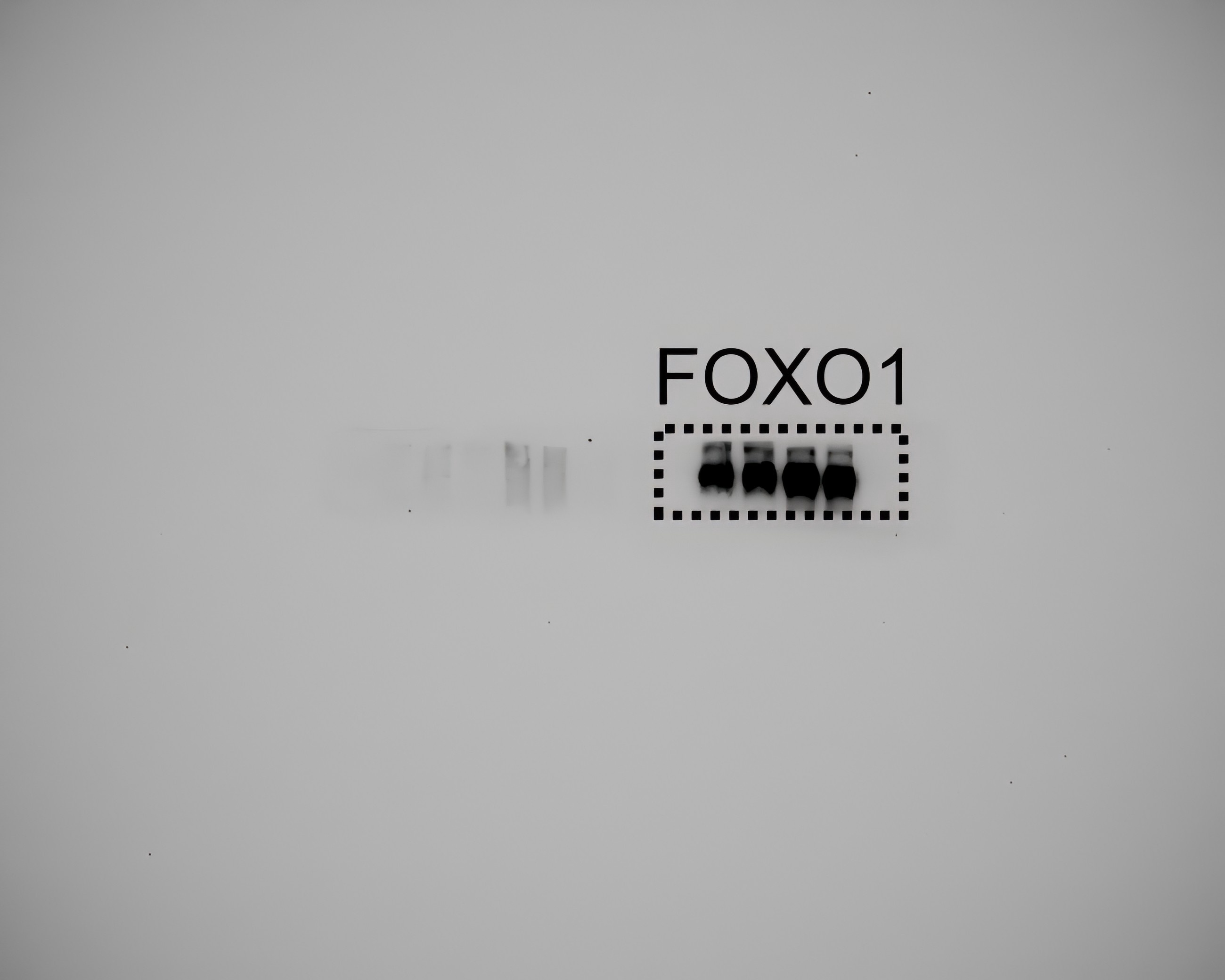

Supplement: Supplementary file 3 — Original Data [file 41419_2026_8662_MOESM3_ESM.zip › Original Data/Fig. 5C/3-FOXO1.tif]

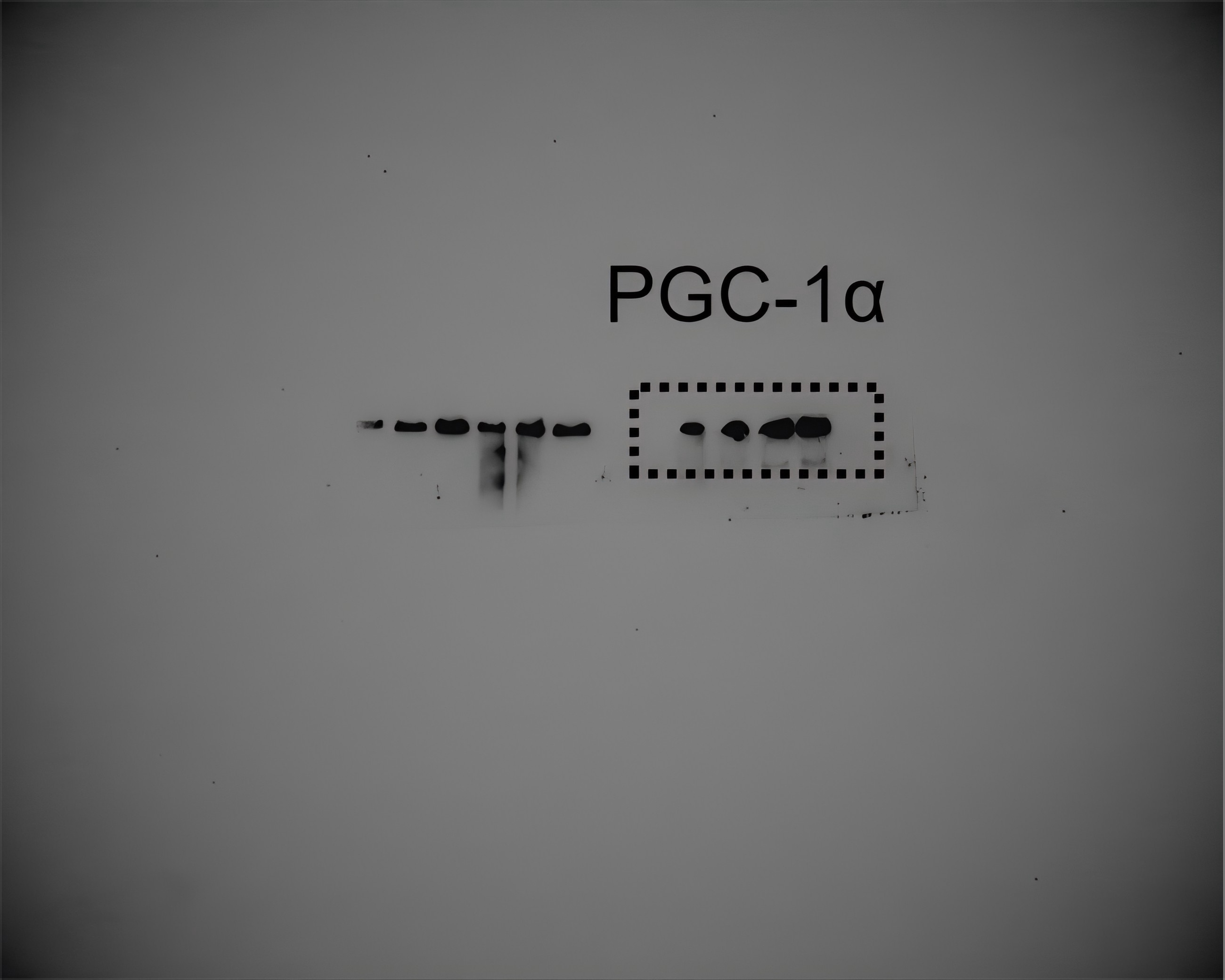

Supplement: Supplementary file 3 — Original Data [file 41419_2026_8662_MOESM3_ESM.zip › Original Data/Fig. 5C/4-PGC-1α.tif]

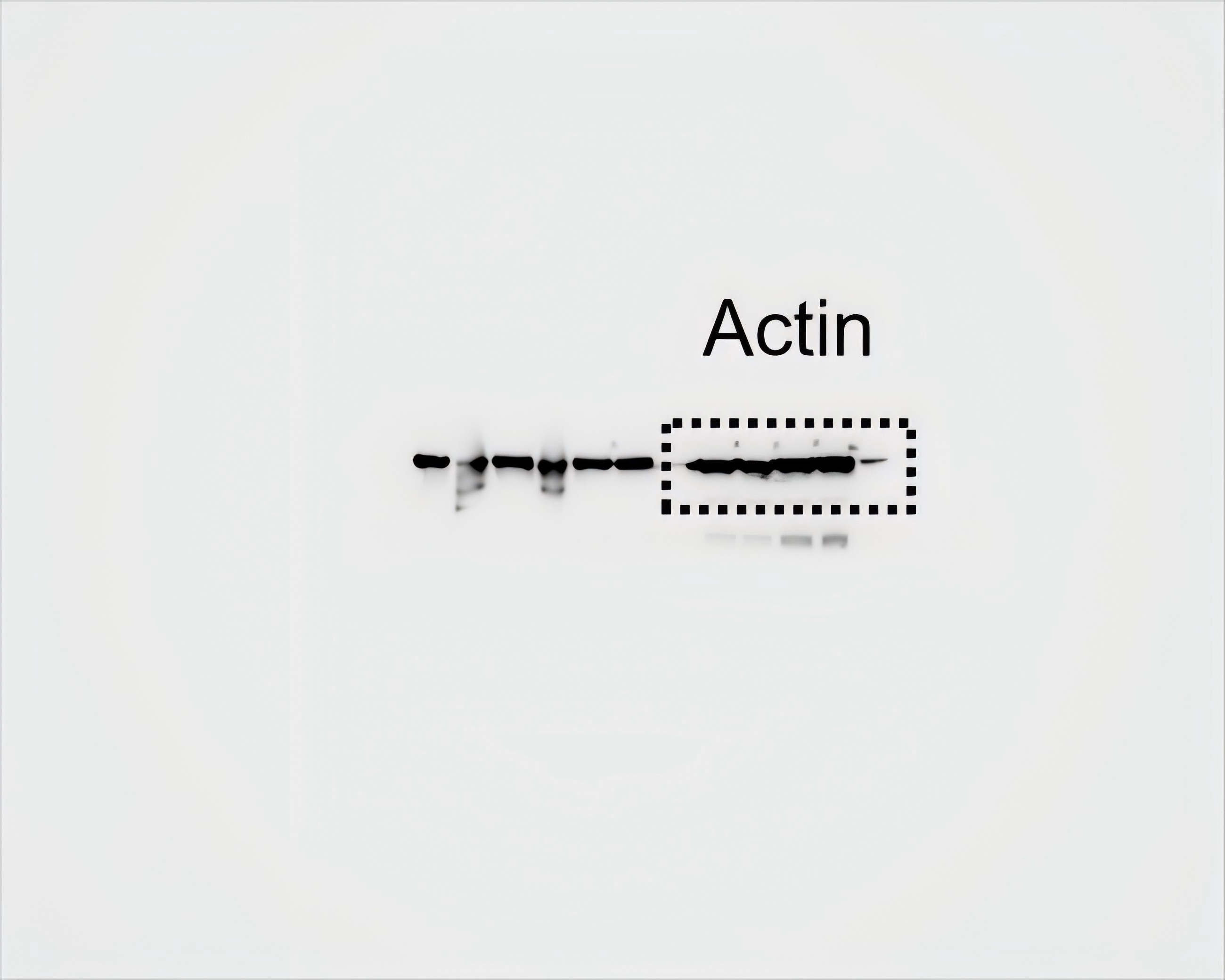

Supplement: Supplementary file 3 — Original Data [file 41419_2026_8662_MOESM3_ESM.zip › Original Data/Fig. 5C/5-Actin.tif]

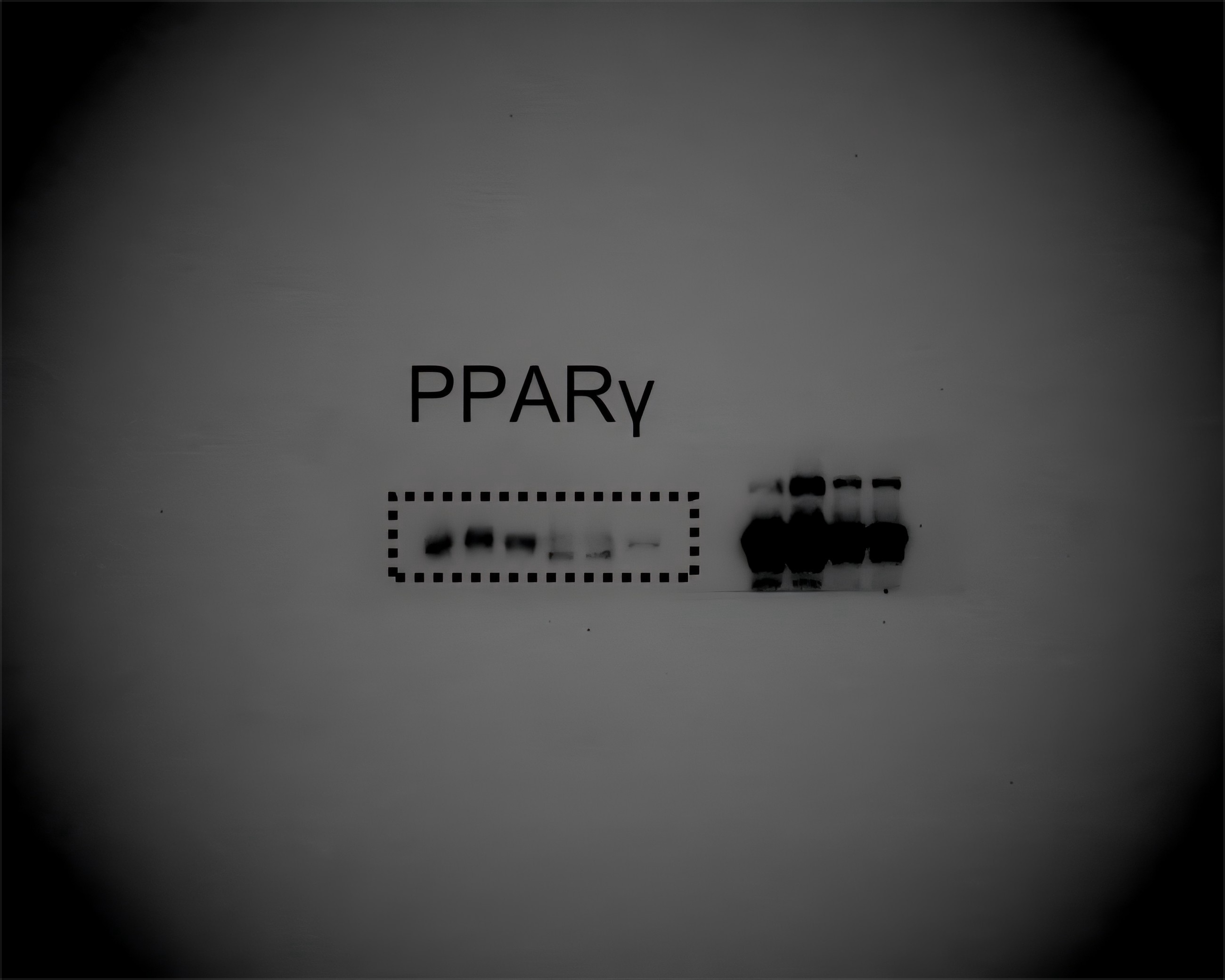

Supplement: Supplementary file 3 — Original Data [file 41419_2026_8662_MOESM3_ESM.zip › Original Data/Fig. 5D/1-PPARγ.tif]

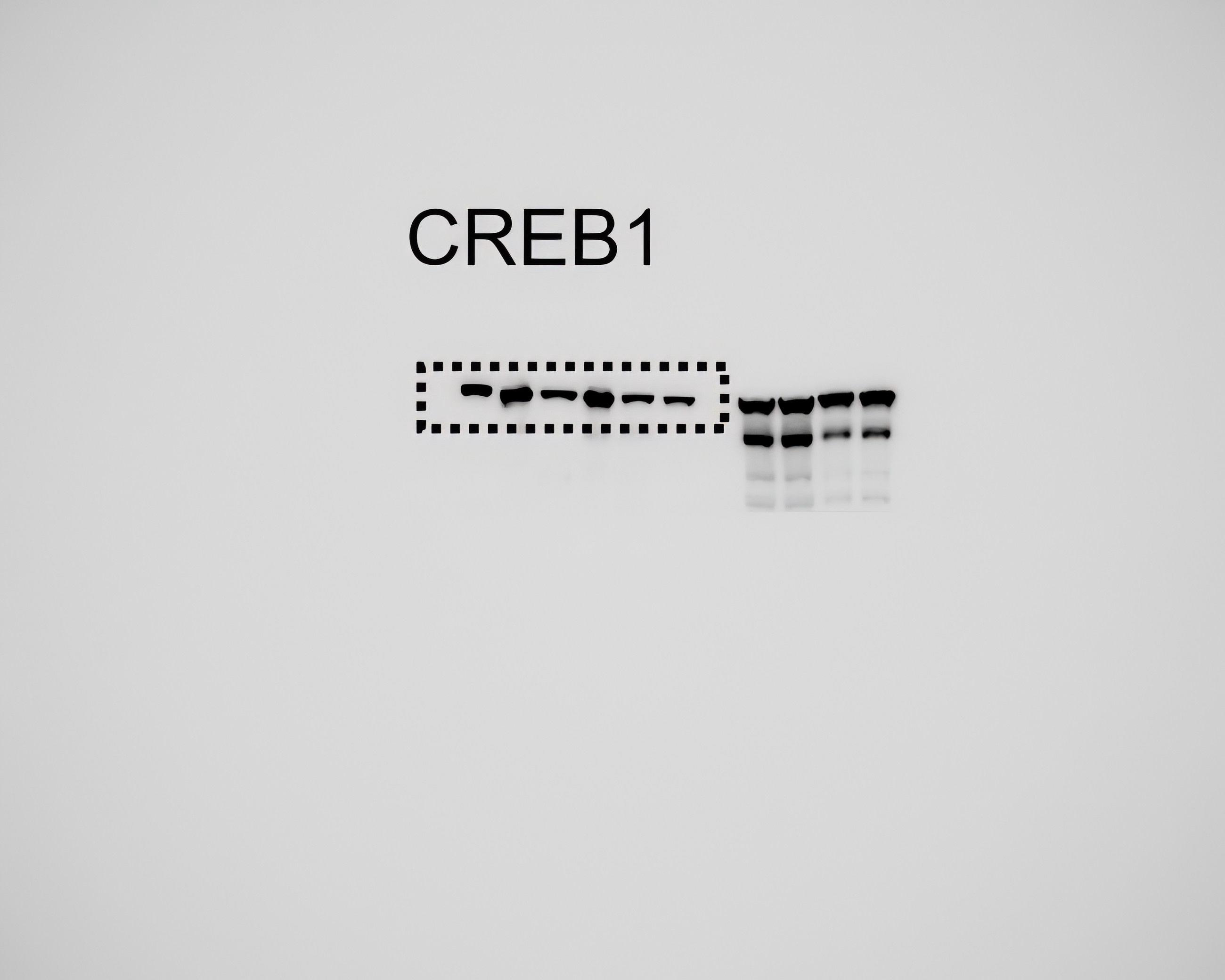

Supplement: Supplementary file 3 — Original Data [file 41419_2026_8662_MOESM3_ESM.zip › Original Data/Fig. 5D/2-CREB1.tif]

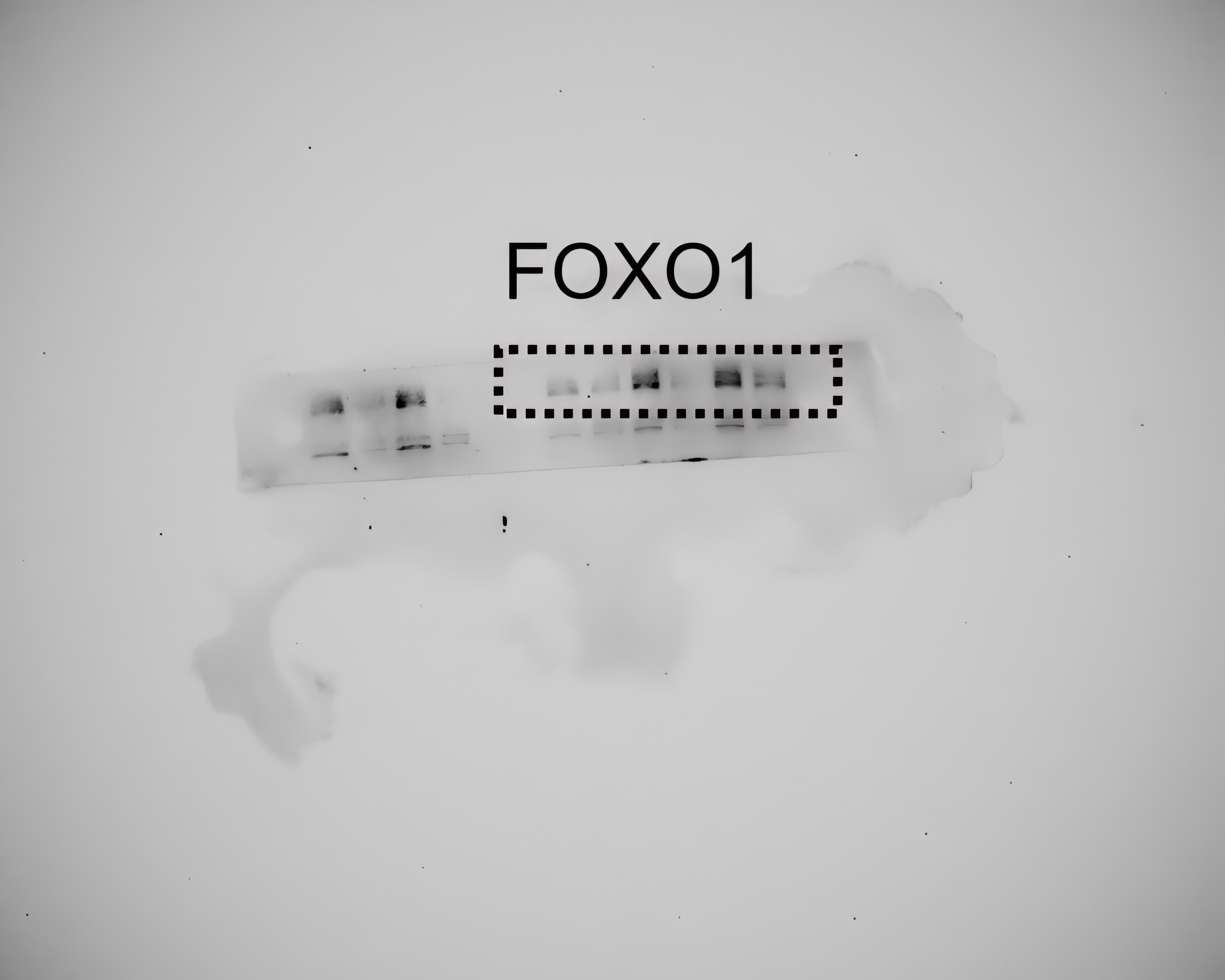

Supplement: Supplementary file 3 — Original Data [file 41419_2026_8662_MOESM3_ESM.zip › Original Data/Fig. 5D/3-FOXO1.tif]

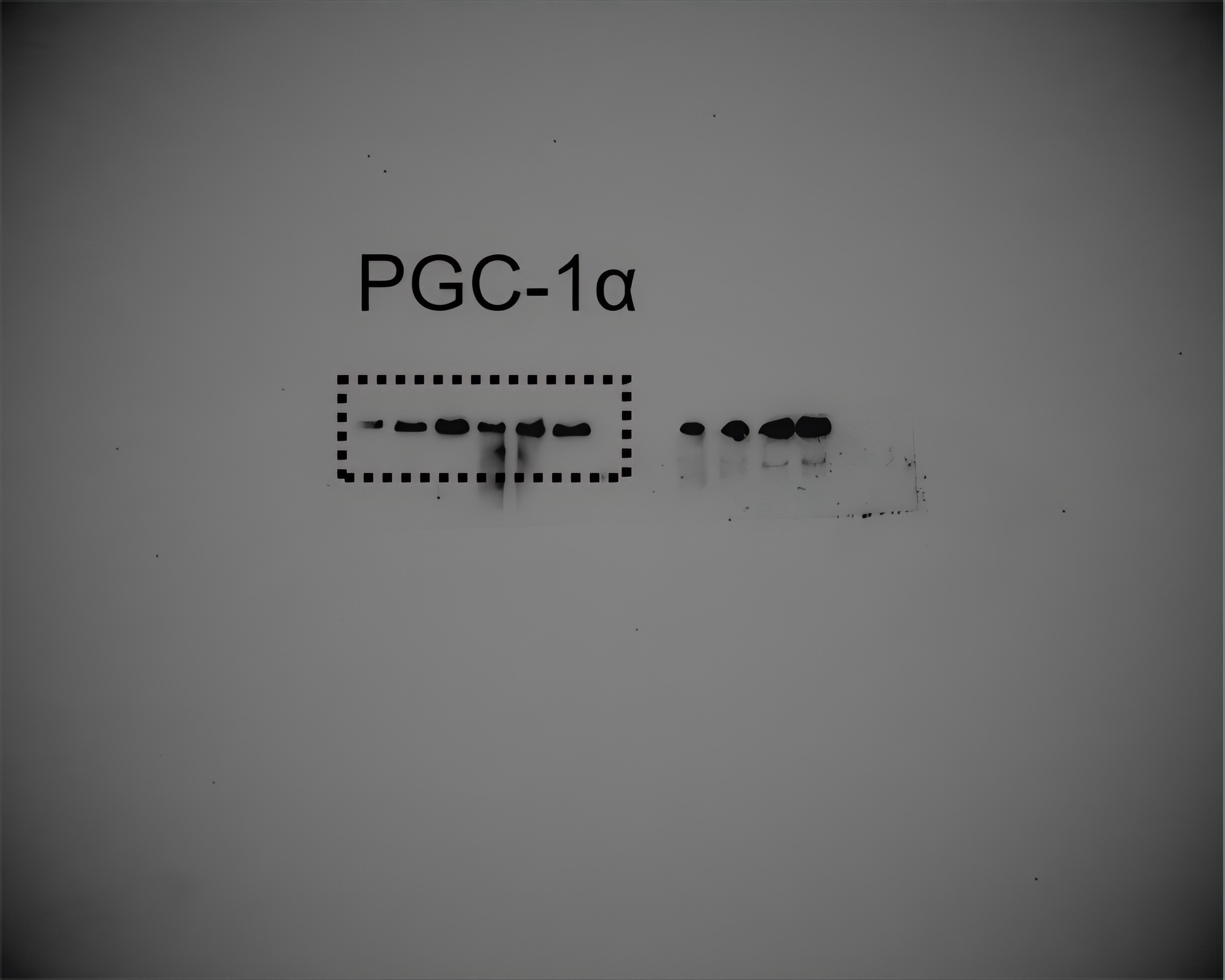

Supplement: Supplementary file 3 — Original Data [file 41419_2026_8662_MOESM3_ESM.zip › Original Data/Fig. 5D/4-PGC-1α.tif]

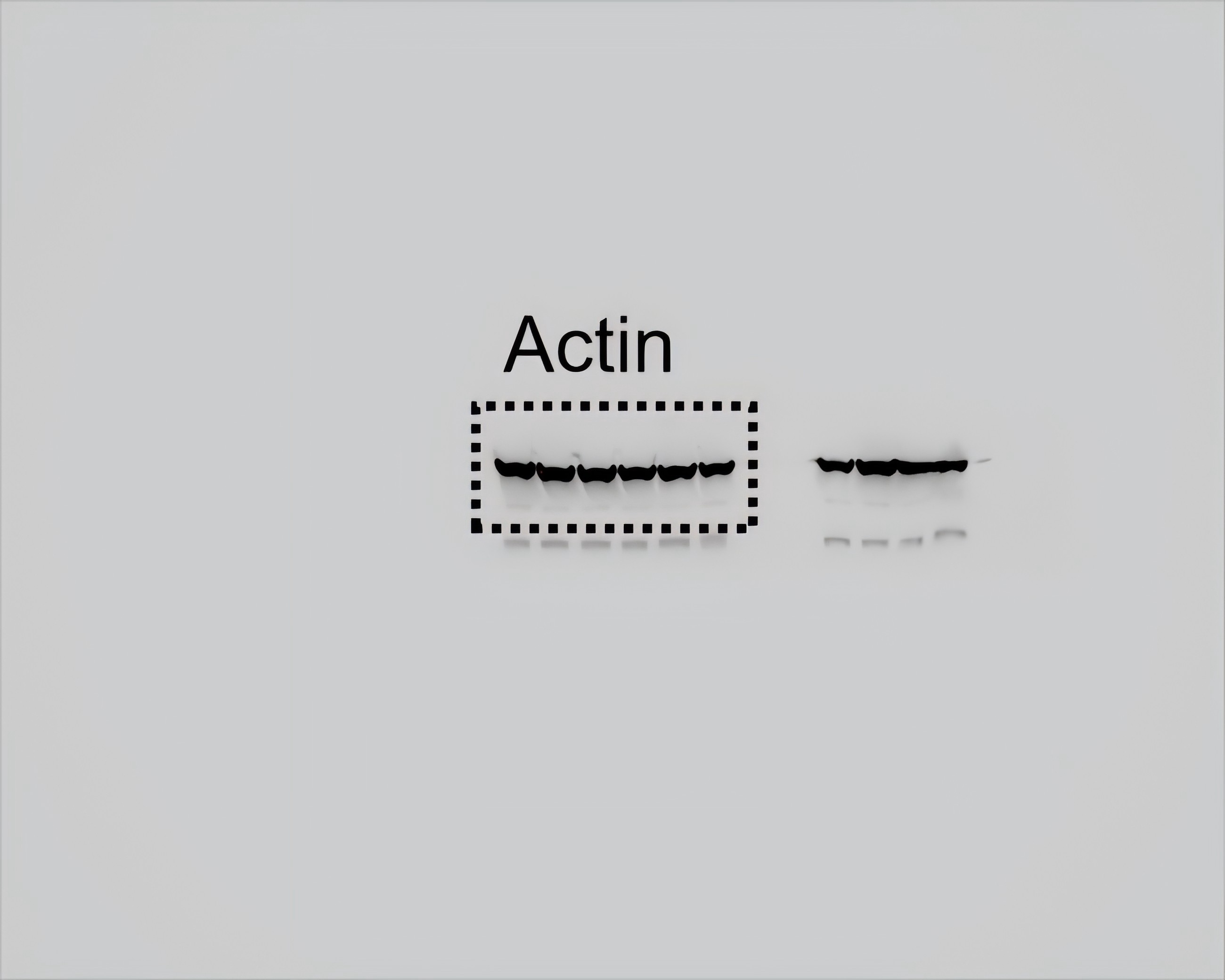

Supplement: Supplementary file 3 — Original Data [file 41419_2026_8662_MOESM3_ESM.zip › Original Data/Fig. 5D/5-Actin.tif]

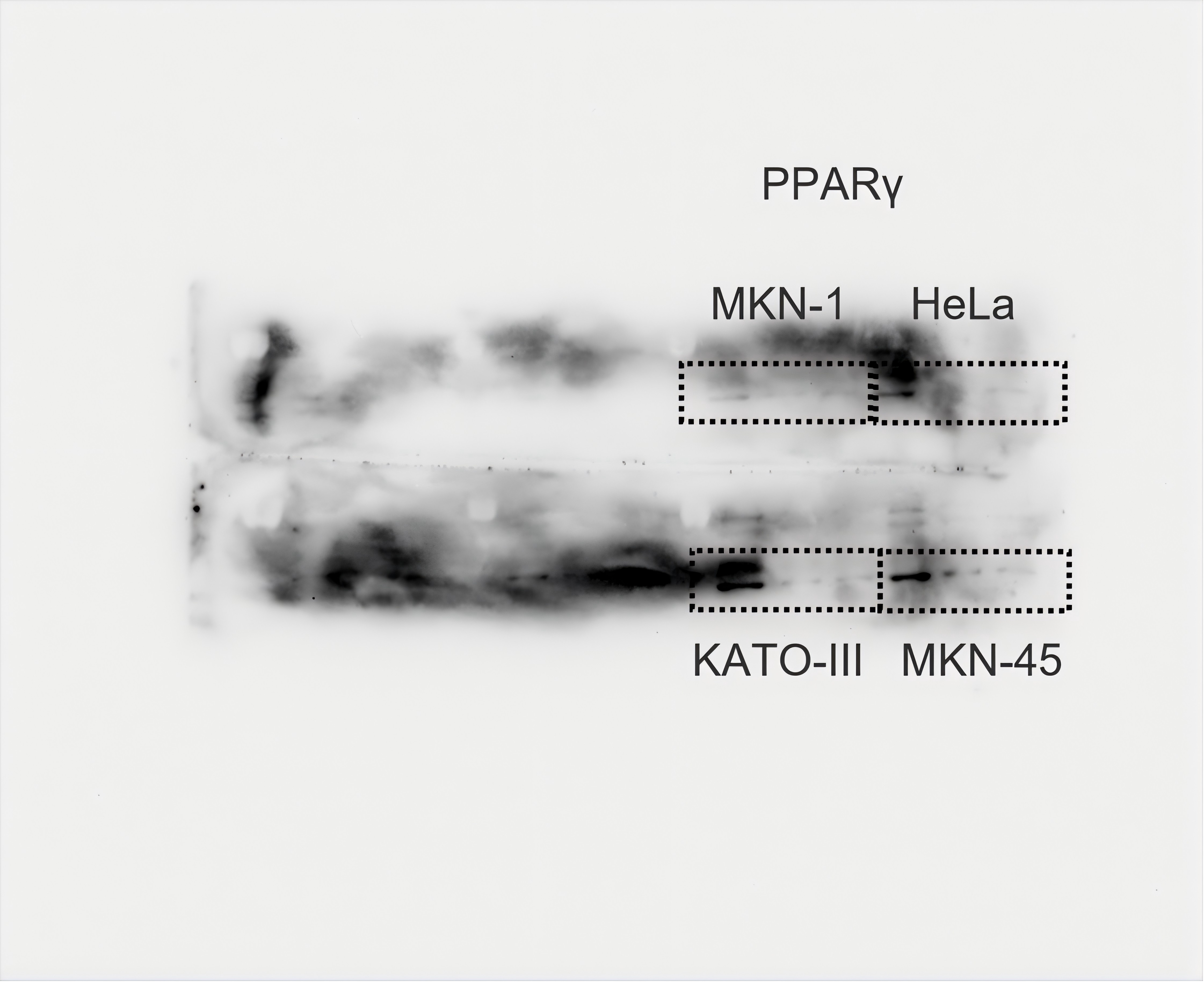

Supplement: Supplementary file 3 — Original Data [file 41419_2026_8662_MOESM3_ESM.zip › Original Data/Fig. 5G/1-PPARγ.tif]

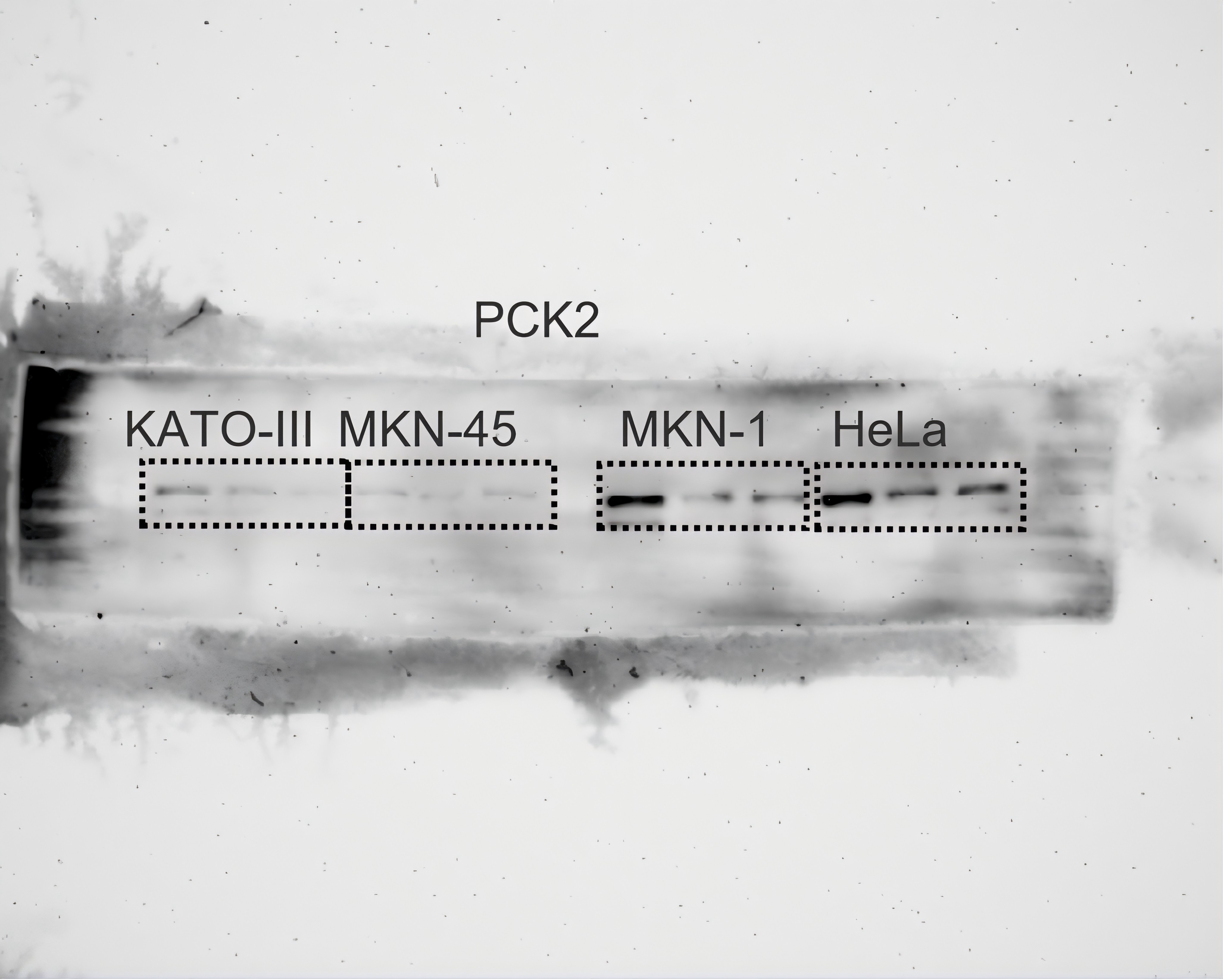

Supplement: Supplementary file 3 — Original Data [file 41419_2026_8662_MOESM3_ESM.zip › Original Data/Fig. 5G/2-PCK2.tif]

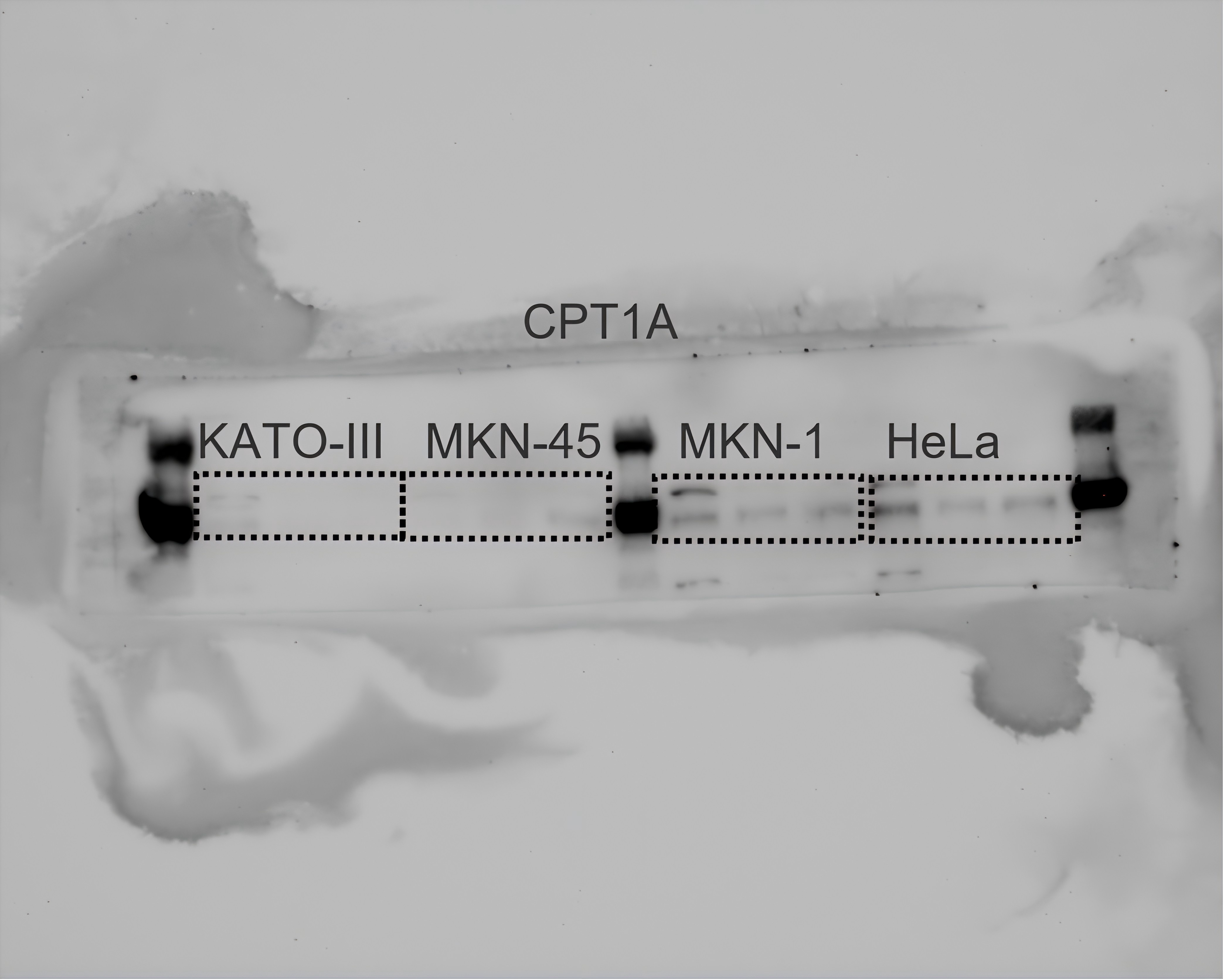

Supplement: Supplementary file 3 — Original Data [file 41419_2026_8662_MOESM3_ESM.zip › Original Data/Fig. 5G/3-CPT1A.tif]

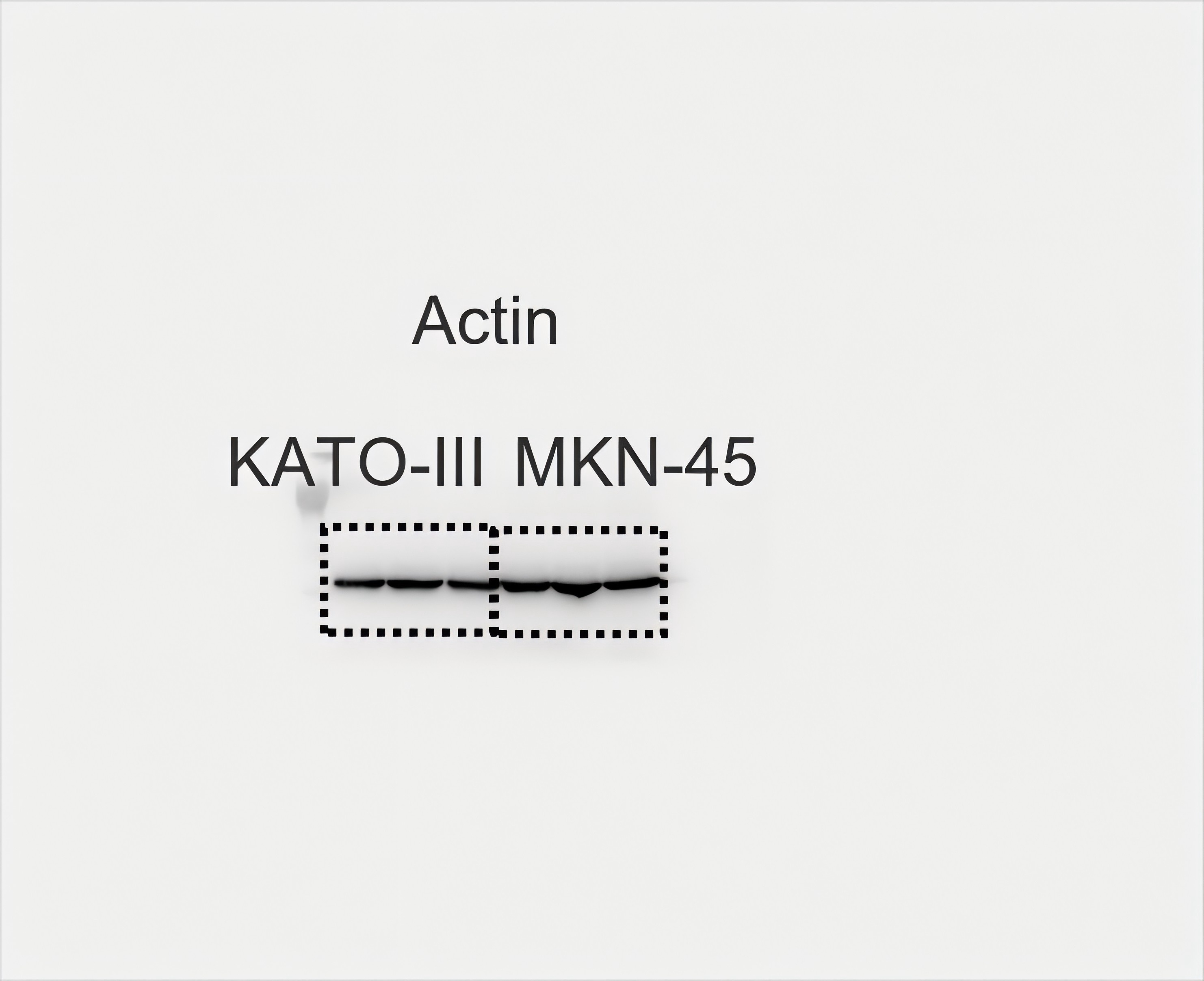

Supplement: Supplementary file 3 — Original Data [file 41419_2026_8662_MOESM3_ESM.zip › Original Data/Fig. 5G/4-Actin.tif]

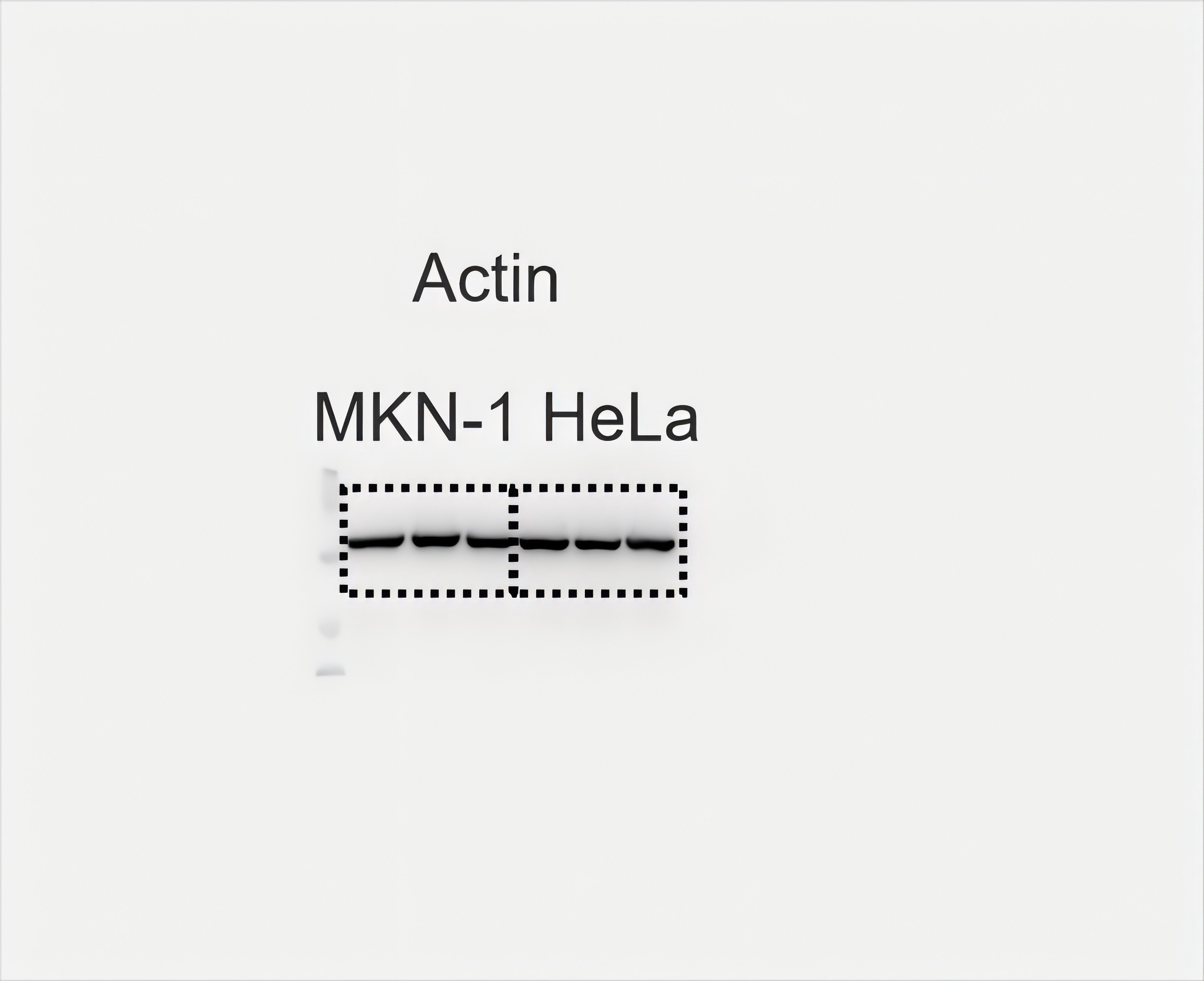

Supplement: Supplementary file 3 — Original Data [file 41419_2026_8662_MOESM3_ESM.zip › Original Data/Fig. 5G/5-Actin.tif]

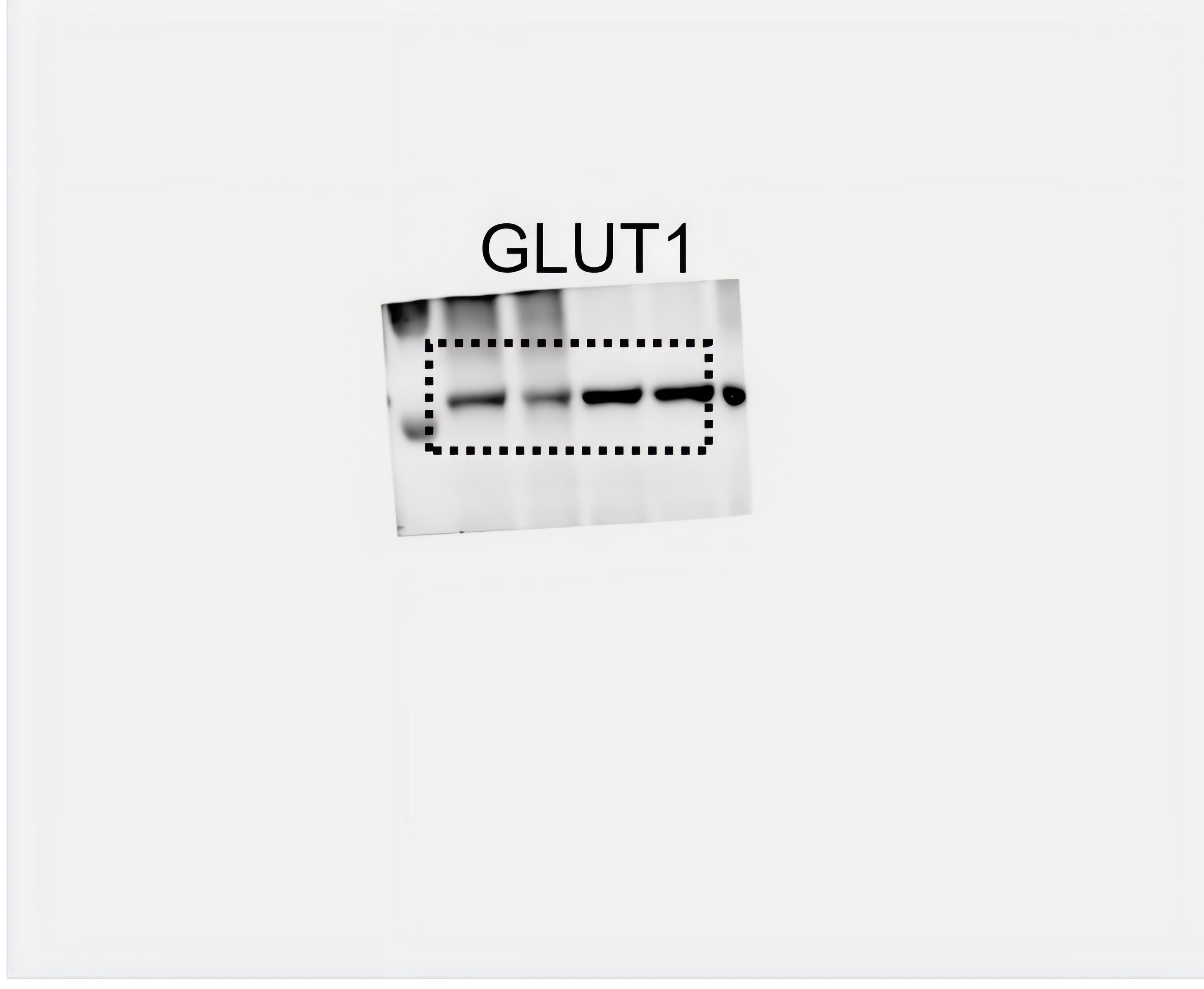

Supplement: Supplementary file 3 — Original Data [file 41419_2026_8662_MOESM3_ESM.zip › Original Data/Fig. S1/1-GLUT1.tif]

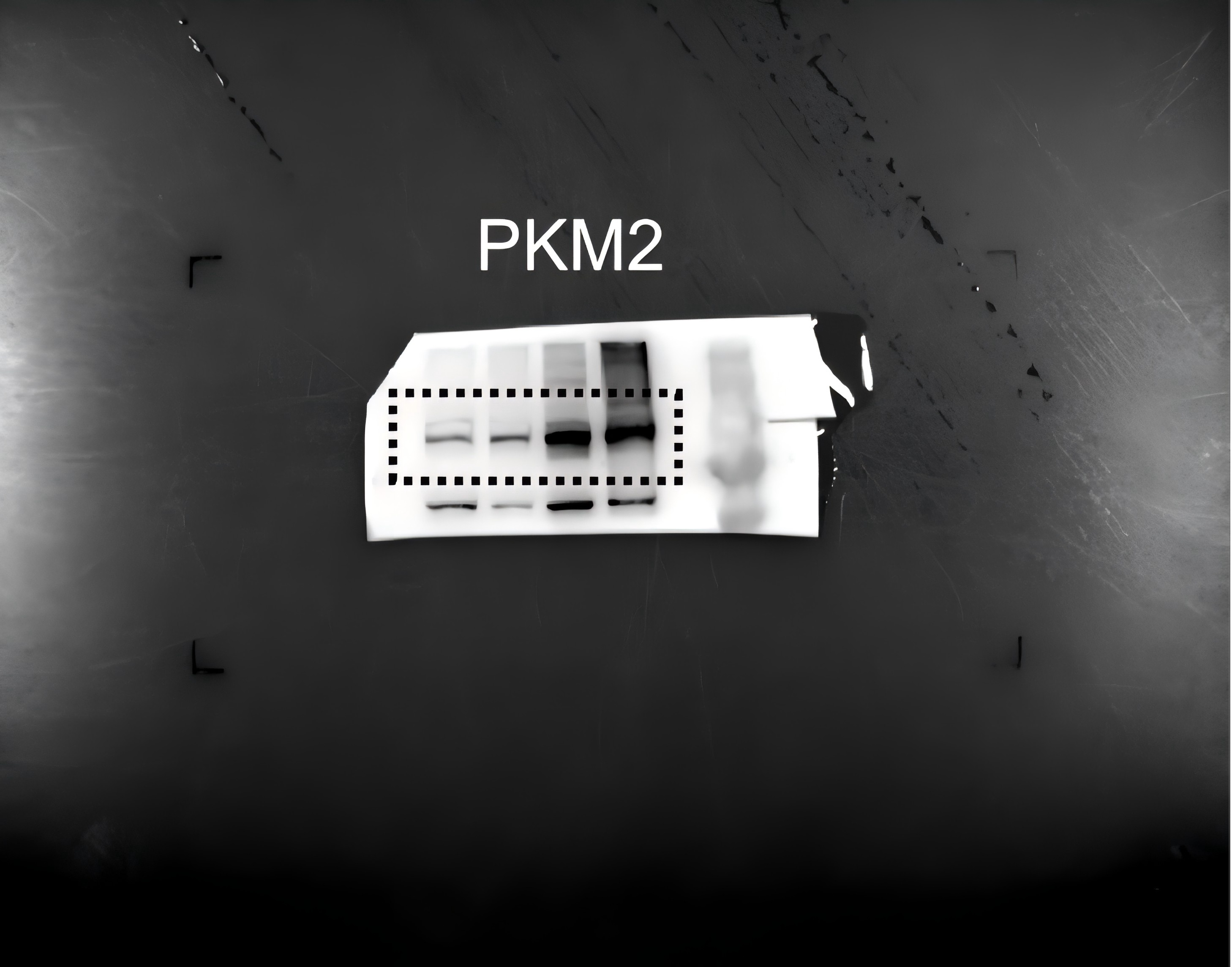

Supplement: Supplementary file 3 — Original Data [file 41419_2026_8662_MOESM3_ESM.zip › Original Data/Fig. S1/10-PKM2.tif]

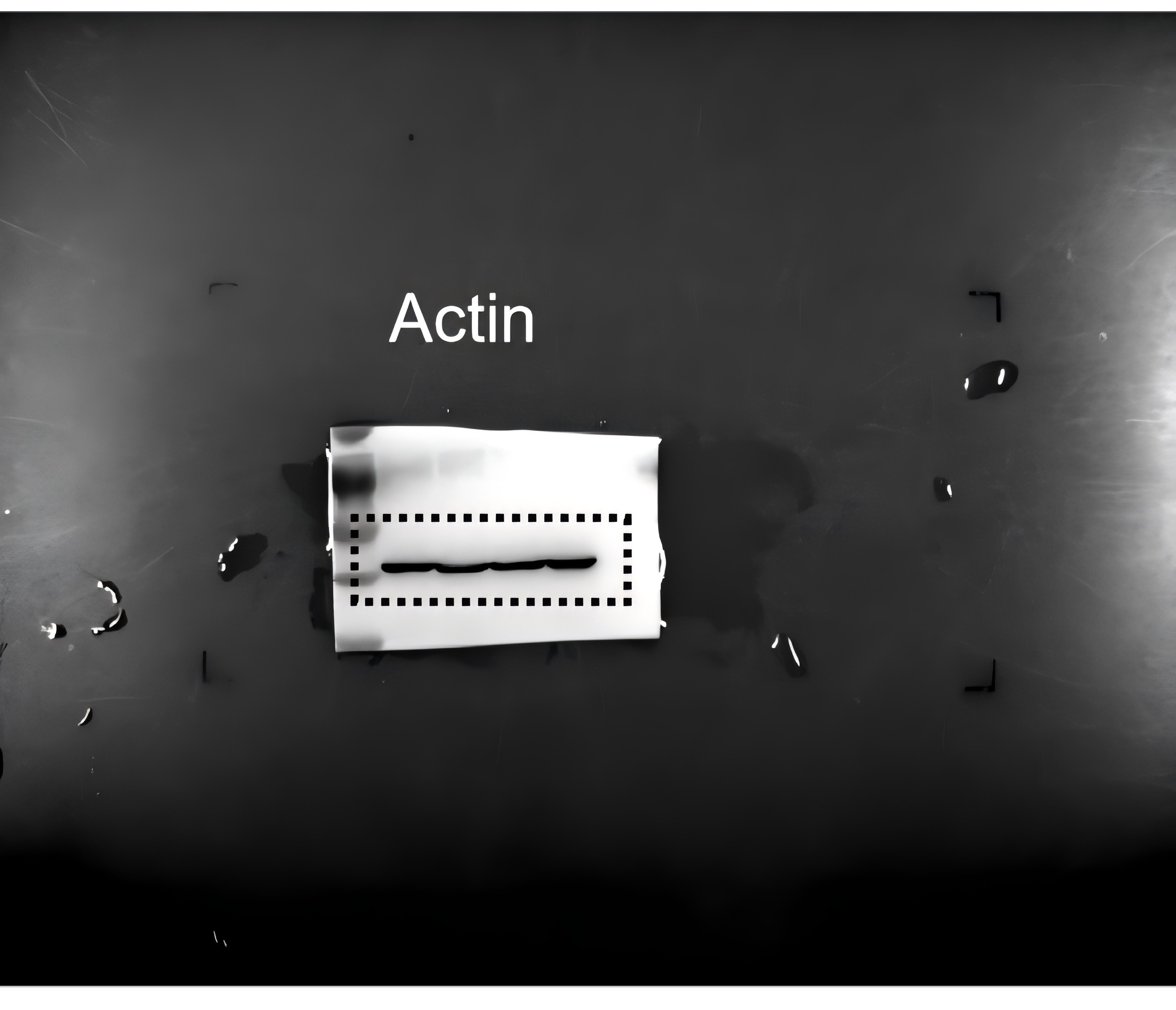

Supplement: Supplementary file 3 — Original Data [file 41419_2026_8662_MOESM3_ESM.zip › Original Data/Fig. S1/11-Actin.tif]

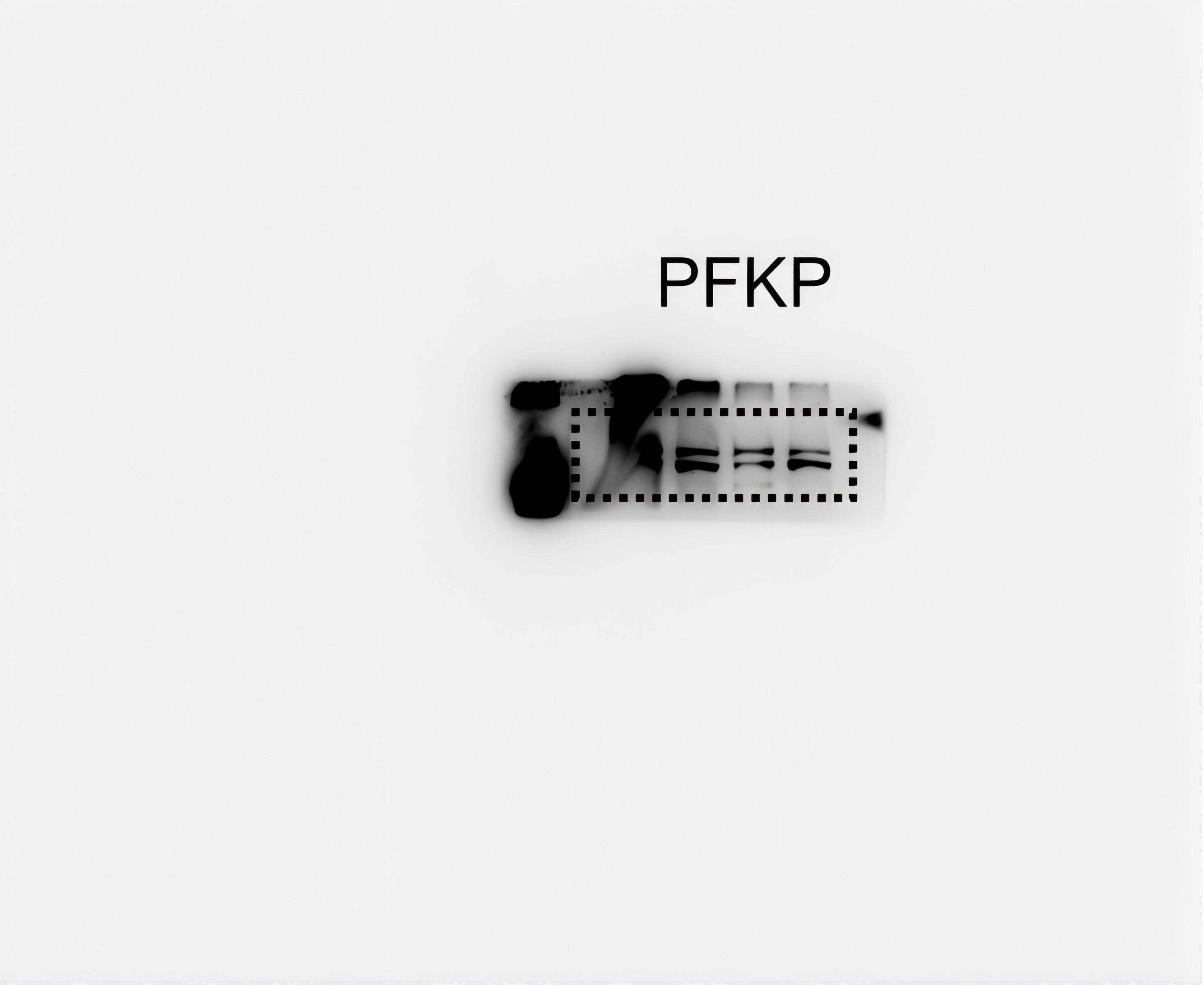

Supplement: Supplementary file 3 — Original Data [file 41419_2026_8662_MOESM3_ESM.zip › Original Data/Fig. S1/2-PFKP.tif]

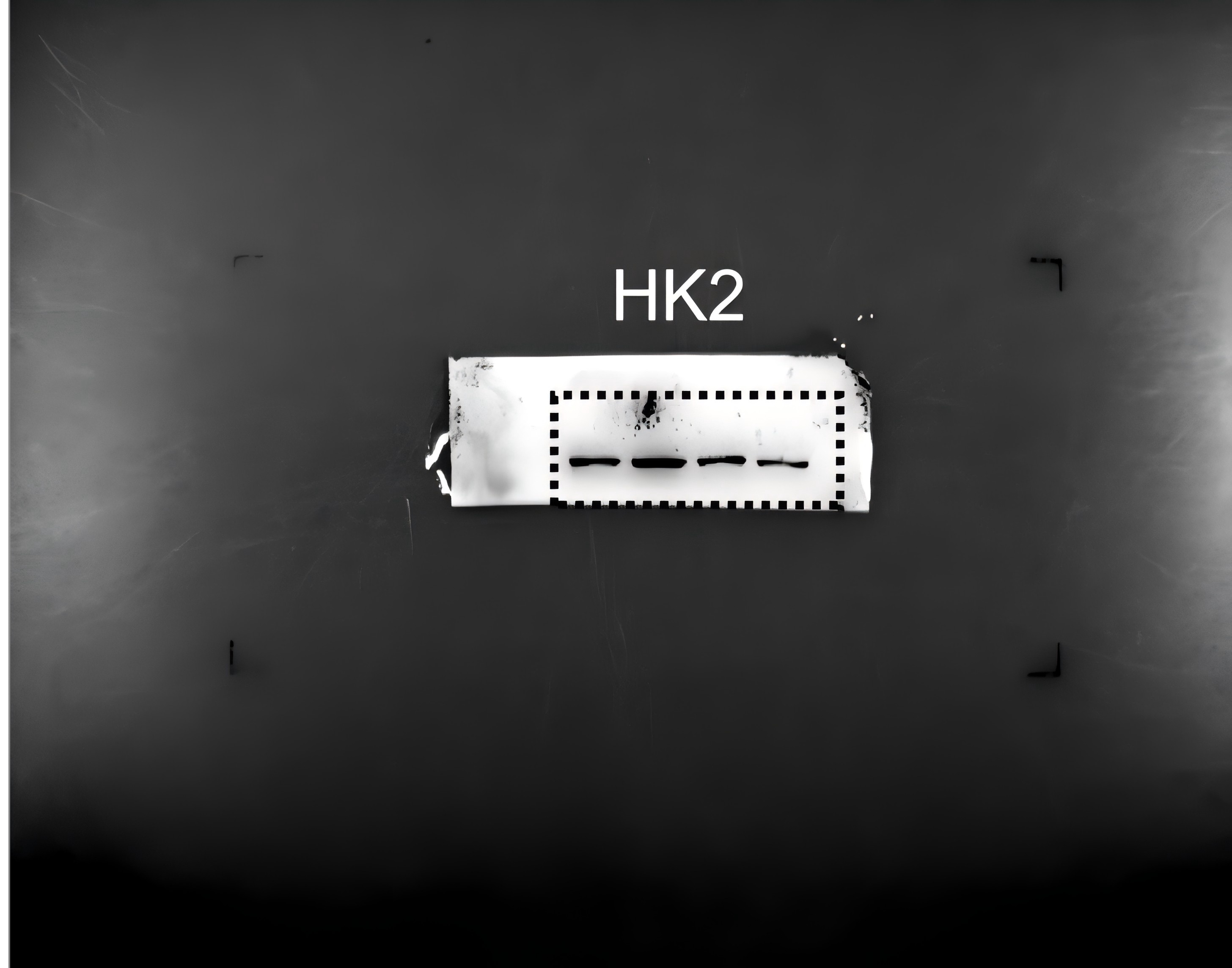

Supplement: Supplementary file 3 — Original Data [file 41419_2026_8662_MOESM3_ESM.zip › Original Data/Fig. S1/3-HK2.tif]

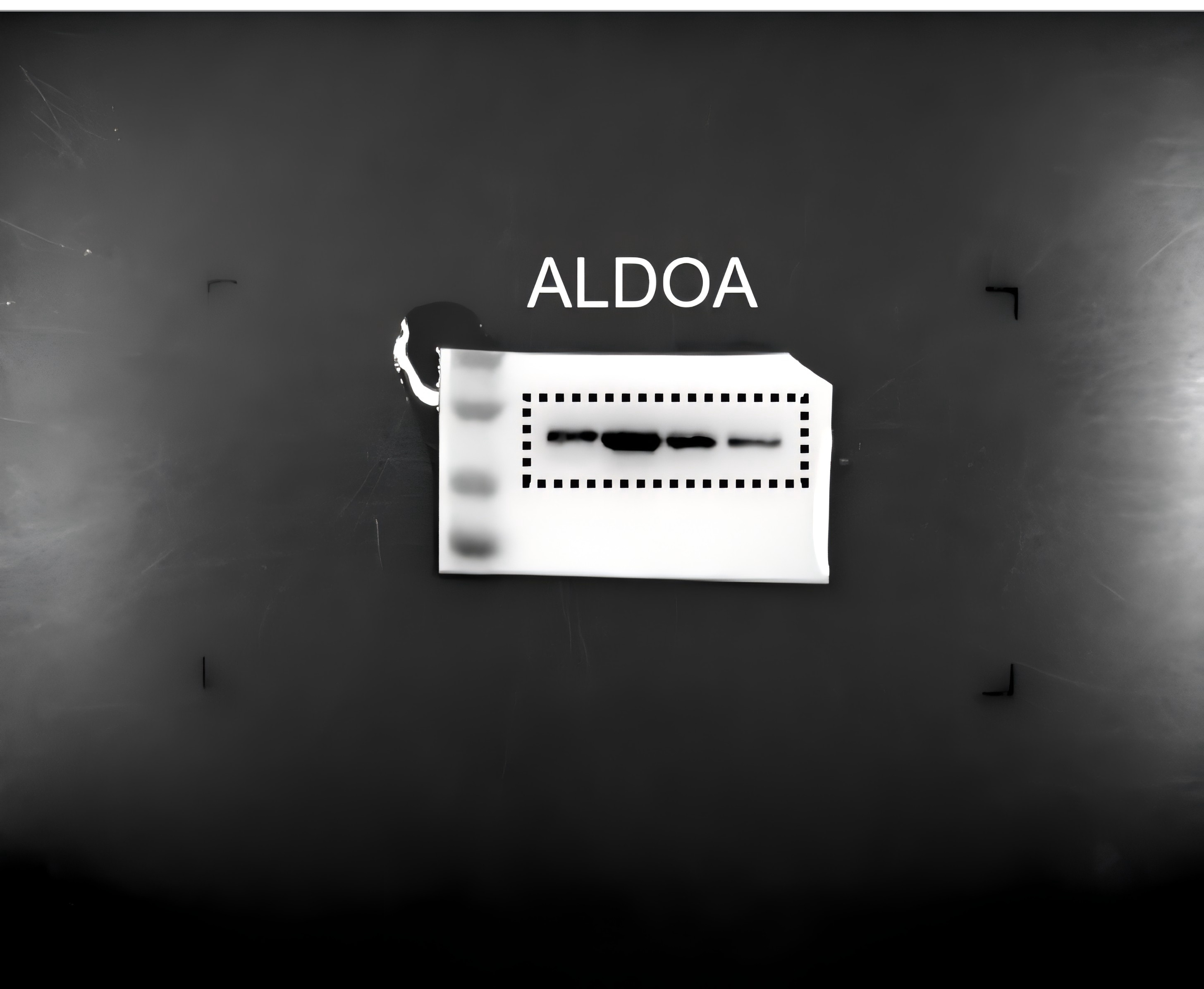

Supplement: Supplementary file 3 — Original Data [file 41419_2026_8662_MOESM3_ESM.zip › Original Data/Fig. S1/4-ALDOA.tif]

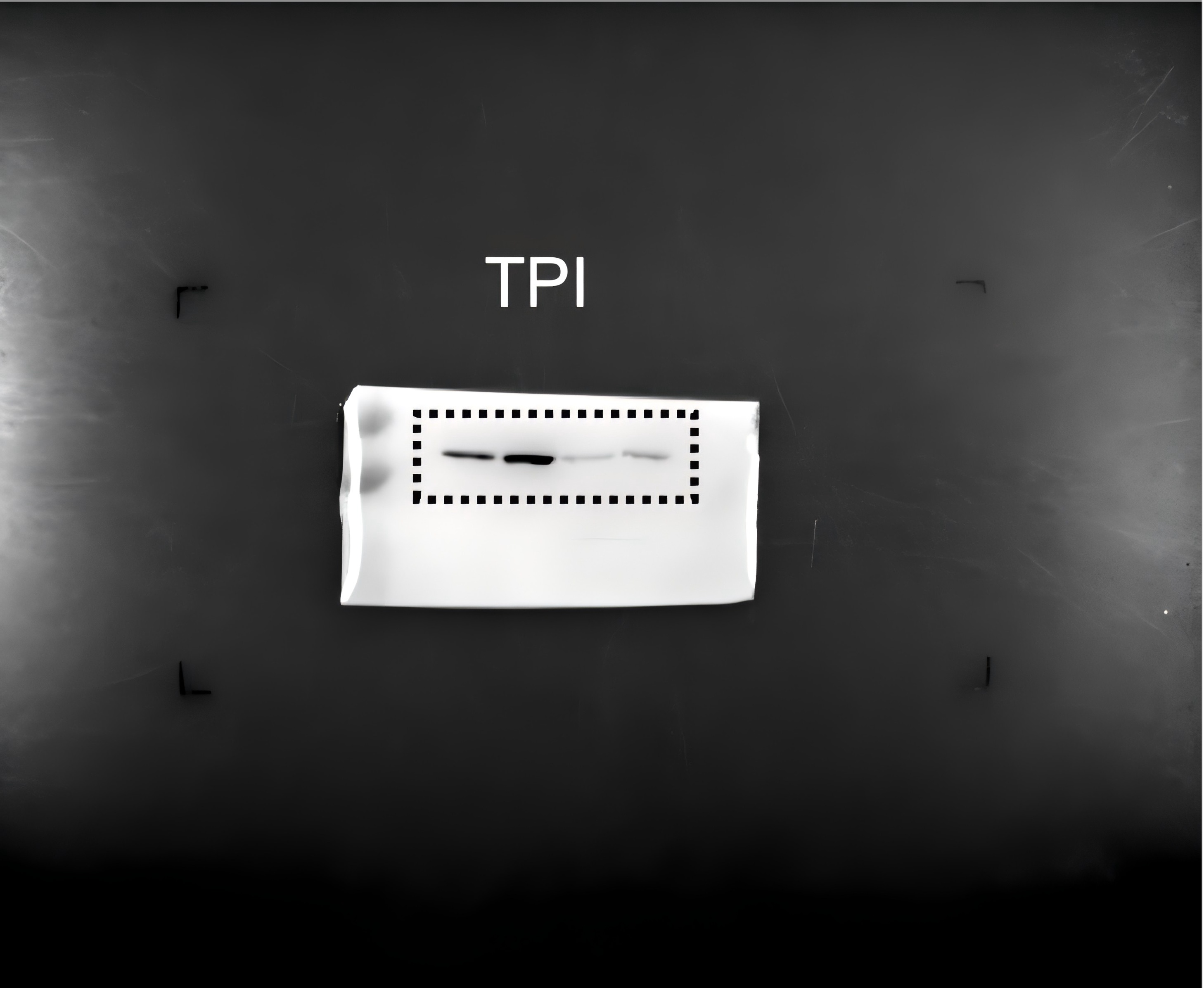

Supplement: Supplementary file 3 — Original Data [file 41419_2026_8662_MOESM3_ESM.zip › Original Data/Fig. S1/5-TPI.tif]

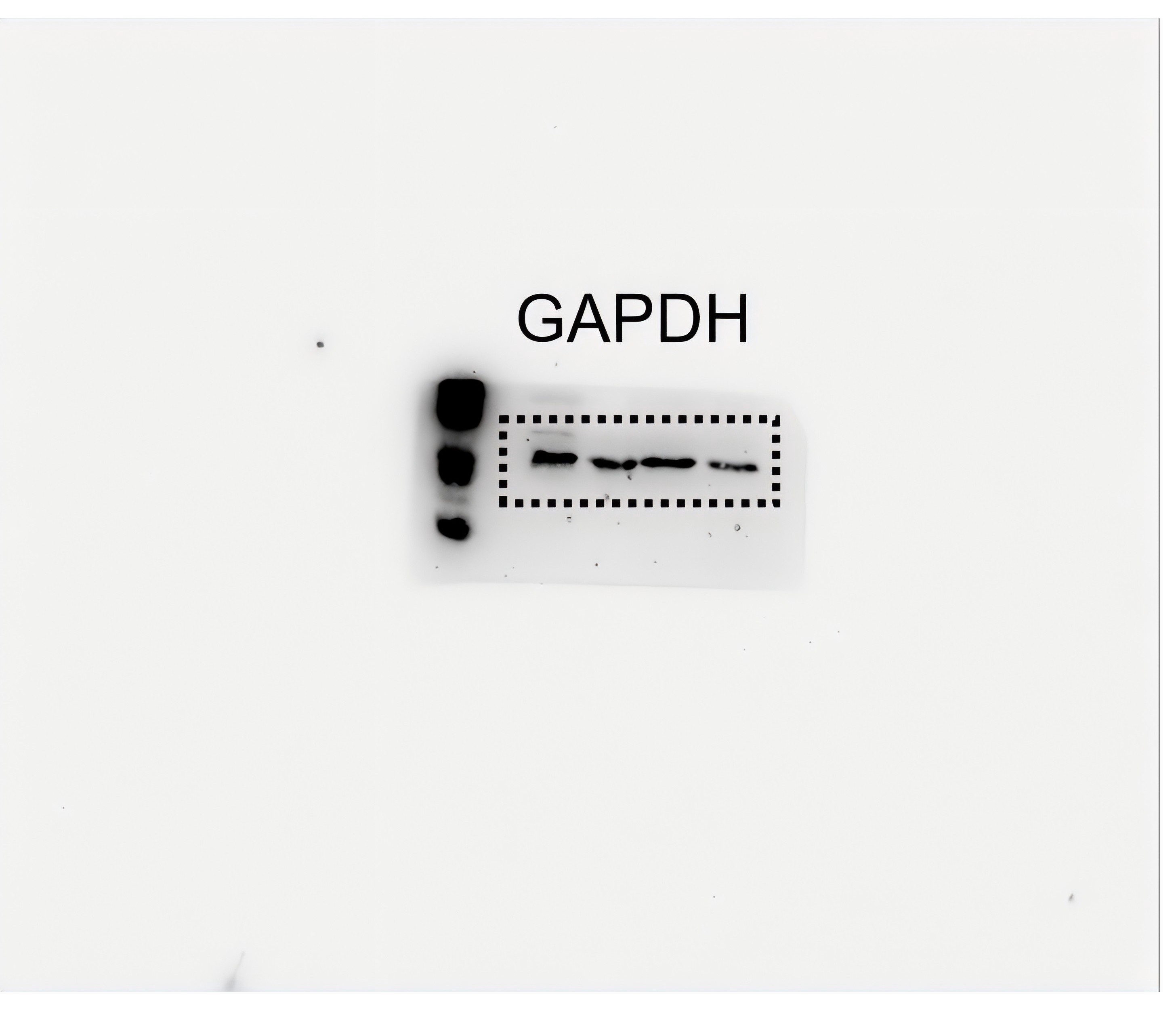

Supplement: Supplementary file 3 — Original Data [file 41419_2026_8662_MOESM3_ESM.zip › Original Data/Fig. S1/6-GAPDH.tif]

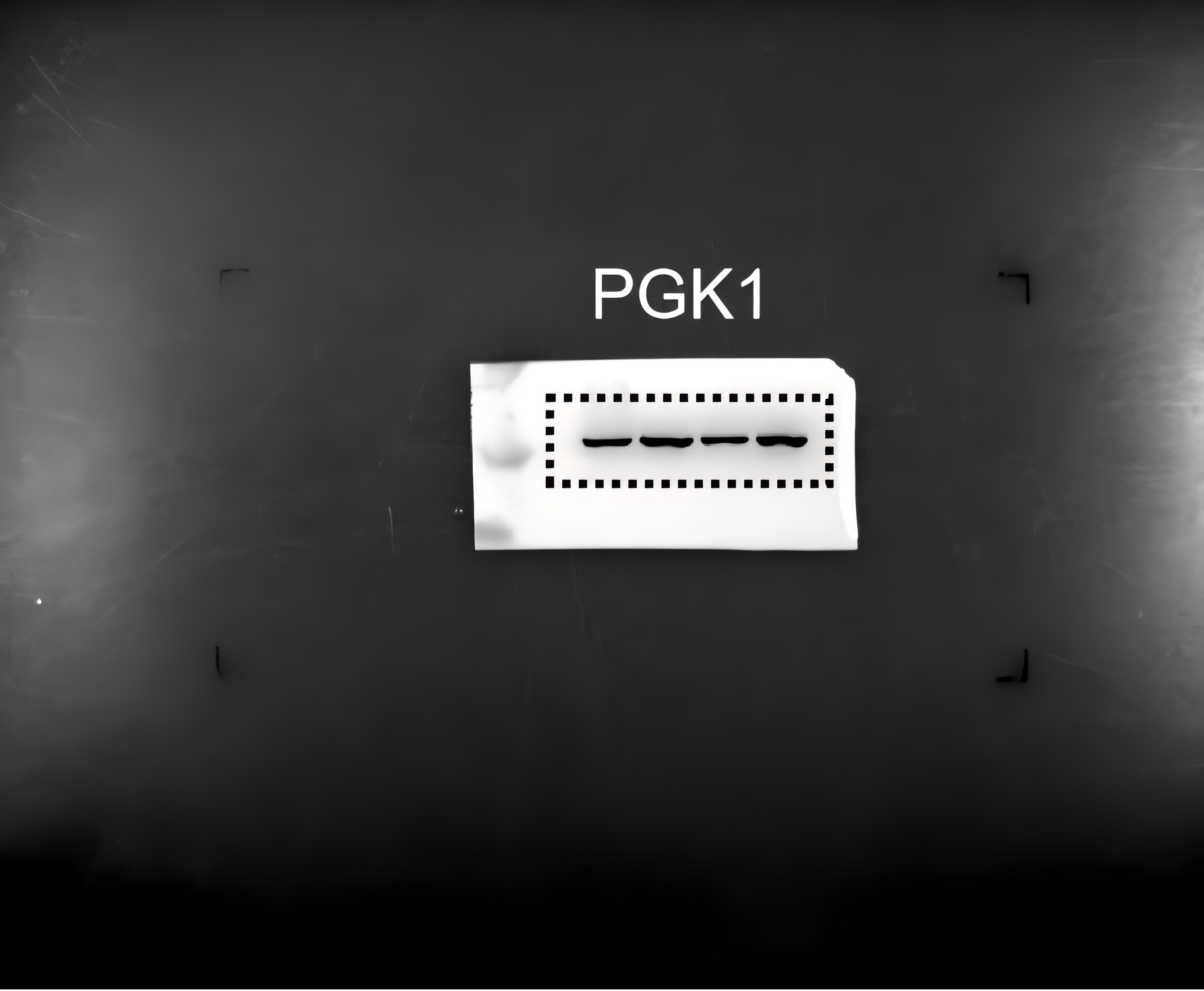

Supplement: Supplementary file 3 — Original Data [file 41419_2026_8662_MOESM3_ESM.zip › Original Data/Fig. S1/7-PGK1.tif]

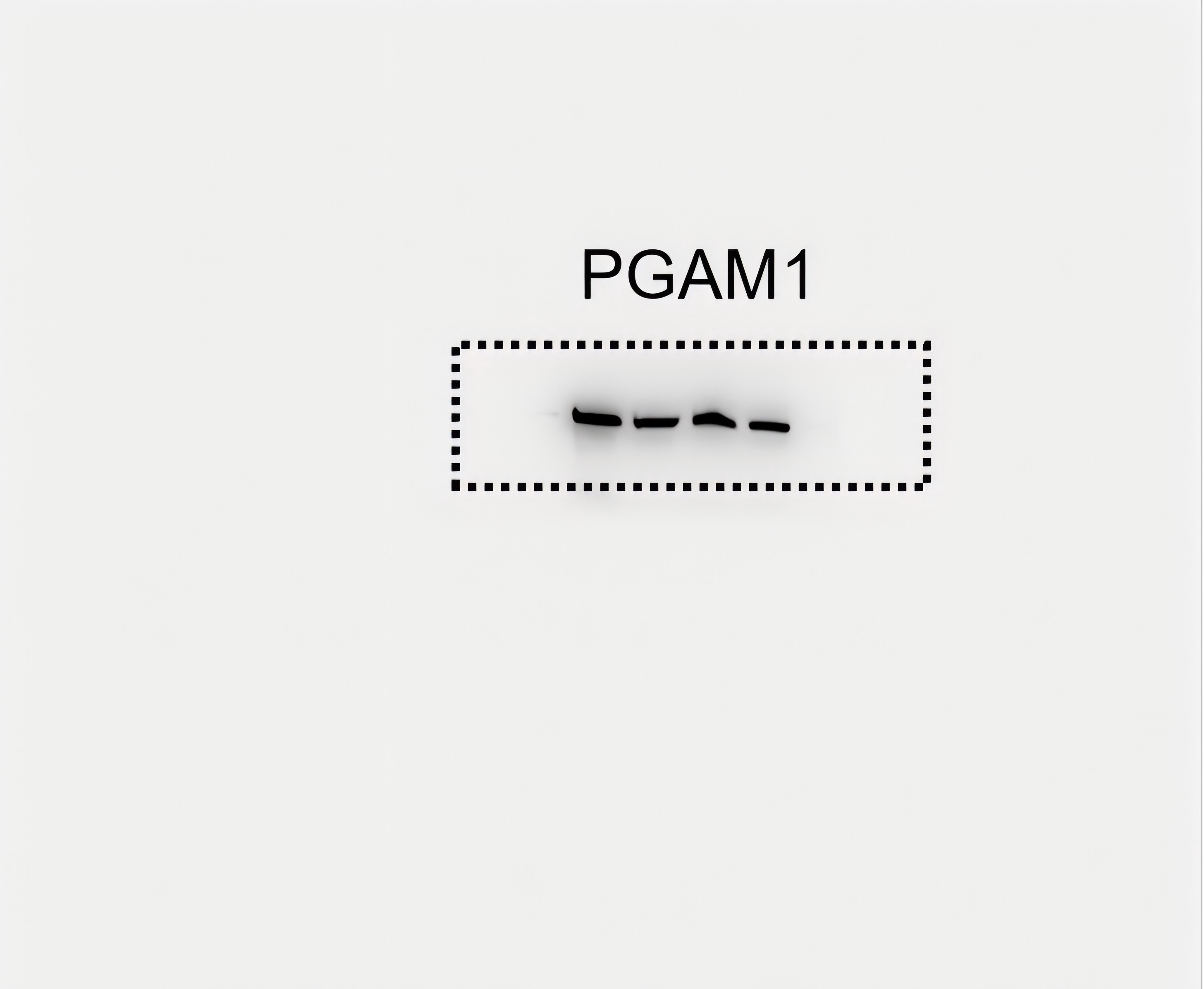

Supplement: Supplementary file 3 — Original Data [file 41419_2026_8662_MOESM3_ESM.zip › Original Data/Fig. S1/8-PGAM1.tif]

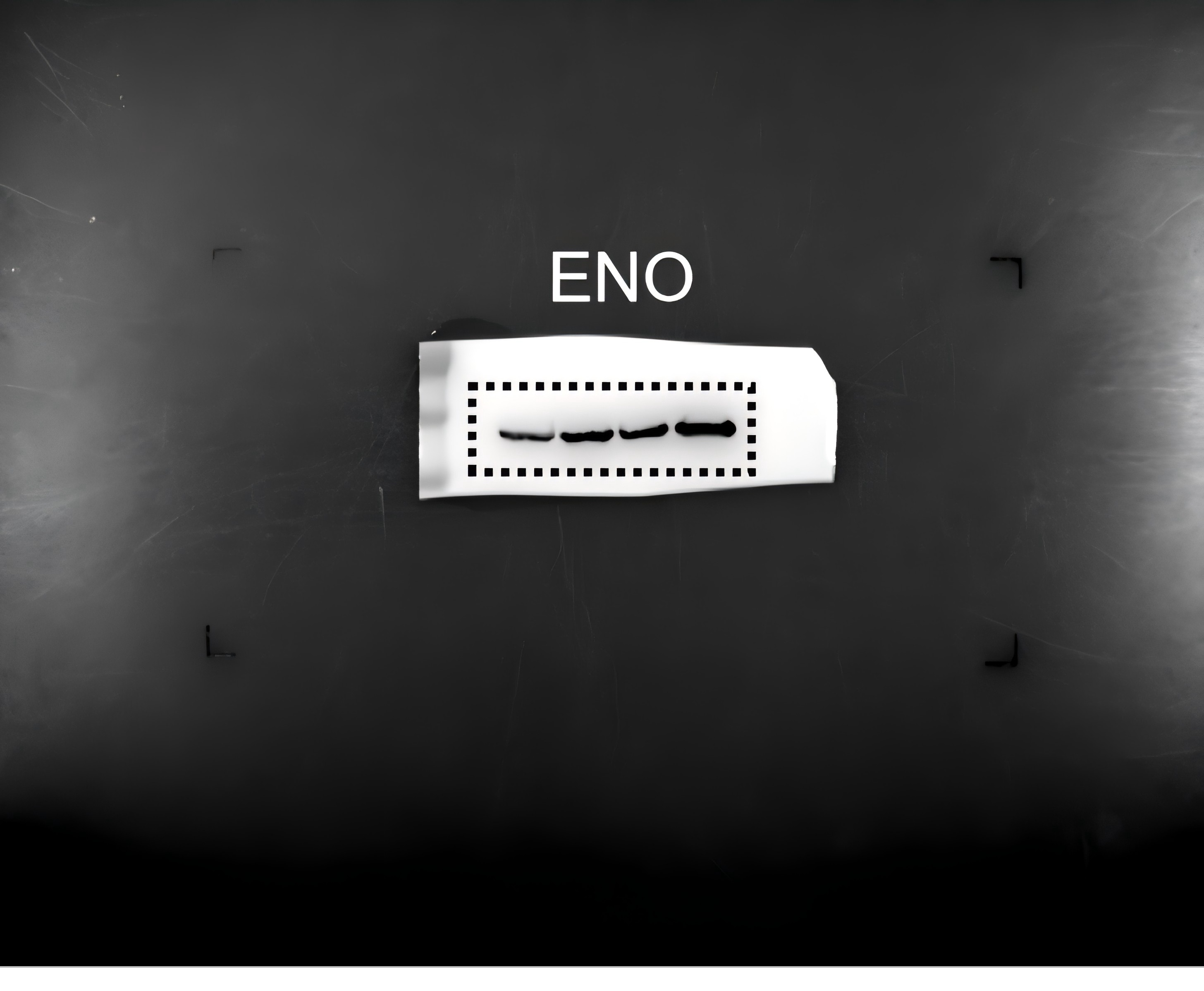

Supplement: Supplementary file 3 — Original Data [file 41419_2026_8662_MOESM3_ESM.zip › Original Data/Fig. S1/9-ENO.tif]
